# An unintended consequence of COVID-19 immunity passports—quasi-experimental evidence of moral hazard observed after implementing the domestic Green Pass policy during the second wave of the COVID-19 pandemic in Italy

**DOI:** 10.3389/fpubh.2024.1345119

**Published:** 2024-04-17

**Authors:** Cristina Oliva

**Affiliations:** ^1^Kingston Business School, Kingston University, Kingston upon Thames, United Kingdom; ^2^Kingston Business School, Faculty of Business and Social Sciences, Institute for Leadership and Management in Health, Kingston University, Kingston upon Thames, United Kingdom

**Keywords:** public health policy, unintended consequences, moral hazard, COVID-19, immunity certificates, Green Pass policy

## Abstract

**Objectives:**

Amidst the second wave of the COVID-19 pandemic, Italian policymakers mandated to exhibit evidence of vaccination or immunity (the Green Pass) as a condition to access retail premises and public offices. This study aims to offer evidence, in a quasi-experimental setting, suggesting that an unintended consequence of this policy was the emergence of moral hazard.

**Methods:**

Google visit duration data measured the time customers typically spend on retail premises or public offices. A pairwise comparison of median visit time per premise was performed at a six-week interval before and after the introduction of the Green Pass.

**Results:**

This study is the first to provide evidence of “ex-post” moral hazard associated with introducing a domestic Green Pass policy. The median visiting time on premises that required digital immunity control significantly increased after introducing the domestic Green Pass policy, contrary to other public premises where access remained free of limitations. The increase in median visit time in premises with faster customer turnaround, such as coffee shops (+49%) and fast-food restaurants (+45%), was lower than the increase observed for fine-dining restaurants (+74%) and pizzerias (+163%). No significant increase in median visit time was observed in premises where the Green Pass was not required, such as food supermarkets, retail non-food shops, post offices, banks, pharmacies, and gas stations.

**Conclusion:**

The evidence of moral hazard highlights the critical issue of unintended consequences stemming from public health policies. This discovery is pivotal for policymakers, indicating that unforeseen behavioral adjustments could offset the intended benefits despite the intent to reduce risk through measures like the Green Pass.

## Introduction

Public health policies throughout the COVID-19 pandemic were characterized by rapid and decisive actions aimed at combining efforts to contain the spread of the disease and mitigate its impacts. The primary goal was to delay the pandemic’s peak, ensure a more level distribution of the demand on limited healthcare resources, and protect vulnerable groups (1). The strict enforcement of policies in this unique situation also uncovered disagreements and showed how these decisions led to opinion differences among policymakers and the general public (2).

Given the significant changes brought about by the COVID-19 pandemic and its profound effects on societal norms, digital proof of immunity rapidly emerged as a contentious point of deliberation within most liberal democracies (3). The Green Pass, as it was commonly called in Italy, was an entry permit to public premises or facilities, a digital proof that an individual had either been vaccinated against COVID-19, received a negative test result, or recovered from COVID-19 (4).

Advocates emphasized that the Green Pass could potentially enhance freedom of movement, stimulate economic resurgence, and facilitate unhindered access to employment and educational avenues without compromising public health. Conversely, concerns abounded regarding their potential to precipitate unequal treatments, accentuate existing societal disparities, infringe on individual privacy rights, and inadvertently jeopardize public health by fostering complacency. An evolving body of academic work has begun to interrogate these ethical dimensions, offering a nuanced exploration of the advantages and pitfalls of such measures (5–7). Unintended responses to public health policies could lead to a maleficent “paradox effect” when riskier behaviors stem from heightened confidence (8).

Under severe epidemiological, economic, and social pressures, Italian policymakers began to explore the idea of a domestic Green Pass policy aimed at increasing the number of activities that could be subject to the possession of proof of vaccination or immunity. Therefore, since August 6, 2021, individuals showing their Green Pass would have complete freedom of access to indoor leisure activities such as restaurants, cafeterias, coffee shops, sports events, shows, museums, cultural exhibitions, swimming pools, gyms, and recreational facilities (9). The introduction of the domestic Green Pass policy was controversial, raising fierce media and political debates about its constitutional validity, practical impact on public health, respect for data privacy, and limitations of personal freedom (10).

The Green Pass domestic policy rests on a single epidemiological premise: individuals vaccinated or previously infected with COVID-19 who produce antibodies to the virus will then be immune to re-infection (at least for some nontrivial length of time) (11). Under this epidemiological condition, limiting access to public premises for Green Pass holders would create a sort of safe “immunity bubble” where the close contact risk of getting infected by COVID-19 would be virtually equal to zero. The Green Pass would implicitly signal to the community that the certificate holders were safe and others would be safe around them.

This study examines how the perceived “immunity” against COVID-19 risks possibly reduced risk-mitigating behaviors (ex-ante moral hazard). In economics, a moral hazard is a situation in which an economic actor has an incentive to increase its exposure to risk because it does not bear the full costs of that risk (12). In the COVID-19 infectious disease context, moral hazard applies where individuals who possess a certificate of immunity, such as the Green Pass, may relax protective behaviors, consequently increasing chances of close contact exposure to COVID-19 (13).

The study’s main aim is to provide quasi-experimental evidence of moral hazard determined by the certification of immunity by measuring differences in median visit duration by public premises and the consequent change in protective behavior observed among the holders before and after the introduction of the Green Pass.

The rationale behind the retrospective policy analysis of the domestic Green Pass implementation in Italy hinges on a single pivotal consideration. Understanding how the introduction of the Green Pass influenced individual and collective behaviors, the study seeks to assess whether the Green Pass motivated adherence to health measures or inadvertently led to complacency. The study is positioned to inform future policy adaptations where behavioral choices under moral hazard are rational and can be anticipated “ex-ante.”

## Methods

### Close contact risk of exposure to COVID-19

COVID-19 spreads mainly among people in close contact (14). When defining close contact, factors include proximity (closer distance likely increases exposure risk) and exposure duration (longer exposure time likely increases exposure risk). A working definition of the risk of exposure to COVID-19 for daily activities was developed based on the CDC’s definition of close contact (15):


(1) Risk of exposure=crowding×visit duration

As recommended by the CDC, close contact should generally be determined irrespective of whether the contact was wearing respiratory personal protective equipment (PPE).

In Italy, maximum crowding standards are regulated by norms, which set the maximum number of people allowable for design purposes for each square meter of floor area concerning various categories of public offices and retail premises. In March 2020, a decree from the Prime Minister introduced urgent actions to mitigate the impact of the COVID-19 epidemiological crisis. It set a new maximum occupancy limit for all commercial premises based on the requirement to maintain a one-meter distance between individuals for social distancing (16). Consequently, since the introduction of the domestic Green Pass policy in August 2021, Equation (1) could be rewritten as the product of a constant (Kc) and visit duration Equation (2):


(2) Risk of exposure=Kc×visit duration

### Data collection and inclusion

Google data was used to measure visit duration, the time customers typically spend on a specific retail premise or public office. Google uses aggregated and anonymized data from users who have opted for Google Location History. Data on visit duration indicate the average amount of time (in minutes) customers spend in a particular location, such as a restaurant, coffee shop, or supermarket. These estimates are derived from analyzing patterns in customer visits over the preceding weeks. No personally identifiable information, such as an individual’s location, contact, or movement, will be made available at any point (17).

Visit duration data was collected from all the Genoa metropolitan area retail activities visible on Google Maps and reported visit duration times. Interpreting mobility data in metropolitan areas required an in-depth understanding of urbanism and road mapping in the selected area. The choice of location was determined by the fact that the author was born and raised in a metropolitan area of Genoa. This methodological choice was consistent with Google’s recommendation to avoid comparing places across regions because of local differences in the data, which might be misleading (18).

Visit duration times (in minutes) for individual premises located by Google Maps in the metropolitan area of Genoa, Italy, were then aggregated into median visit duration time (in minutes) by ten categories according to their primary use: coffee shops, fast food restaurants, pizzerias, fine-dining restaurants, food supermarkets, retail non-food shops, post offices, banks, pharmacies, and gas stations.

Two main factors informed the choice of the time interval between the two observations. The first was the date of introduction of the domestic Green Pass policy (August 6, 2021), which could not be anticipated ex-ante. The second was the availability of a convenience sample of visit duration data dated six weeks before the introduction of the Green Pass policy (June 28, 2021). Visit duration data had been collected following a method perfectly consistent with the one adopted for the second observation, and the data set had been published (19). Based on the date of the first implementation of the domestic Green Pass in Italy and the availability of a convenience sample collected six weeks before, the second sample of visit duration data was collected six weeks after the introduction of the domestic Green Pass (September 13, 2021).

**Figure 1:**
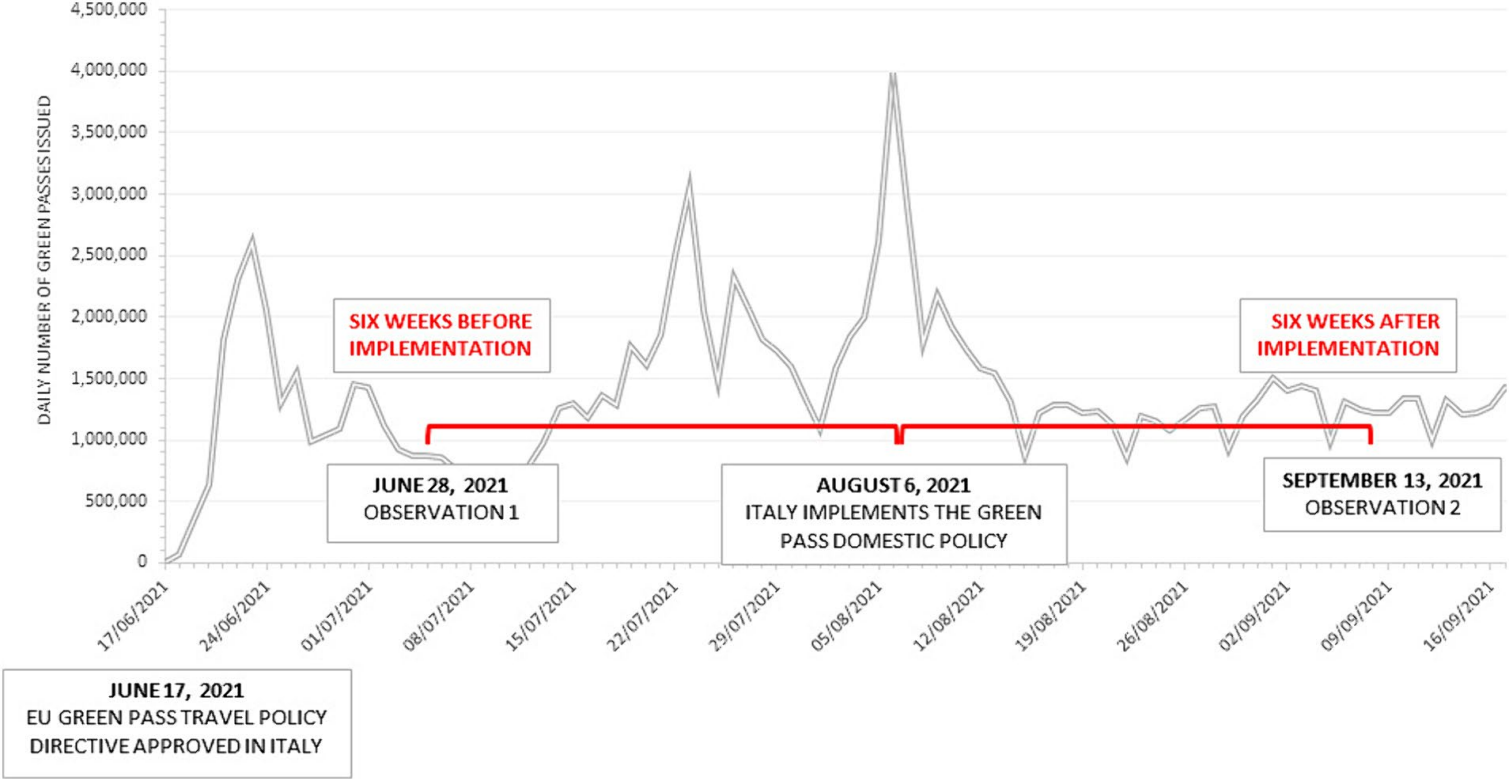
The time interval between the two observations. The time chart shows the number of Green Passes daily issued by the Italian Government from adopting the EU directive concerning travel passes (June 17, 2021) to the end of September 2021 (source: Italian Ministry of Health repository). On August 6, 2021, the domestic policy of Green Pass was first implemented. Unlike the travel pass needed to travel abroad, the domestic policy mandated the Green Pass as a condition for all individuals to access crowded retail premises (coffee shops, fast foods, pizzerias, and fine-dining restaurants). The time interval of data collection for the visit time duration for the two observations was set six weeks before and after the introduction of the domestic Green Pass policy in Italy.

Visit duration data were manually transcribed from Google Maps during two specific working weeks, with data collected within five consecutive days: from Monday, June 28th to Friday, July 2nd, 2021 (Observation 1) and from Monday, September 13th to Friday, September 17th, 2021 (Observation 2). The dates of the two observations spanned the summer season, reducing the bias of seasonality, which could have impacted visit duration and, consequently, changes in customers’ behavior. This aspect is particularly relevant to the location of the study: Genoa, a medieval city on the Italian Riviera, is a popular resort rich in art and museums, with an evocative old town, a varied food and wine culture, and a sprawling seafront (Figure 1).

Google determines peak hours, expected wait times, and the length of visits by utilizing aggregated and anonymized data from users who have activated Google Location History. The average visit duration was displayed if a business receives sufficient visits from these users. This data will only appear if enough visitation data is available for that business through Google (20). Due to this limitation, the list of retail premises whose visit duration data were collected in the second observation did not match the list of premises included in the first observation. This discrepancy could lead to a methodological bias since premises grouped in the same cluster can have different features that can significantly impact visit time duration. For example, a coffee shop can have a bar counter and a few tables where the customers quickly consume an espresso or a soft drink. Another coffee shop can have a patisserie and a vast seating area, inviting customers to a significantly longer visit time. Due to this limitation, there were discrepancies between retail premises with visit duration data for the first and second observation. Only retail premises with data for the first and second observations were included for analyses to reduce potential bias and ensure consistency between observations.

In normal distribution, the mean value per cluster would be used as a variable to be compared between observations. In contrast, the median value would have been the variable of choice in skewed distribution since outliers could distort the mean value (21). To reduce the bias of validity when including pairwise samples of premises showing different sizes (e.g., fine dining restaurant *n* = 34, and food supermarket *n* = 155), all mean/median values were resampled with replacement one thousand times (22).

The final sample was then clustered into ten groups of premises: four of which required the Green Pass (fine dining restaurants, pizzerias, fast food, and coffee shops) and six that did not require the Green Pass (food supermarkets, retail stores, banks, post offices, gas stations, and pharmacies).

The data collected for the study, including individual location data and a data dictionary defining each field in the set, are available in the Supplementary material.

### Hypothesis testing

As discussed earlier, since the introduction of the domestic Green Pass policy in Italy, the risk of exposure for each retail activity is dependent on a single variable: the visit duration time. Consequently, to test the moral hazard hypothesis, the following null hypothesis was formulated for each retail activity included in the sample:

*H_0_*: Visit duration times obtained six weeks before and after the introduction of the domestic Green Pass policy have the same means/medians.

*H_a_*: Visit duration times obtained six weeks before and after the introduction of the domestic Green Pass policy have different means/medians.

Suppose the null hypothesis H_0_ cannot be rejected for all retail activities or most activities requiring Green Pass; in this case, the conclusion would be that implementing the domestic policy in Italy did not generate moral hazard, as previously defined. If the null hypothesis is rejected, accepting the alternative hypothesis implies that the mean/median visit time duration differed between the two observations. Suppose the mean/median visit duration time related to the premises that required a Green Pass increased. In that case, while the mean/median duration time of the premises where the Green Pass was not required did not change, then moral hazard was the unintended consequence of the introduction of the domestic Green Pass and ultimately resulted in a higher close contact risk of COVID-19 infection for the holders.

### Data analysis

The choice of method for comparing mean/median visit duration time between the two observations will be informed by the normality test of each sample of data aggregated by premises. In the case of normal distribution of the data, a one-way analysis of variance (ANOVA) will be used to compare whether paired samples’ means are significantly different.

In case of skewed data distribution observed in each sample, medians will be first resampled with replacement (1,000 iterations). Then, the Mood test, a special case of Pearson’s chi-squared test, will be used to compare pairwise medians. The Mood test is a non-parametric method for comparing k independent samples (23). The null hypothesis is that the distributions of k groups are equal. The Mood test assumes independence of observations and no assumption of normality. If Mood’s median test result is significant, a post-hoc test will be conducted to investigate which medians differ (24).

XLSTAT statistical software for Excel by Addinsoft was used for resampling and statistical analysis.

## Results

### Significance of differences in median visit duration time by premise

**Figure 2:**
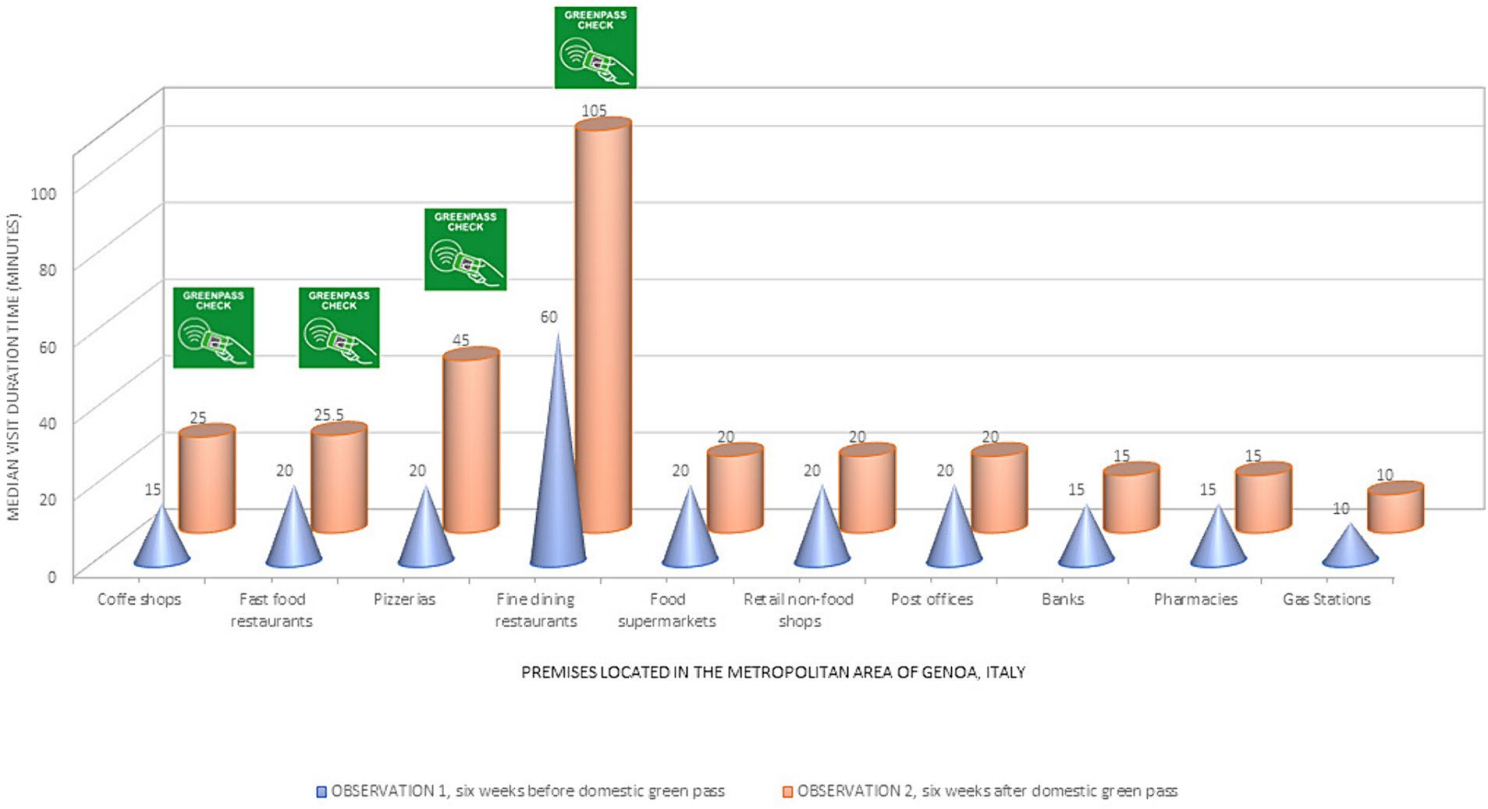
Median visit duration time for retail and public premises. The graph shows the median visit duration time (in minutes) obtained by Google Maps for the two samples of premises in the metropolitan area of Genoa, Italy, aggregated by main activity. The cones indicate the median visit time for the first observation (six weeks before the domestic Green Pass policy). The cylinders show the median visit time at the second observation (six weeks after the mandatory Green Pass). The label “Green Pass check” indicates the crowded premises where the pass was mandated as a condition to access (coffee shops, fast foods, pizzerias, and fine-dining restaurants).

The study included a total sample of 506 retail premises and public offices in the metropolitan area of Genoa, Italy. Typical visit duration time (in minutes) was reported by Google Maps and observed at two specific time points during the second half of 2021. The store data was then clustered into ten groups of premises according to their primary activity. A graphical representation of the pairwise comparison of the observed median visit time by premise seemed to indicate a significant increase in the average time spent by customers in the premises where the Green Pass was mandatory compared to the premises that did not require the Green Pass as a condition to access (Figure 2).

### Were these differences significant?

The normal distribution hypothesis was rejected for all data samples included in the analysis. Consequently, bootstrapped estimators of median values were obtained by resampling with 1,000 replacements for all samples included in the analysis. Moreover, the non-normality condition justified the choice of non-parametric tests, such as Mood’s tests, to compare the bootstrapped estimators of median values.

The four premises with conditional access to the exhibition of the Green Pass (namely, coffee shops, fast foods, pizzerias, and fine-dining restaurants) showed a significant increase in visit duration time compared to the one observed before its introduction (June 28, 2021). On the other hand, the remaining stores or offices that did not require the Green Pass showed no noticeable increase in the typical time spent inside the premises.

Based on the observed data and the results of Mood’s statistically significant differences in median values, the null hypothesis (H_0_) that visit duration times obtained six weeks before and after the introduction of the domestic Green Pass policy have the same medians can be rejected. The alternative hypothesis (H_a_) should be accepted: introducing the domestic Green Pass policy increased the median visit duration observed in the premises where possession was required.

Table 1 summarizes this study’s main results, while the complete statistical analysis is available in the Supplementary material.

**Table 1:** Change in median visit duration time by premise after the implementation of the Green Pass domestic policy.

Retail premises and public offices		Sample *n* = 506	Observation 1 (28 June – 02 July, 2021)			Observation 2 (13–17 September, 2021)			OBS 1 v OBS 2 significance of paired differences
			Median visit duration time (minutes)	Resampled median (1,000 iterations; significance level = 5%)	Shapiro–Wilk test of normality *p*-value two-tailed (α 0.05) (*)	Median visit duration time (minutes)	Resampled median (1,000 iterations; significance level = 5%)	Shapiro–Wilk test of normality *p*-value two-tailed (α 0.05) (*)	Mood test *p*-value (α 0.05) (**)
Green Pass required	Coffee shops	39	15.00	15.35	<0.0001	25.00	22.81	0.001	0.002
	Fast food restaurants	26	20.00	18.36	0.015	25.50	26.71	<0.0001	0.001
	Pizzerias	36	20.00	18.17	<0.0001	45.00	47.82	0.002	<0.0001
	Fine dining restaurants	32	60.00	60.09	<0.0001	105.00	104.81	0.000	<0.0001
Green Pass not required	Food supermarkets	155	20.00	17.98	<0.0001	20.00	18.64	<0.0001	0.877
	Retail non-food shops	44	20.00	20.84	0.001	20.00	20.86	0.000	0.823
	Post offices	60	20.00	18.01	<0.0001	20.00	18.95	<0.0001	0.798
	Banks	37	15.00	15.00	0.000	15.00	14.99	<0.0001	1.000
	Pharmacies	80	15.00	15.00	<0.0001	15.00	15.00	<0.0001	0.634
	Gas stations	26	10.00	10.00	<0.0001	10.00	10.00	<0.0001	1.000

(*) Shapiro–Wilk test interpretation: H0: The variable from which the sample was extracted follows a normal distribution. H_a_: The variable from which the sample was extracted does not follow a normal distribution. As the computed *p*-value is lower/higher than the significance level alpha = 0.05, one should reject/accept the null hypothesis H0, and accept/reject the alternative hypothesis H_a_. (**) Mood test interpretation: H0: The medians of Observation 1 and Observation 2 are equal. H_a_: Medians of Observation 1 and Observation 2 are not equal. As the computed p-value is lower/higher than the significance level alpha = 0.05, one should reject/accept the null hypothesis H0, and accept/reject the alternative hypothesis, H_a_.

### What are the implications of this finding for public health?

Table 2, reported below, shows the horizontal and vertical analysis of the change in visit duration, which provides valuable insights into the incremental risk of exposure to COVID-19 observed after introducing the domestic Green Pass policy in Italy.

**Table 2:** Change in relative and incremental risk of exposure by premise after introducing the Green Pass domestic policy.

Retail premises and public offices		Resampled median visit duration time (minutes)			Relative risk of exposure (gas stations = 1)		Incremental risk of exposure after introduction of the Green Pass
		Observation		Significance of paired differences *p*-value (α 0.05) (*)	Observation		OBS 2 v OBS 1(%)
		1	2		1	2	
Green Pass required	Coffee shops	15.35	22.81	0.002	1.54	2.28	49%
	Fast food restaurants	18.36	26.71	0.001	1.84	2.67	45%
	Pizzerias	18.17	47.82	<0.0001	1.82	4.78	163%
	Fine dining restaurants	60.09	104.81	<0.0001	6.01	10.48	74%
Green Pass not required	Food supermarkets	17.98	18.64	0.877	1.80	1.86	4%
	Retail non-food shops	20.84	20.86	0.823	2.08	2.09	0%
	Post offices	18.01	18.95	0.798	1.80	1.90	5%
	Banks	15.00	14.99	1.000	1.50	1.50	0%
	Pharmacies	15.00	15.00	0.634	1.50	1.50	0%
	Gas stations	10.00	10.00	1.000	1.00	1.00	0%

(*) Interpretation of Mood test of difference in paired medians: H_0_: The medians of Observation 1 and Observation 2 are equal. H_a_: Medians of Observation 1 and Observation 2 are not equal. As the computed *p*-value is lower/higher than the significance level alpha = 0.05, one should reject/accept the null hypothesis H_0_, and accept/reject the alternative hypothesis, H_a_.

A horizontal comparison of visit duration times observed in similar premises at different time intervals showed that by the end of June 2021, just six weeks after the introduction of the domestic Green Pass, the time typically spent by customers in pizzerias more than doubled while the time spent in fine dining restaurants increased by 74.42%. In addition, the duration of visits for more casual and frequent activities in our everyday lives, such as getting an espresso in a coffee shop or grabbing a burger in a fast food restaurant, increased by 48.60 and 45.48%, respectively. The vertical analysis confirmed the relevance of the changes in typical visit time to the risk of close contact in the premises where the Green Pass was required. Relative to gas stations (risk = 1), introducing the Green Pass determined a significant increase in exposure in the four activities already at the highest risk of close contact. The close contact risk increased for restaurants (from 6.01 to 10.48), pizzerias (from 1.82 to 4.78), fast foods (from 1.84 to 2.67), and coffee shops (from 1.54 to 2.28). Restaurants (of any kind), coffee shops, and bars did not require customers to wear facial protection (masks) when having a meal or a drink. Consequently, after introducing the domestic Green Pass policy, individuals paradoxically spent significantly more time on the premises that were most vulnerable to close contact risk.

On the other hand, the incremental and relative risk of exposure remained unchanged for all the premises where the Green Pass was not a condition of access.

Generalizing the outcomes by accepting the alternative hypothesis H_a_, this study provided the first evidence of moral hazard observed after introducing a domestic Green Pass policy.

The introduction of the Green Pass indicated that social activities should remain a key priority to contain the spread of COVID-19.

## Discussion

The COVID-19 pandemic has imposed an unprecedented social and economic burden on the global population. Although mass vaccination offers a promising exit strategy for the pandemic, limitations in personal freedom and social distancing have been enacted with varying degrees of severity at various points in time to contain the spread of the virus (25).

The benefits and challenges of the Green Pass remain controversial in the infection-acquired and vaccination-acquired immunity framework (26). In August 2021, Italy was the first mover to extend the remit of the Green Pass by enacting a domestic Green Pass policy to allow vaccinated individuals to return to their pre-COVID lives and do so safely. The domestic policy turned the Green Pass into proof of vaccination in a printed personal certificate or a digital version downloaded on a smartphone. As a result, the Green Pass became a mandatory prerequisite to attend particularly high-risk events in any indoor setting, whether a dinner in a restaurant, a movie theater, or a sports match.

This study is the first to provide evidence of “ex-post” moral hazard associated with introducing a domestic Green Pass policy. The median visiting time on premises that required digital immunity control significantly increased after the policy was introduced, contrary to other public premises where access remained free of limitations.

COVID Pass’s “ex-ante” impact on moral hazard is unambiguous: conceptually, the marginal disutility of risk-mitigating behavior (social distancing) should equal the marginal benefit of self-protection. The marginal benefit of self-protection is simply the marginal change in the probability of infection times the difference in utility between the uninfected and infected states of the world (27). Since the Green Pass certifies immunity, it reduces the marginal disutility of health loss from infection virtually to zero, consequently reducing the incentives for self-protection. This substitution effect would argue that the COVID-19 domestic Green Pass policy should increase ex-ante moral hazard.

Policymakers could have anticipated the behavioral reaction of Green Pass holders to lifting any precaution while dining out or having coffee at a table in a coffee shop. Eating out is an essential part of the Italian lifestyle. Therefore, the prolonged closure of restaurants, followed by a severe limitation of their opening hours (takeaway and delivery only after 6 p.m.), generated a vast dissatisfaction in the population craving social contact after a full year of distancing. It was conceivable that, under the “immunity” premise, Green Pass holders would increase their typical visit duration in these premises since the utility gained from additional time spent in social activities was higher than the perceived risk (close to zero) of incremental close contact risk of COVID-19. Therefore, as shown by comparing median visit duration data, citizens did just that. It was a rational behavioral choice, perfectly predictable.

### Limitations

The research aims to establish the impact of the introduction of the domestic Green Pass on the duration of customers’ visits to various retail premises and public offices. While the study offers valuable insights into this topic, several potential limitations exist.

The study relies on Google data to measure visit duration, dependent on users opting for Google Location History. The sample may not be representative of the entire population. The data does not account for non-Google users or those who have turned off their location history, which might introduce bias. Only businesses with sufficient Google visitation data are included, potentially excluding numerous other businesses.

Data collection is limited to the metropolitan area of Genoa, Italy. Results might not be generalized to other cities or regions of Italy or countries with different sociocultural or economic contexts.

The two one-week observations took place over 12 weeks in the course of the summer season. Despite the intention to reduce seasonality bias, this timeframe might not fully capture the domestic Green Pass policy’s long-term effects or behavior changes during other seasons.

The study assumes that visit duration time directly correlates with exposure risk. However, factors such as airflow, sanitation practices, and individual behaviors during visits could also influence risk.

In summary, while the research provides evidence of the domestic Green Pass policy’s unintended effect on consumer behavior in Genoa, Italy, several limitations exist. These should be acknowledged when interpreting or using the results to inform decision-making.

## Conclusion

The study provides insight into the effects of the domestic Green Pass on visit durations within certain premises and the subsequent increase in exposure risk provide a critical lens through which to reassess and refine pandemic response strategies. Acknowledging a paradoxical increase in exposure risk despite implementing a safety policy highlights the complexity of managing public health in the context of social and economic activities.

This finding is crucial for policymakers, suggesting that while policies like the Green Pass are designed to mitigate risk, they must also consider potential behavioral changes that could offset their benefits. Policymakers could have foreseen the “ex-ante” moral hazard consequent to implementing the domestic Green Pass policy in Italy and could have observed “ex-post” the unintended behavioral changes determined by the policy. The domestic Green Pass policy depended on the immunological condition of acquired immunity. When this condition was violated, the observed moral hazard significantly increased the close contact risk of infection caused by COVID-19 variants for the entire community. The World Health Organization (WHO) also suggested that the Green Pass could increase the risks of continued transmission because those carrying one would ignore public health advice about physical distancing. Vaccinated people could still be able to spread the virus and put others at risk, so experts have stressed the importance of continuing to distance and wear masks (28).

The balance between economic activity and health safety, as well as the call for continuous monitoring and adjustment of policies, further underlines the ongoing challenges in public health policymaking. These aspects of the study’s implications suggest that effective COVID-19 containment requires a multifaceted approach that includes initial policy implementation and continuing assessment and adaptation. To sustain Green Pass’ social and economic benefits, the risk of moral hazard could have been mitigated using Google visit duration data to inform the public and potentially influence social distancing decisions during public health crises.

The pandemic has revealed the importance of developing a shared awareness of threats for resilience in interconnected societies. This collective understanding encourages individuals to collaborate on common goals and mitigate shared dangers. A shared understanding of what constitutes a threat versus a desirable outcome may depend on how risk is communicated (29).

The COVID-19 health crisis has led to an unprecedented use of surveillance measures from public health authorities. The acceptance of the use of smartphone location data could mitigate the unintended consequences of moral hazard by helping people self-regulate their behavior to align with societal norms and expectations, essentially surveilling themselves without the need for external oversight (30).

Suppose the public knows the average visit duration for specific locations, such as coffee shops or restaurants. In that case, they can make informed decisions about the incremental risk of exposure of indulging in a conversation while sipping a cappuccino or having a three-course gourmet meal with a large group of friends. Moreover, suppose a store has a notably long visit duration. In that case, some might interpret that as potential inefficiency in social distancing measures and choose to visit at off-peak times or select another location.

In conclusion, the unintended consequences of future public health policies during a crisis can be mitigated by paying closer attention to the data, promoting transparency, encouraging participatory governance, and embracing innovative solutions, all while safeguarding privacy and advancing equity.

## Data availability statement

The original contributions presented in the study are included in the article/Supplementary material, further inquiries can be directed to the corresponding author.

## Ethics statement

Ethics approval and/or informed consent were not sought for this study. Publicly available Google data were collected to measure the time customers typically spend on a specific retail premise or public office. Google uses aggregated and anonymized data from users who have opted for Google Location History to determine visit duration. No personally identifiable information, such as an individual’s location, contact, or movement, will be made available at any point.

## Author contributions

CO: Conceptualization, Data curation, Formal analysis, Investigation, Methodology, Project administration, Visualization, Writing – original draft, Writing – review & editing.
