# Supplementary material for: An unintended consequence of COVID-19 immunity passports—quasi-experimental evidence of moral hazard observed after implementing the domestic Green Pass policy during the second wave of the COVID-19 pandemic in Italy
Source: Front Public Health. 2024 Apr 17;12:1345119. doi: 10.3389/fpubh.2024.1345119 (PMC11061360; doi:10.3389/fpubh.2024.1345119)
Supplement: Supplementary file 1 [file Data_Sheet_1.docx]

Online Supplementary Material

Table of Contents

[Definition of close contact risk of Covid-19 3](#_Toc148003503)

[References: 3](#_Toc148003504)

[Maximum crowding standard by retail activity 4](#_Toc148003505)

[UNI 10339 norm. 4](#_Toc148003506)

[DPCM anti Covid-19 4](#_Toc148003507)

[References: 6](#_Toc148003508)

[Mean visit duration in retail shopping premises: the Google Maps data 7](#_Toc148003509)

[Visit duration 7](#_Toc148003510)

[Sample database and statistical analysis 8](#_Toc148003511)

# Definition of close contact risk of Covid-19

The risk of getting COVID-19 is evolving daily and varies between and within communities. Spread happens when an infected person coughs, sneezes, or talks, and droplets from their mouth or nose are launched into the air and land in the mouths or noses of people nearby. The droplets can also be inhaled into the lungs. [1]

COVID-19 spreads mainly among people who are in close contact [1].

In our research, I used the CDC’s definition of close contact, revised on October 21^st^, 2020:

“Close Contact: someone who was within 6 feet of an infected person for a cumulative total of 15 minutes or more over a 24-hour period* starting from 2 days before illness onset (or, for asymptomatic patients, 2 days prior to test specimen collection) until the time the patient is isolated.” [2]

** Individual exposures added together over a 24-hour period (e.g., three 5-minute exposures for a total of 15 minutes). Data are limited, making it difficult to precisely define “close contact;” however, 15 cumulative minutes of exposure at a distance of 6 feet or less can be used as an operational definition for contact investigation. Factors to consider when defining close contact include proximity (closer distance likely increases exposure risk), the duration of exposure (longer exposure time likely increases exposure risk), whether the infected individual has symptoms (the period around onset of symptoms is associated with the highest levels of viral shedding), if the infected person was likely to generate respiratory aerosols (e.g., was coughing, singing, shouting), and other environmental factors (crowding, adequacy of ventilation, whether exposure was indoors or outdoors). Because the general public has not received training on proper selection and use of respiratory PPE, such as an N95, the determination of close contact should generally be made irrespective of whether the contact was wearing respiratory PPE. At this time, differential determination of close contact for those using fabric face coverings is not recommended.*

From the definition of closed contact I derived the working definition of ***closed contact risk of Covid-19*** for retail activities:

Crowding rate/square meter x median visit duration (minutes)

10.4 square meters* 15 minutes

* Equivalent to the area of a circle of 6 feet (1.82 cm) radius

## References:

[1] El Hassan M, Assoum H, Bukharin N, Al Otaibi H, Mofijur M, Sakout A. A review on the transmission of COVID-19 based on cough/sneeze/breath flows. Eur Phys J Plus. 2022;137(1):1. doi: 10.1140/epjp/s13360-021-02162-9.

[2] Centers for Disease Control and Prevention (CDC). Social distancing. Available online at: <https://www.cdc.gov/coronavirus/2019-ncov/prevent-getting-sick/social-distancing.html> . Last accessed on 21/12/2020

[3] Centers for Disease Control and Prevention (CDC). Appendix A – Glossary of Key Terms. Available online at: <https://www.cdc.gov/coronavirus/2019-ncov/php/contact-tracing/contact-tracing-plan/appendix.html#contact> Last accessed on 21/12/2020

# Maximum crowding standard by retail activity

## UNI 10339 norm.

The UNI 10339 standard applies to all aeraulic systems intended for the well-being of people, installed in buildings residential and non-residential intended to control thermal, hygrometric, quality and air movement in indoor environments [3]

Appendix A, with reference to various categories of buildings, provides the values ​​of the crowding indices (i.e. the number of people present, for design purposes, for each square meter of floor area). The crowding rates are intended to be conventional. They are fixed only for rooms where people are expected to stay.

## DPCM anti Covid-19

In order to guarantee the resumption of activities, following the lockdown phase, compatibly with the trend of the epidemic, as well as the protection of the health of the staff and users, a gradual and progressive remodelling of the containment measures was necessary.

As a consequence, the Prime Ministerial Decree (DPCM) of April 26, 2020, defined that the maximum crowding standard attributed to commercial establishments was 13.3 m^2^ per person (example: 3 people can enter a 40 m^2^ room). The text of the Decree reads verbatim: "For premises up to forty square meters, one person can access at a time, in addition to a maximum of two operators" (i.e. 40 sqm / 3 = 13.3 sqm). [4]

In May 2020, the National Institute for Occupational Accident Insurance (INAIL) produced a technical document in collaboration with the Istituto Superiore di Sanità (ISS) with the aim of providing technical evaluation elements to the political decision-maker about the possible remodelling of the measures to contain the infection from Covid-19, with the aim of guaranteeing the health and safety of both operators and consumers. INAIL published a technical document dedicated to coffee shops and restaurants, in which it established the standard of 4 square meters per person. [5]

The following Table reports the maximum crowding by retail activity allowed by both standards: UNI (before Covid-19) and the DPCM dated April 26,2020 (after Covid-19).

| **RETAIL ACTIVITIES** | **Crowding (people in the contact area)** | |  |  |
| --- | --- | --- | --- | --- |
|  | **Max crowding standard (people per square meter) UNI10339, October 2008** | **Max crowding standard (people per square meter) DPCM anti Covid-19, April 2020** |  |  |
| **Fine-dining restaurants** | 0.66 | 0.250 |  |  |
| **Pizza restaurants** | 0.66 | 0.250 |  |  |
| **Pubs** | 0.66 | 0.250 |  |  |
| **Fast-food restaurants** | 0.66 | 0.250 |  |  |
| **Coffee shops** | 0.80 | 0.250 |  |  |
| **Gyms** | 0.25 | 0.250 |  |  |
| **Hair salons** | 0.20 | 0.200 |  |  |
| **Shopping centres** | 0.20 | 0.200 |  |  |
| **Retail shops (non-food)** | 0.20 | 0.075 |  |  |
| **Food supermarkets** | 0.20 | 0.075 |  |  |
| **Pharmacies** | 0.20 | 0.075 |  |  |
| **Banks** | 0.20 | 0.075 |  |  |
| **Post offices** | 0.20 | 0.075 |  |  |
| **Gas stations *** | 0.20 | 0.075 |  |  |
| ** Max crowding standard refers to retail premises of the gas station (convenience store)* | | | | |

## References:

[3] Standard UNI 10339. Appendix A. Version updated on 17/10/2008. Available online at: [http://www.ctslab.eu/doc/Revisione_UNI_10339_del_081017.pdf Last accessed on 21/12/2020](http://www.ctslab.eu/doc/Revisione_UNI_10339_del_081017.pdf%20Last%20accessed%20on%2021/12/2020)

[4] Gazzetta Ufficiale. Prime Ministerial Decree (DPCM) of April 26, 2020. Available online at: <https://www.gazzettaufficiale.it/eli/id/2020/04/27/20A02352/sg> Last accessed on 11/01/2021

[5] National Institute for Occupational Accident Insurance (INAIL). Documento tecnico su ipotesi di rimodulazione delle misure contenitive del contagio da SARS-CoV-2 nel settore della ristorazione. May, 2020. Available online at: <https://marcigaglia.it/wp-content/uploads/2020/05/Documento-tecnico-ristorazione-2-compresso.pdf> Last accessed on 22/12/2020.

# Mean visit duration in retail shopping premises: the Google Maps data

## Visit duration

Since October 2020, Google made visit duration time available on Google Maps. This data shows how much time customers typically spend in a specific store. Visit duration estimates are based on patterns of customer visits over the past several weeks.

Visit duration is expressed in units of time (minutes ad hours)

Google reports median visit duration value in units of time (minutes), based on customer visits patterns over the past several weeks. When visit duration was shown as a range (e.g., 90–180 min), I consistently included the lower value in the sample only because more relevant to the determination of the evolution of close contact risk of exposure to COVID-19 over time. Data were collected from all the Genoa metropolitan area retail activities visible in Google Map and reporting visit duration time.


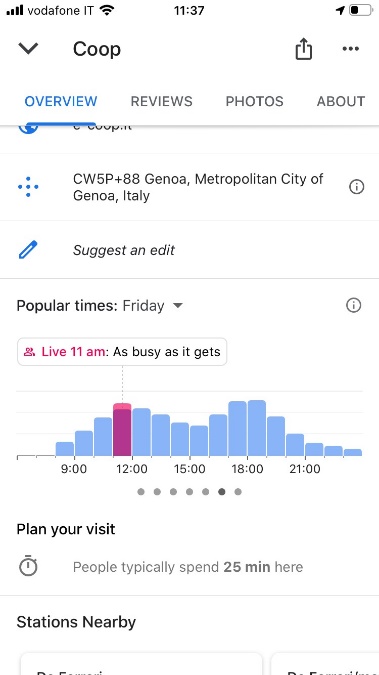

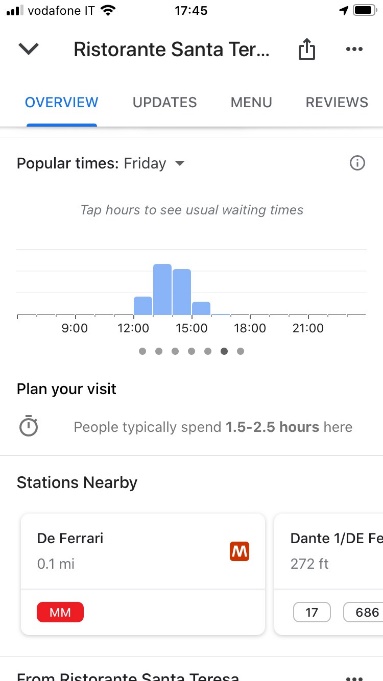


# Sample database and statistical analysis

| **Coffee shops** | **OBS 1** | **OBS 2** |
| --- | --- | --- |
| 23 Rosso | 15 | 20 |
| 7 Nasi | 15 | 30 |
| Bar Boasi | 20 | 25 |
| Bar Bolla | 15 | 25 |
| Bar Carioca | 15 | 20 |
| Bar Carletto 1960 | 15 | 15 |
| Bar Corallo | 15 | 20 |
| Bar Margherita | 10 | 30 |
| Bar Paolo e Gianni | 15 | 15 |
| Bar Tagliafico | 15 | 15 |
| Basilico Caffè Albaro | 15 | 30 |
| Bisquit Bar | 15 | 15 |
| Cafè HB 1969 | 20 | 30 |
| Caffè degli Specchi | 20 | 15 |
| Caffè Fogliotti | 15 | 20 |
| Caffetteria 68R | 10 | 20 |
| Caffetteria Carignano | 15 | 15 |
| Cafhein | 15 | 25 |
| Che Nervi | 15 | 15 |
| De Stefanis | 20 | 30 |
| Don Cola | 25 | 30 |
| Don Paolo | 20 | 25 |
| Douce Patisserie Cafè | 20 | 45 |
| East River Cafè | 15 | 15 |
| Giumin | 15 | 35 |
| La Bottega del Caffè | 25 | 25 |
| La Piazza | 20 | 30 |
| La Superba | 25 | 30 |
| Maca Caffè | 25 | 20 |
| Mangini | 15 | 25 |
| Marina Bar | 15 | 20 |
| Mentelocale Bistrot | 15 | 20 |
| Merdiana caffè | 25 | 30 |
| P&O Bar | 15 | 30 |
| Quinto Caffè | 15 | 20 |
| Simo Caffè | 20 | 20 |
| Storico Lounge Cafe | 30 | 45 |
| Tre Corone | 20 | 20 |
| Vintage Cafè 74 | 15 | 30 |

| **Descriptives** | **OBS 1** | **OBS 2** |  |  |  |  |  |  |  |
| --- | --- | --- | --- | --- | --- | --- | --- | --- | --- |
| Nbr. of observations | 39 | 39 |  |  |  |  |  |  |  |
| Nbr. of missing values | 0 | 0 |  |  |  |  |  |  |  |
| Obs. without missing data | 39 | 39 |  |  |  |  |  |  |  |
| Minimum | 10.000 | 15.000 |  |  |  |  |  |  |  |
| Maximum | 30.000 | 45.000 |  |  |  |  |  |  |  |
| Freq. of minimum | 2 | 8 |  |  |  |  |  |  |  |
| Freq. of maximum | 1 | 2 |  |  |  |  |  |  |  |
| Median | 15.000 | 25.000 |  |  |  |  |  |  |  |
| Mean | 17.564 | 24.231 |  |  |  |  |  |  |  |
| Variance (n-1) | 19.568 | 58.603 |  |  |  |  |  |  |  |
| Standard deviation (n-1) | 4.424 | 7.655 |  |  |  |  |  |  |  |
| Median absolute deviation | 0.000 | 5.000 |  |  |  |  |  |  |  |
|  |  |  |  |  |  |  |  |  |  |
|  |  |  |  |  |  |  |  |  |  |
| **Shapiro-Wilk test (OBS 1):** | |  |  |  |  |  |  |  |  |
|  |  |  |  |  |  |  |  |  |  |
| W | 0.813 |  |  |  |  |  |  |  |  |
| p-value (Two-tailed) | **<0.0001** |  |  |  |  |  |  |  |  |
| alpha  Coffee shops n=39*SEP*Summary statistics*SEP*$M$31 Coffee shops n=39*SEP*Results of the resampling (X1)*SEP*$M$37  Coffee shops n=39*SEP*Summary statistics*SEP*$M$59 Coffee shops n=39*SEP*Results of the resampling (X1)*SEP*$M$65 | 0.05 |  |  |  |  |  |  |  |  |
|  |  |  |  |  |  |  |  |  |  |
| Test interpretation: |  |  |  |  |  |  |  |  |  |
| H0: The variable from which the sample was extracted follows a Normal distribution. | | | | | | |  |  |  |
| Ha: The variable from which the sample was extracted does not follow a Normal distribution. | | | | | | |  |  |  |
| As the computed p-value is lower than the significance level alpha=0.05, one should reject  the null hypothesis H0, and accept the alternative hypothesis Ha. | | | | | | | | |  |
|  |  |  |  |  |  |  |  |  |  |
| RunProcRSP Form118.txt CheckBoxTrans,CheckBox,0,False,03,False,Trans,False,,, ListBoxQuanti,ListBox,,True,200000000000_Outputs,True,,False,,, CheckBoxHist,CheckBox,0,True,300000000000_Charts,True,Histograms,False,,, OptionButtonHistBar,OptionButton,-1,True,300000000100_Charts,True,Bars,False,,, OptionButtonHistCont,OptionButton,0,True,300000010100_Charts,True,Continuous line,False,,, CheckBoxCum,CheckBox,0,True,300000000200_Charts,True,Cumulative histograms,False,,, OptionButtonHisBased,OptionButton,-1,True,300000000300_Charts,True,Based on the histogram,False,,, OptionButtonECDF,OptionButton,0,True,300000010300_Charts,True,Empirical cumulative distribution,False,,, CheckBoxRData,CheckBox,0,True,200000000008_Outputs,True,Resamples,False,,, TextBoxConfPer,TextBox,95,True,200000000003_Outputs,True,Confidence interval (%):,False,,, CheckBoxRStat,CheckBox,0,True,200000000007_Outputs,True,Resampled statistics,False,,, CheckBoxNormInt,CheckBox,0,True,200000000004_Outputs,True,Standard bootstrap interval,False,,, CheckBoxPercInt,CheckBox,0,True,200000000005_Outputs,True,Simple percentile interval,False,,, CheckBoxBiasInt,CheckBox,0,True,200000000006_Outputs,True,B.C. percentile interval,False,,, OptionButton_MVRemove,OptionButton,0,True,100000000100_Missing data,True,Remove the observations,False,,, OptionButton_MVEstimate,OptionButton,0,True,100000000300_Missing data,True,Estimate missing data,False,,, OptionButton_MeanMode,OptionButton,-1,True,100000000400_Missing data,True,Mean,False,,, OptionButtonAll,OptionButton,-1,True,100000000200_Missing data,True,For all samples,False,,, OptionButtonRestrict,OptionButton,0,True,100000010200_Missing data,True,For the corresponding sample,False,,, OptionButtonMVRefuse,OptionButton,-1,True,100000000000_Missing data,True,Do not accept missing data,False,,, OptionButton_W,OptionButton,0,True,000000000001_General,True,Workbook,False,,, OptionButton_R,OptionButton,-1,True,000000010001_General,True,Range,False,,, OptionButton_S,OptionButton,0,True,000000020001_General,True,Sheet,False,,, RefEdit_R,RefEdit,'Coffee shops n=39'!$M$20,True,000000000101_General,True,Range:,False,,0,0 CheckBoxVarLabels,CheckBox,0,True,000000000201_General,True,Sample labels,False,,, CheckBox_W,CheckBox,0,True,000000000301_General,True,Weights,False,,, RefEdit_W,RefEdit,,True,000000000401_General,True,Weights:,False,,, TextBox_Resample,TextBox,1000,True,000000000000_General,True,Number of samples:,False,,, RefEdit_X,RefEdit0,'Coffee shops n=39'!$I$2:$I$40,True,000000000100_General,True,Quantitative data:,False,,39,1 ComboBoxMethod,ComboBox,0,True,000000010300_General,True,Method:,False,,, TextBox_obs,TextBox,5,True,000000000500_General,True,Sample size:,False,,, ComboBoxChartY,ComboBox,2,True,300000000101_Charts,True,Ordinate of the histograms:,False,,,  1 ListBox 118 ListBoxQuanti  1 22  Sum,0 Mean,-1 Variance (n),-1 Variance (n-1),-1 Standard deviation (n),-1 Standard deviation (n-1),-1 Median,-1 1st Quartile,0 3rd Quartile,0 Variation coefficient,0 Standard error of the mean,0 Mean absolute deviation,0 Median absolute deviation,-1 Geometric mean,0 Geometric standard deviation,0 Harmonic mean,0 1-Percentile,0 99-Percentile,0 2.5-Percentile,0 97.5-Percentile,0 5-Percentile,0 95-Percentile,0  777618 |  |  |  |  |  |  |  |  |  |
| **Shapiro-Wilk test (OBS 2):** | |  |  |  |  |  |  |  |  |
|  |  |  |  |  |  |  |  |  |  |
| W | 0.878 |  |  |  |  |  |  |  |  |
| p-value (Two-tailed) | **0.001** | 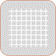 |  |  |  |  |  |  |  |
| alpha | 0.05 |  |  |  |  |  |  |  |  |
|  |  |  |  |  |  |  |  |  |  |
| Test interpretation: |  |  |  |  |  |  |  |  |  |
| H0: The variable from which the sample was extracted follows a Normal distribution. | | | | | | |  |  |  |
| Ha: The variable from which the sample was extracted does not follow a Normal distribution. | | | | | | |  |  |  |
| As the computed p-value is lower than the significance level alpha=0.05, one should  reject the null hypothesis H0, and accept the alternative hypothesis Ha. | | | | | | | | |  |
|  |  |  |  |  |  |  |  |  |  |
|  |  |  |  |  |  |  |  |  |  |
| **Results of the resampling (OBS 1):** | | |  |  |  |  |  |  |  |
|  |  |  |  |  |  |  |  |  |  |
| Parameters | Estimator | Estimator (Bootstrap) | Standard deviation (Bootstrap) |  |  |  |  |  |  |
| Mean | 17.564 | 17.595 | 0.679 |  |  |  |  |  |  |
| Variance (n)  RunProcMOO Form168.txt RefEditT,RefEdit,'Sheet1'!$I$1:$K$40,True,000000000100_General,True,,False,,40,3 RefEditGroups,RefEdit,,True,000000000300_General,True,Sample identifiers:,False,,, CheckBoxTrans,CheckBox,0,False,03,False,Trans,False,,, CheckBoxPairwise,CheckBox,-1,True,000000000301_General,True,Multiple pairwise comparisons,False,,, OptionButtonSample,OptionButton,-1,True,000000000600_General,True,One column per sample,False,,, OptionButtonVariable,OptionButton,0,True,000000000700_General,True,One column per variable,False,,, CheckBoxLabels,CheckBox,-1,True,000000000201_General,True,Column labels,False,,, OptionButton_W,OptionButton,0,True,000000020001_General,True,Workbook,False,,, OptionButton_R,OptionButton,-1,True,000000000001_General,True,Range,False,,, OptionButton_S,OptionButton,0,True,000000010001_General,True,Sheet,False,,, RefEdit_R,RefEdit,'Sheet1'!$M$63,True,000000000101_General,True,Range:,False,,0,0 TextBox_Conf,TextBox,5,True,100000010001_Options,True,Significance level (%):,False,,, CheckBox_Desc,CheckBox,-1,True,300000000000_Outputs,True,Descriptive statistics,False,,, OptionButtonAsympt,OptionButton,0,True,100000000101_Options,True,Asymptotic p-value,False,,, OptionButtonMonte,OptionButton,-1,True,100000000201_Options,True,Monte Carlo method,False,,, TextBoxPermut,TextBox,10000,True,100000010301_Options,True,Number of simulations:,False,,, CheckBoxCorrect,CheckBox,-1,True,100000000401_Options,True,Continuity correction,False,,, OptionButtonExact,OptionButton,0,True,100000000501_Options,True,Exact p-value,False,,, OptionButtonMVRemove,OptionButton,0,True,200000000100_Missing data,True,Remove the observations,False,,, OptionButtonMVRefuse,OptionButton,-1,True,200000000000_Missing data,True,Do not accept missing data,False,,, OptionButtonMVEstimate,OptionButton,0,True,200000000200_Missing data,True,Estimate missing data,False,,, OptionButtonMeanMode,OptionButton,-1,True,200000000300_Missing data,True,Mean,False,,, OptionButtonMVIgnore,OptionButton,0,True,200000000400_Missing data,True,Ignore missing data,False,,, TextBoxMaxTime,TextBox,180,True,100000010601_Options,True,Maximum time (s):,False,,,  865276  Coffee shops n=39*SEP*Summary statistics*SEP*$M$75 Coffee shops n=39*SEP*Mood test*SEP*$M$83 Coffee shops n=39*SEP*95% confidence interval on the p-value*SEP*$M$98 Coffee shops n=39*SEP*Multiple pairwise comparisons*SEP*$M$101 Coffee shops n=39*SEP*Mood test (01/06/2021\|01/10/2021)*SEP*$M$103 Coffee shops n=39*SEP*95% confidence interval on the p-value*SEP*$M$118 Coffee shops n=39*SEP*Mood test (01/06/2021\|01/12/2021)*SEP*$M$121 Coffee shops n=39*SEP*95% confidence interval on the p-value*SEP*$M$136 Coffee shops n=39*SEP*Mood test (01/10/2021\|01/12/2021)*SEP*$M$139 Coffee shops n=39*SEP*95% confidence interval on the p-value*SEP*$M$154  Coffee shops n=39*SEP*Summary statistics*SEP*$M$75 Coffee shops n=39*SEP*Mood test*SEP*$M$83 Coffee shops n=39*SEP*95% confidence interval on the p-value*SEP*$M$98 Coffee shops n=39*SEP*Multiple pairwise comparisons*SEP*$M$101 Coffee shops n=39*SEP*Mood test (01/06/2021\|01/10/2021)*SEP*$M$103 Coffee shops n=39*SEP*95% confidence interval on the p-value*SEP*$M$118 Coffee shops n=39*SEP*Mood test (01/06/2021\|01/12/2021)*SEP*$M$121 Coffee shops n=39*SEP*95% confidence interval on the p-value*SEP*$M$136 Coffee shops n=39*SEP*Mood test (01/10/2021\|01/12/2021)*SEP*$M$139 Coffee shops n=39*SEP*95% confidence interval on the p-value*SEP*$M$154 | 19.066 | 18.733 | 4.443 |  |  |  |  |  |  |
| Variance (n-1) | 19.568 | 19.226 | 4.560 |  |  |  |  |  |  |
| Standard deviation (n) | 4.367 | 4.297 | 0.521 |  |  |  |  |  |  |
| Standard deviation (n-1) | 4.424 | 4.353 | 0.527 |  |  |  |  |  |  |
| Median | 15.000 | 15.350 | 1.276 |  |  |  |  |  |  |
| Median absolute deviation | 0.000 | 1.030 | 2.023 |  |  |  |  |  |  |
|  |  |  |  |  |  |  |  |  |  |
| **Results of the resampling (OBS 2):** | | |  |  |  |  |  |  |  |
|  |  |  |  |  |  |  |  |  |  |
| Parameters | Estimator | Estimator (Bootstrap) | Standard deviation (Bootstrap) |  |  |  |  |  |  |
| Mean | 24.231 | 24.208 | 1.263 |  |  |  |  |  |  |
| Variance (n) | 57.101 | 55.729 | 15.203 |  |  |  |  |  |  |
| Variance (n-1) | 58.603 | 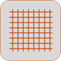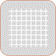57.196 | 15.603 |  |  |  |  |  |  |
| Standard deviation (n) | 7.556 | 7.396 | 1.017 |  |  |  |  |  |  |
| Standard deviation (n-1) | 7.655 | 7.492 | 1.030 |  |  |  |  |  |  |
| Median | 25.000 | 22.810 | 2.804 |  |  |  |  |  |  |
| Median absolute deviation | 5.000 | 5.005 | 0.475 |  |  |  |  |  |  |
|  |  |  |  |  |  |  |  |  |  |
| **Mood test:**  RunProcMOO Form168.txt RefEditT,RefEdit0,'Sheet1'!$I$1:$K$40,True,000000000100_General,True,,False,,40,3 RefEditGroups,RefEdit,,True,000000000300_General,True,Sample identifiers:,False,,, CheckBoxTrans,CheckBox,0,False,03,False,Trans,False,,, CheckBoxPairwise,CheckBox,-1,True,000000000301_General,True,Multiple pairwise comparisons,False,,, OptionButtonSample,OptionButton,-1,True,000000000600_General,True,One column per sample,False,,, OptionButtonVariable,OptionButton,0,True,000000000700_General,True,One column per variable,False,,, CheckBoxLabels,CheckBox,-1,True,000000000201_General,True,Column labels,False,,, OptionButton_W,OptionButton,0,True,000000020001_General,True,Workbook,False,,, OptionButton_R,OptionButton,-1,True,000000000001_General,True,Range,False,,, OptionButton_S,OptionButton,0,True,000000010001_General,True,Sheet,False,,, RefEdit_R,RefEdit0,'Sheet1'!$M$63,True,000000000101_General,True,Range:,False,,0,0 TextBox_Conf,TextBox,5,True,100000010001_Options,True,Significance level (%):,False,,, CheckBox_Desc,CheckBox,-1,True,300000000000_Outputs,True,Descriptive statistics,False,,, OptionButtonAsympt,OptionButton,0,True,100000000101_Options,True,Asymptotic p-value,False,,, OptionButtonMonte,OptionButton,-1,True,100000000201_Options,True,Monte Carlo method,False,,, TextBoxPermut,TextBox,10000,True,100000010301_Options,True,Number of simulations:,False,,, CheckBoxCorrect,CheckBox,-1,True,100000000401_Options,True,Continuity correction,False,,, OptionButtonExact,OptionButton,0,True,100000000501_Options,True,Exact p-value,False,,, OptionButtonMVRemove,OptionButton,0,True,200000000100_Missing data,True,Remove the observations,False,,, OptionButtonMVRefuse,OptionButton,-1,True,200000000000_Missing data,True,Do not accept missing data,False,,, OptionButtonMVEstimate,OptionButton,0,True,200000000200_Missing data,True,Estimate missing data,False,,, OptionButtonMeanMode,OptionButton,-1,True,200000000300_Missing data,True,Mean,False,,, OptionButtonMVIgnore,OptionButton,0,True,200000000400_Missing data,True,Ignore missing data,False,,, TextBoxMaxTime,TextBox,180,True,100000010601_Options,True,Maximum time (s):,False,,,  630210 |  |  |  |  |  |  |  |  |  |
|  |  |  |  |  |  |  |  |  |  |
| U | 9.750 |  |  |  |  |  |  |  |  |
| Critical value | 3.841 |  |  |  |  |  |  |  |  |
| DF | 1.000 |  |  |  |  |  |  |  |  |
| p-value | 0.002 |  |  |  |  |  |  |  |  |
| alpha | 0.05 |  |  |  |  |  |  |  |  |
| The p-value has been computed using 10000 Monte Carlo simulations. Time elapsed: 0s.  RunProcRSP Form118.txt CheckBoxTrans,CheckBox,0,False,03,False,Trans,False,,, ListBoxQuanti,ListBox,,True,200000000000_Outputs,True,,False,,, CheckBoxHist,CheckBox,0,True,300000000000_Charts,True,Histograms,False,,, OptionButtonHistBar,OptionButton,-1,True,300000000100_Charts,True,Bars,False,,, OptionButtonHistCont,OptionButton,0,True,300000010100_Charts,True,Continuous line,False,,, CheckBoxCum,CheckBox,0,True,300000000200_Charts,True,Cumulative histograms,False,,, OptionButtonHisBased,OptionButton,-1,True,300000000300_Charts,True,Based on the histogram,False,,, OptionButtonECDF,OptionButton,0,True,300000010300_Charts,True,Empirical cumulative distribution,False,,, CheckBoxRData,CheckBox,0,True,200000000008_Outputs,True,Resamples,False,,, TextBoxConfPer,TextBox,95,True,200000000003_Outputs,True,Confidence interval (%):,False,,, CheckBoxRStat,CheckBox,0,True,200000000007_Outputs,True,Resampled statistics,False,,, CheckBoxNormInt,CheckBox,0,True,200000000004_Outputs,True,Standard bootstrap interval,False,,, CheckBoxPercInt,CheckBox,0,True,200000000005_Outputs,True,Simple percentile interval,False,,, CheckBoxBiasInt,CheckBox,0,True,200000000006_Outputs,True,B.C. percentile interval,False,,, OptionButton_MVRemove,OptionButton,0,True,100000000100_Missing data,True,Remove the observations,False,,, OptionButton_MVEstimate,OptionButton,0,True,100000000300_Missing data,True,Estimate missing data,False,,, OptionButton_MeanMode,OptionButton,-1,True,100000000400_Missing data,True,Mean,False,,, OptionButtonAll,OptionButton,-1,True,100000000200_Missing data,True,For all samples,False,,, OptionButtonRestrict,OptionButton,0,True,100000010200_Missing data,True,For the corresponding sample,False,,, OptionButtonMVRefuse,OptionButton,-1,True,100000000000_Missing data,True,Do not accept missing data,False,,, OptionButton_W,OptionButton,0,True,000000000001_General,True,Workbook,False,,, OptionButton_R,OptionButton,-1,True,000000010001_General,True,Range,False,,, OptionButton_S,OptionButton,0,True,000000020001_General,True,Sheet,False,,, RefEdit_R,RefEdit0,'Sheet1'!$M$48,True,000000000101_General,True,Range:,False,,0,0 CheckBoxVarLabels,CheckBox,0,True,000000000201_General,True,Sample labels,False,,, CheckBox_W,CheckBox,0,True,000000000301_General,True,Weights,False,,, RefEdit_W,RefEdit,,True,000000000401_General,True,Weights:,False,,, TextBox_Resample,TextBox,1000,True,000000000000_General,True,Number of samples:,False,,, RefEdit_X,RefEdit0,'Sheet1'!$J$2:$J$40,True,000000000100_General,True,Quantitative data:,False,,39,1 ComboBoxMethod,ComboBox,0,True,000000010300_General,True,Method:,False,,, TextBox_obs,TextBox,5,True,000000000500_General,True,Sample size:,False,,, ComboBoxChartY,ComboBox,2,True,300000000101_Charts,True,Ordinate of the histograms:,False,,,  1 ListBox 118 ListBoxQuanti  1 22  Sum,0 Mean,-1 Variance (n),-1 Variance (n-1),-1 Standard deviation (n),-1 Standard deviation (n-1),-1 Median,-1 1st Quartile,0 3rd Quartile,0 Variation coefficient,0 Standard error of the mean,0 Mean absolute deviation,0 Median absolute deviation,-1 Geometric mean,0 Geometric standard deviation,0 Harmonic mean,0 1-Percentile,0 99-Percentile,0 2.5-Percentile,0 97.5-Percentile,0 5-Percentile,0 95-Percentile,0  578601 | | | | | | |  |  |  |
|  |  |  |  |  |  |  |  |  |  |
| Test interpretation: |  | 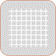 |  |  |  |  |  |  |  |
| H0: The medians of OBS 1 and OBS 2 are equal. | | |  |  |  |  |  |  |  |
| Ha: Medians of OBS 1 and OBS 2 are not equal  RunProcRSP Form118.txt CheckBoxTrans,CheckBox,0,False,03,False,Trans,False,,, ListBoxQuanti,ListBox,,True,200000000000_Outputs,True,,False,,, CheckBoxHist,CheckBox,0,True,300000000000_Charts,True,Histograms,False,,, OptionButtonHistBar,OptionButton,-1,True,300000000100_Charts,True,Bars,False,,, OptionButtonHistCont,OptionButton,0,True,300000010100_Charts,True,Continuous line,False,,, CheckBoxCum,CheckBox,0,True,300000000200_Charts,True,Cumulative histograms,False,,, OptionButtonHisBased,OptionButton,-1,True,300000000300_Charts,True,Based on the histogram,False,,, OptionButtonECDF,OptionButton,0,True,300000010300_Charts,True,Empirical cumulative distribution,False,,, CheckBoxRData,CheckBox,0,True,200000000008_Outputs,True,Resamples,False,,, TextBoxConfPer,TextBox,95,True,200000000003_Outputs,True,Confidence interval (%):,False,,, CheckBoxRStat,CheckBox,0,True,200000000007_Outputs,True,Resampled statistics,False,,, CheckBoxNormInt,CheckBox,0,True,200000000004_Outputs,True,Standard bootstrap interval,False,,, CheckBoxPercInt,CheckBox,0,True,200000000005_Outputs,True,Simple percentile interval,False,,, CheckBoxBiasInt,CheckBox,0,True,200000000006_Outputs,True,B.C. percentile interval,False,,, OptionButton_MVRemove,OptionButton,0,True,100000000100_Missing data,True,Remove the observations,False,,, OptionButton_MVEstimate,OptionButton,0,True,100000000300_Missing data,True,Estimate missing data,False,,, OptionButton_MeanMode,OptionButton,-1,True,100000000400_Missing data,True,Mean,False,,, OptionButtonAll,OptionButton,-1,True,100000000200_Missing data,True,For all samples,False,,, OptionButtonRestrict,OptionButton,0,True,100000010200_Missing data,True,For the corresponding sample,False,,, OptionButtonMVRefuse,OptionButton,-1,True,100000000000_Missing data,True,Do not accept missing data,False,,, OptionButton_W,OptionButton,0,True,000000000001_General,True,Workbook,False,,, OptionButton_R,OptionButton,-1,True,000000010001_General,True,Range,False,,, OptionButton_S,OptionButton,0,True,000000020001_General,True,Sheet,False,,, RefEdit_R,RefEdit0,'Sheet1'!$M$51,True,000000000101_General,True,Range:,False,,0,0 CheckBoxVarLabels,CheckBox,0,True,000000000201_General,True,Sample labels,False,,, CheckBox_W,CheckBox,0,True,000000000301_General,True,Weights,False,,, RefEdit_W,RefEdit,,True,000000000401_General,True,Weights:,False,,, TextBox_Resample,TextBox,1000,True,000000000000_General,True,Number of samples:,False,,, RefEdit_X,RefEdit0,'Sheet1'!$K$2:$K$40,True,000000000100_General,True,Quantitative data:,False,,39,1 ComboBoxMethod,ComboBox,0,True,000000010300_General,True,Method:,False,,, TextBox_obs,TextBox,5,True,000000000500_General,True,Sample size:,False,,, ComboBoxChartY,ComboBox,2,True,300000000101_Charts,True,Ordinate of the histograms:,False,,,  1 ListBox 118 ListBoxQuanti  1 22  Sum,0 Mean,-1 Variance (n),-1 Variance (n-1),-1 Standard deviation (n),-1 Standard deviation (n-1),-1 Median,-1 1st Quartile,0 3rd Quartile,0 Variation coefficient,0 Standard error of the mean,0 Mean absolute deviation,0 Median absolute deviation,-1 Geometric mean,0 Geometric standard deviation,0 Harmonic mean,0 1-Percentile,0 99-Percentile,0 2.5-Percentile,0 97.5-Percentile,0 5-Percentile,0 95-Percentile,0  362513  Coffee shops n=39*SEP*Summary statistics*SEP*$M$62 Coffee shops n=39*SEP*Results of the resampling (X1)*SEP*$M$68 | | |  |  |  |  |  |  |  |
| As the computed p-value is lower than the significance level alpha=0.05, one should reject the null hypothesis H0, and accept the alternative hypothesis Ha. | | | | |  |  |  |  |  |
|  |  |  |  |  |  |  |  |  |  |

| **Fast Foods** | **OBS 1** | **OBS 2** |
| --- | --- | --- |
| Bowl! | 25 | 20 |
| Burger Drive | 15 | 20 |
| Burger King Porto Antico | 20 | 30 |
| Burger King Via Mantovani | 15 | 30 |
| Chicken & Chicken | 15 | 30 |
| Chicken & Chicken Via Contubernio | 15 | 20 |
| Eat Italy | 30 | 25 |
| Fast Food Amico | 15 | 30 |
| Frigggitoria Carega | 20 | 30 |
| Gnam Gnam | 25 | 25 |
| Il Masetto | 20 | 25 |
| KFC Fiumara | 20 | 25 |
| King Kebab | 25 | 60 |
| La focacceria e dintorni | 15 | 45 |
| La Moucca | 20 | 30 |
| La Piadineria | 15 | 20 |
| Lo Stramburger | 20 | 25 |
| Mc Donald Fiumara | 15 | 26 |
| Mc Donald Via Fiume | 15 | 20 |
| Mc Donald Via di Sottoripa | 10 | 20 |
| Mc Donald Via XX Settembre | 15 | 20 |
| Old Wild West | 10 | 20 |
| Poke and Bowl | 20 | 30 |
| Rooster Via Fiasella | 20 | 30 |
| Strakkino | 25 | 30 |
| Strike | 30 | 30 |

| **Descriptives** | **OBS 1** | **OBS 2** |  |  |  |  |  |  |  |
| --- | --- | --- | --- | --- | --- | --- | --- | --- | --- |
| Nbr. of observations | 26 | 26 |  |  |  |  |  |  |  |
| Nbr. of missing values | 0 | 0 |  |  |  |  |  |  |  |
| Obs. without missing data | 26 | 26 |  |  |  |  |  |  |  |
| Minimum | 10.000 | 20.000 |  |  |  |  |  |  |  |
| Maximum | 30.000 | 60.000 |  |  |  |  |  |  |  |
| Freq. of minimum | 2 | 8 |  |  |  |  |  |  |  |
| Freq. of maximum | 2 | 1 |  |  |  |  |  |  |  |
| Median | 20.000 | 25.500 |  |  |  |  |  |  |  |
| Mean | 18.846 | 27.538 |  |  |  |  |  |  |  |
| Variance (n-1) | 28.615 | 76.338 |  |  |  |  |  |  |  |
| Standard deviation (n-1) | 5.349 | 8.737 |  |  |  |  |  |  |  |
| Median absolute deviation | 5.000 | 4.500 |  |  |  |  |  |  |  |
|  |  |  |  |  |  |  |  |  |  |
| **Shapiro-Wilk test (OBS 1):** |  |  |  |  |  |  |  |  |  |
|  |  |  |  |  |  |  |  |  |  |
| W | 0.900 |  |  |  |  |  |  |  |  |
| p-value (Two-tailed) | **0.015** |  |  |  |  |  |  |  |  |
| alpha | 0.05 |  |  |  |  |  |  |  |  |
| Fast foods n=26*SEP*Summary statistics*SEP*$M$39 Fast foods n=26*SEP*Results of the resampling (X1)*SEP*$M$45 |  |  |  |  |  |  |  |  |  |
| Test interpretation: |  |  |  |  |  |  |  |  |  |
| H0: The variable from which the sample was extracted follows a Normal distribution. | | | | | |  |  |  |  |
| Ha: The variable from which the sample was extracted does not follow a Normal distribution. | | | | | | |  |  |  |
| As the computed p-value is lower than the significance level alpha=0.05, one should  reject the null hypothesis H0, and accept the alternative hypothesis Ha. | | | | | | | | |  |
|  |  |  |  |  |  |  |  |  |  |

| **Shapiro-Wilk test (OBS 2):** | |  |  |  |  |  |  |  |  |
| --- | --- | --- | --- | --- | --- | --- | --- | --- | --- |
|  |  |  |  |  |  |  |  |  |  |
| W | 0.712 |  |  |  |  |  |  |  |  |
| p-value (Two-tailed) | **<0.0001** |  |  |  |  |  |  |  |  |
| alpha | 0.05 |  |  |  |  |  |  |  |  |
|  |  |  |  |  |  |  |  |  |  |
| Test interpretation: |  |  |  |  |  |  |  |  |  |
| H0: The variable from which the sample was extracted follows a Normal distribution. | | | | | | |  |  |  |
| Ha: The variable from which the sample was extracted does not follow a Normal distribution. | | | | | | | |  |  |
| As the computed p-value is lower than the significance level alpha=0.05,  one should reject the null hypothesis H0, and accept the alternative hypothesis Ha. | | | | | | | | |  |
|  |  |  |  |  |  |  |  |  |  |

| **Results of the resampling (OBS 1):**  RunProcRSP Form118.txt CheckBoxTrans,CheckBox,0,False,03,False,Trans,False,,, ListBoxQuanti,ListBox,,True,200000000000_Outputs,True,,False,,, CheckBoxHist,CheckBox,0,True,300000000000_Charts,True,Histograms,False,,, OptionButtonHistBar,OptionButton,-1,True,300000000100_Charts,True,Bars,False,,, OptionButtonHistCont,OptionButton,0,True,300000010100_Charts,True,Continuous line,False,,, CheckBoxCum,CheckBox,0,True,300000000200_Charts,True,Cumulative histograms,False,,, OptionButtonHisBased,OptionButton,-1,True,300000000300_Charts,True,Based on the histogram,False,,, OptionButtonECDF,OptionButton,0,True,300000010300_Charts,True,Empirical cumulative distribution,False,,, CheckBoxRData,CheckBox,0,True,200000000008_Outputs,True,Resamples,False,,, TextBoxConfPer,TextBox,95,True,200000000003_Outputs,True,Confidence interval (%):,False,,, CheckBoxRStat,CheckBox,0,True,200000000007_Outputs,True,Resampled statistics,False,,, CheckBoxNormInt,CheckBox,0,True,200000000004_Outputs,True,Standard bootstrap interval,False,,, CheckBoxPercInt,CheckBox,0,True,200000000005_Outputs,True,Simple percentile interval,False,,, CheckBoxBiasInt,CheckBox,0,True,200000000006_Outputs,True,B.C. percentile interval,False,,, OptionButton_MVRemove,OptionButton,0,True,100000000100_Missing data,True,Remove the observations,False,,, OptionButton_MVEstimate,OptionButton,0,True,100000000300_Missing data,True,Estimate missing data,False,,, OptionButton_MeanMode,OptionButton,-1,True,100000000400_Missing data,True,Mean,False,,, OptionButtonAll,OptionButton,-1,True,100000000200_Missing data,True,For all samples,False,,, OptionButtonRestrict,OptionButton,0,True,100000010200_Missing data,True,For the corresponding sample,False,,, OptionButtonMVRefuse,OptionButton,-1,True,100000000000_Missing data,True,Do not accept missing data,False,,, OptionButton_W,OptionButton,0,True,000000000001_General,True,Workbook,False,,, OptionButton_R,OptionButton,-1,True,000000010001_General,True,Range,False,,, OptionButton_S,OptionButton,0,True,000000020001_General,True,Sheet,False,,, RefEdit_R,RefEdit0,'Sheet2'!$M$28,True,000000000101_General,True,Range:,False,,0,0 CheckBoxVarLabels,CheckBox,0,True,000000000201_General,True,Sample labels,False,,, CheckBox_W,CheckBox,0,True,000000000301_General,True,Weights,False,,, RefEdit_W,RefEdit,,True,000000000401_General,True,Weights:,False,,, TextBox_Resample,TextBox,1000,True,000000000000_General,True,Number of samples:,False,,, RefEdit_X,RefEdit0,'Sheet2'!$I$2:$I$27,True,000000000100_General,True,Quantitative data:,False,,26,1 ComboBoxMethod,ComboBox,0,True,000000010300_General,True,Method:,False,,, TextBox_obs,TextBox,5,True,000000000500_General,True,Sample size:,False,,, ComboBoxChartY,ComboBox,2,True,300000000101_Charts,True,Ordinate of the histograms:,False,,,  1 ListBox 118 ListBoxQuanti  1 22  Sum,0 Mean,-1 Variance (n),-1 Variance (n-1),-1 Standard deviation (n),-1 Standard deviation (n-1),-1 Median,-1 1st Quartile,0 3rd Quartile,0 Variation coefficient,0 Standard error of the mean,0 Mean absolute deviation,0 Median absolute deviation,-1 Geometric mean,0 Geometric standard deviation,0 Harmonic mean,0 1-Percentile,0 99-Percentile,0 2.5-Percentile,0 97.5-Percentile,0 5-Percentile,0 95-Percentile,0  661160  Fast foods n=26*SEP*Summary statistics*SEP*$M$39 Fast foods n=26*SEP*Results of the resampling (X1)*SEP*$M$45 | | | | |  | | | |  | | | |  |  |  |  |
| --- | --- | --- | --- | --- | --- | --- | --- | --- | --- | --- | --- | --- | --- | --- | --- | --- |
|  | |  | | |  | | | |  | | | |  |  |  |  |
| Parameters | | Estimator | | | Estimator (Bootstrap) | | | | Standard deviation (Bootstrap) | | | |  |  |  |  |
| Mean | | 18.846 | | | 18.945 | | | | 1.048 | | | |  |  |  |  |
| Variance (n) | | 27.515 | | | 26.777 | | | | 6.923 | | | |  |  |  |  |
| Variance (n-1) | | 28.615 | | | 27.848 | | | | 7.200 | | | |  |  |  |  |
| Standard deviation (n) | | 5.245 | | | 5.131 | | | | 0.674 | | | |  |  |  |  |
| Standard deviation (n-1) | | 5.349 | | | 5.232 | | | | 0.687 | | | |  |  |  |  |
| Median | | 20.000 | | | 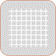18.363 | | | | 2.163 | | | |  |  |  |  |
| Median absolute deviation | | 5.000 | | | 4.048 | | | | 1.593 | | | |  |  |  |  |
|  | |  | | |  | | | |  | | | |  |  |  |  |
| **Results of the resampling (OBS 2):** | | | | | | | |  | | | |  | |  |  |  |
|  |  | | |  | | | |  | | | |  | |  |  |  |
| Parameters | Estimator | | | Estimator (Bootstrap) | | | | Standard deviation (Bootstrap) | | | |  | |  |  |  |
| Mean | 27.538 | | | 27.484 | | | | 1.657 | | | |  | |  |  |  |
| Variance (n) | 73.402 | | | 69.705 | | | | 38.007 | | | |  | |  |  |  |
| Variance (n-1) | 76.338 | | | 72.493 | | | | 39.527 | | | |  | |  |  |  |
| Standard deviation (n) | 8.568 | | | 8.015 | | | | 2.340 | | | |  | |  |  |  |
| Standard deviation (n-1) | 8.737 | | | 8.173 | | | | 2.387 | | | |  | |  |  |  |
| Median | 25.500 | | | 26.711 | | | | 2.321 | | | |  | |  |  |  |
| Median absolute deviation | 4.500 | | | 3.991 | | | | 1.693 | | | |  | |  |  |  |
|  |  | | |  | | | |  | | | |  | |  |  |  |
| **Mood test:**  RunProcRSP Form118.txt CheckBoxTrans,CheckBox,0,False,03,False,Trans,False,,, ListBoxQuanti,ListBox,,True,200000000000_Outputs,True,,False,,, CheckBoxHist,CheckBox,0,True,300000000000_Charts,True,Histograms,False,,, OptionButtonHistBar,OptionButton,-1,True,300000000100_Charts,True,Bars,False,,, OptionButtonHistCont,OptionButton,0,True,300000010100_Charts,True,Continuous line,False,,, CheckBoxCum,CheckBox,0,True,300000000200_Charts,True,Cumulative histograms,False,,, OptionButtonHisBased,OptionButton,-1,True,300000000300_Charts,True,Based on the histogram,False,,, OptionButtonECDF,OptionButton,0,True,300000010300_Charts,True,Empirical cumulative distribution,False,,, CheckBoxRData,CheckBox,0,True,200000000008_Outputs,True,Resamples,False,,, TextBoxConfPer,TextBox,95,True,200000000003_Outputs,True,Confidence interval (%):,False,,, CheckBoxRStat,CheckBox,0,True,200000000007_Outputs,True,Resampled statistics,False,,, CheckBoxNormInt,CheckBox,0,True,200000000004_Outputs,True,Standard bootstrap interval,False,,, CheckBoxPercInt,CheckBox,0,True,200000000005_Outputs,True,Simple percentile interval,False,,, CheckBoxBiasInt,CheckBox,0,True,200000000006_Outputs,True,B.C. percentile interval,False,,, OptionButton_MVRemove,OptionButton,0,True,100000000100_Missing data,True,Remove the observations,False,,, OptionButton_MVEstimate,OptionButton,0,True,100000000300_Missing data,True,Estimate missing data,False,,, OptionButton_MeanMode,OptionButton,-1,True,100000000400_Missing data,True,Mean,False,,, OptionButtonAll,OptionButton,-1,True,100000000200_Missing data,True,For all samples,False,,, OptionButtonRestrict,OptionButton,0,True,100000010200_Missing data,True,For the corresponding sample,False,,, OptionButtonMVRefuse,OptionButton,-1,True,100000000000_Missing data,True,Do not accept missing data,False,,, OptionButton_W,OptionButton,0,True,000000000001_General,True,Workbook,False,,, OptionButton_R,OptionButton,-1,True,000000010001_General,True,Range,False,,, OptionButton_S,OptionButton,0,True,000000020001_General,True,Sheet,False,,, RefEdit_R,RefEdit0,'Sheet2'!$M$56,True,000000000101_General,True,Range:,False,,0,0 CheckBoxVarLabels,CheckBox,0,True,000000000201_General,True,Sample labels,False,,, CheckBox_W,CheckBox,0,True,000000000301_General,True,Weights,False,,, RefEdit_W,RefEdit,,True,000000000401_General,True,Weights:,False,,, TextBox_Resample,TextBox,1000,True,000000000000_General,True,Number of samples:,False,,, RefEdit_X,RefEdit0,'Sheet2'!$J$2:$J$27,True,000000000100_General,True,Quantitative data:,False,,26,1 ComboBoxMethod,ComboBox,0,True,000000010300_General,True,Method:,False,,, TextBox_obs,TextBox,5,True,000000000500_General,True,Sample size:,False,,, ComboBoxChartY,ComboBox,2,True,300000000101_Charts,True,Ordinate of the histograms:,False,,,  1 ListBox 118 ListBoxQuanti  1 22  Sum,0 Mean,-1 Variance (n),-1 Variance (n-1),-1 Standard deviation (n),-1 Standard deviation (n-1),-1 Median,-1 1st Quartile,0 3rd Quartile,0 Variation coefficient,0 Standard error of the mean,0 Mean absolute deviation,0 Median absolute deviation,-1 Geometric mean,0 Geometric standard deviation,0 Harmonic mean,0 1-Percentile,0 99-Percentile,0 2.5-Percentile,0 97.5-Percentile,0 5-Percentile,0 95-Percentile,0  381560  Fast foods n=26*SEP*Summary statistics*SEP*$M$67 Fast foods n=26*SEP*Results of the resampling (X1)*SEP*$M$73 | | |  | | |  |  | | |  |  |  | |  |  |  |
|  | | |  | | |  |  | | |  |  |  | |  |  |  |
| U | | | 9.363 | | |  |  | | |  |  |  | |  |  |  |
| Critical value | | | 3.841 | | |  |  | | |  |  |  | |  |  |  |
| DF | | | 1.000 | | |  |  | | |  |  |  | |  |  |  |
| p-value | | | 0.001 | | |  |  | | |  |  |  | |  |  |  |
| alpha | | | 0.05 | | |  |  | | |  |  |  | |  |  |  |
| The p-value has been computed using 10000 Monte Carlo simulations. Time elapsed: 0s. | | | | | | | | | | | | | |  |  |  |
|  | | |  | | |  |  | | |  |  |  | |  |  |  |
| Test interpretation: | | |  | | |  |  | | |  |  |  | |  |  |  |
| H0: The medians of OBS 1 and OBS 2 are equal. | | | | | | |  | | |  |  |  | |  |  |  |
| Ha: Medians of OBS 1 and OBS 2 are not equal | | | | | |  |  | | |  |  |  | |  |  |  |
| As the computed p-value is lower than the significance level alpha=0.05, one should  RunProcRSP Form118.txt CheckBoxTrans,CheckBox,0,False,03,False,Trans,False,,, ListBoxQuanti,ListBox,,True,200000000000_Outputs,True,,False,,, CheckBoxHist,CheckBox,0,True,300000000000_Charts,True,Histograms,False,,, OptionButtonHistBar,OptionButton,-1,True,300000000100_Charts,True,Bars,False,,, OptionButtonHistCont,OptionButton,0,True,300000010100_Charts,True,Continuous line,False,,, CheckBoxCum,CheckBox,0,True,300000000200_Charts,True,Cumulative histograms,False,,, OptionButtonHisBased,OptionButton,-1,True,300000000300_Charts,True,Based on the histogram,False,,, OptionButtonECDF,OptionButton,0,True,300000010300_Charts,True,Empirical cumulative distribution,False,,, CheckBoxRData,CheckBox,0,True,200000000008_Outputs,True,Resamples,False,,, TextBoxConfPer,TextBox,95,True,200000000003_Outputs,True,Confidence interval (%):,False,,, CheckBoxRStat,CheckBox,0,True,200000000007_Outputs,True,Resampled statistics,False,,, CheckBoxNormInt,CheckBox,0,True,200000000004_Outputs,True,Standard bootstrap interval,False,,, CheckBoxPercInt,CheckBox,0,True,200000000005_Outputs,True,Simple percentile interval,False,,, CheckBoxBiasInt,CheckBox,0,True,200000000006_Outputs,True,B.C. percentile interval,False,,, OptionButton_MVRemove,OptionButton,0,True,100000000100_Missing data,True,Remove the observations,False,,, OptionButton_MVEstimate,OptionButton,0,True,100000000300_Missing data,True,Estimate missing data,False,,, OptionButton_MeanMode,OptionButton,-1,True,100000000400_Missing data,True,Mean,False,,, OptionButtonAll,OptionButton,-1,True,100000000200_Missing data,True,For all samples,False,,, OptionButtonRestrict,OptionButton,0,True,100000010200_Missing data,True,For the corresponding sample,False,,, OptionButtonMVRefuse,OptionButton,-1,True,100000000000_Missing data,True,Do not accept missing data,False,,, OptionButton_W,OptionButton,0,True,000000000001_General,True,Workbook,False,,, OptionButton_R,OptionButton,-1,True,000000010001_General,True,Range,False,,, OptionButton_S,OptionButton,0,True,000000020001_General,True,Sheet,False,,, RefEdit_R,RefEdit0,'Sheet2'!$M$84,True,000000000101_General,True,Range:,False,,0,0 CheckBoxVarLabels,CheckBox,0,True,000000000201_General,True,Sample labels,False,,, CheckBox_W,CheckBox,0,True,000000000301_General,True,Weights,False,,, RefEdit_W,RefEdit,,True,000000000401_General,True,Weights:,False,,, TextBox_Resample,TextBox,1000,True,000000000000_General,True,Number of samples:,False,,, RefEdit_X,RefEdit0,'Sheet2'!$K$2:$K$27,True,000000000100_General,True,Quantitative data:,False,,26,1 ComboBoxMethod,ComboBox,0,True,000000010300_General,True,Method:,False,,, TextBox_obs,TextBox,5,True,000000000500_General,True,Sample size:,False,,, ComboBoxChartY,ComboBox,2,True,300000000101_Charts,True,Ordinate of the histograms:,False,,,  1 ListBox 118 ListBoxQuanti  1 22  Sum,0 Mean,-1 Variance (n),-1 Variance (n-1),-1 Standard deviation (n),-1 Standard deviation (n-1),-1 Median,-1 1st Quartile,0 3rd Quartile,0 Variation coefficient,0 Standard error of the mean,0 Mean absolute deviation,0 Median absolute deviation,-1 Geometric mean,0 Geometric standard deviation,0 Harmonic mean,0 1-Percentile,0 99-Percentile,0 2.5-Percentile,0 97.5-Percentile,0 5-Percentile,0 95-Percentile,0  555637  Fast foods n=26*SEP*Summary statistics*SEP*$M$95 Fast foods n=26*SEP*Results of the resampling (X1)*SEP*$M$101  reject the null hypothesis H0, and accept the alternative hypothesis Ha. | | | | | | | | | | | | | | | |  |
|  |  |  |  |  |  |  |  |  |  |  |  |  |  |  |  |  |
|  | | |  | | |  |  | | |  |  |  | |  |  |  |

| **Pizzerie** | **OBS 1** | **OBS 2** |
| --- | --- | --- |
| Al solito posto | 20 | 60 |
| Al Vulcano | 15 | 45 |
| Antica Vaccheria | 15 | 60 |
| Arte Pizza | 20 | 30 |
| Brera Express | 30 | 30 |
| City | 20 | 60 |
| Da Franz & Co. | 15 | 60 |
| Da Silva | 15 | 60 |
| Dal Mister | 10 | 45 |
| Eataly | 30 | 30 |
| Eh..già | 20 | 60 |
| Exultate | 60 | 60 |
| Focone | 90 | 90 |
| Fratelli La Bufala | 15 | 30 |
| Fuorigrotta | 15 | 60 |
| Gomez | 20 | 40 |
| I Sassi | 45 | 45 |
| Il Baluardo | 60 | 60 |
| Il Mortaio | 15 | 45 |
| Lo Scugnizzo | 15 | 60 |
| Massarjia | 30 | 30 |
| MoroMare | 20 | 40 |
| MoroMare Nervi | 60 | 60 |
| New O Sole Mio | 15 | 45 |
| Osteria della Piazza | 45 | 45 |
| Pestello d'oro | 60 | 60 |
| Pizza Express | 15 | 60 |
| Pizzeria Stadio | 10 | 30 |
| Pluto 2 | 15 | 20 |
| Pulcinella | 15 | 30 |
| Punta Vagno | 15 | 40 |
| Sbrano | 10 | 45 |
| Sosta Obbligata | 20 | 50 |
| Strakkino | 25 | 40 |
| Totò e Peppino | 15 | 60 |
| Voglie di Pizza | 20 | 45 |

| **Descriptives** | **OBS 1** | **OBS 2** |  |  |  |  |  |  |  |
| --- | --- | --- | --- | --- | --- | --- | --- | --- | --- |
| Nbr. of observations | 36 | 36 |  |  |  |  |  |  |  |
| Nbr. of missing values | 0 | 0 |  |  |  |  |  |  |  |
| Obs. without missing data | 36 | 36 |  |  |  |  |  |  |  |
| Minimum | 10.000 | 20.000 |  |  |  |  |  |  |  |
| Maximum | 90.000 | 90.000 |  |  |  |  |  |  |  |
| Freq. of minimum | 3 | 1 |  |  |  |  |  |  |  |
| Freq. of maximum | 1 | 1 |  |  |  |  |  |  |  |
| Median | 20.000 | 45.000 |  |  |  |  |  |  |  |
| Mean | 25.972 | 48.056 |  |  |  |  |  |  |  |
| Variance (n-1) | 349.742 | 204.683 |  |  |  |  |  |  |  |
| Standard deviation (n-1) | 18.701 | 14.307 |  |  |  |  |  |  |  |
| Median absolute deviation | 5.000 | 15.000 |  |  |  |  |  |  |  |
|  |  |  |  |  |  |  |  |  |  |
| **Shapiro-Wilk test (OBS 1):** | |  |  |  |  |  |  |  |  |
|  |  |  |  |  |  |  |  |  |  |
| W | 0.718 |  |  |  |  |  |  |  |  |
| p-value (Two-tailed) | **<0.0001** |  |  |  |  |  |  |  |  |
| alpha | 0.05 |  |  |  |  |  |  |  |  |
|  |  |  |  |  |  |  |  |  |  |
| Test interpretation:  829246  Pizzerie n=55*SEP*Summary statistics*SEP*$M$69 Pizzerie n=55*SEP*Results of the resampling (X1)*SEP*$M$75  683259  Pizzerie n=55*SEP*Summary statistics*SEP*$M$125 Pizzerie n=55*SEP*Results of the resampling (X1)*SEP*$M$131  777618  462788  Pizzerie n=55*SEP*Summary statistics*SEP*$M$76 Pizzerie n=55*SEP*Mood test*SEP*$M$84 Pizzerie n=55*SEP*95% confidence interval on the p-value*SEP*$M$99 Pizzerie n=55*SEP*Multiple pairwise comparisons*SEP*$M$102 Pizzerie n=55*SEP*Mood test (01/06/2021\|01/10/2021)*SEP*$M$104 Pizzerie n=55*SEP*95% confidence interval on the p-value*SEP*$M$119 Pizzerie n=55*SEP*Mood test (01/06/2021\|01/12/2021)*SEP*$M$122 Pizzerie n=55*SEP*95% confidence interval on the p-value*SEP*$M$137 Pizzerie n=55*SEP*Mood test (01/10/2021\|01/12/2021)*SEP*$M$140 Pizzerie n=55*SEP*95% confidence interval on the p-value*SEP*$M$155  192417  Pizzerie n=55*SEP*Summary statistics*SEP*$M$76 Pizzerie n=55*SEP*Mood test*SEP*$M$84 Pizzerie n=55*SEP*95% confidence interval on the p-value*SEP*$M$99 Pizzerie n=55*SEP*Multiple pairwise comparisons*SEP*$M$102 Pizzerie n=55*SEP*Mood test (01/06/2021\|01/10/2021)*SEP*$M$104 Pizzerie n=55*SEP*95% confidence interval on the p-value*SEP*$M$119 Pizzerie n=55*SEP*Mood test (01/06/2021\|01/12/2021)*SEP*$M$122 Pizzerie n=55*SEP*95% confidence interval on the p-value*SEP*$M$137 Pizzerie n=55*SEP*Mood test (01/10/2021\|01/12/2021)*SEP*$M$140 Pizzerie n=55*SEP*95% confidence interval on the p-value*SEP*$M$155  966684  Pizzerie n=55*SEP*Summary statistics*SEP*$M$67 Pizzerie n=55*SEP*Mood test*SEP*$M$75 Pizzerie n=55*SEP*95% confidence interval on the p-value*SEP*$M$90 Pizzerie n=55*SEP*Multiple pairwise comparisons*SEP*$M$93 Pizzerie n=55*SEP*Mood test (01/06/2021\|01/10/2021)*SEP*$M$95 Pizzerie n=55*SEP*95% confidence interval on the p-value*SEP*$M$110 Pizzerie n=55*SEP*Mood test (01/06/2021\|01/12/2021)*SEP*$M$113 Pizzerie n=55*SEP*95% confidence interval on the p-value*SEP*$M$128 Pizzerie n=55*SEP*Mood test (01/10/2021\|01/12/2021)*SEP*$M$131 Pizzerie n=55*SEP*95% confidence interval on the p-value*SEP*$M$146  845859  Pizzerie n=45*SEP*Summary statistics*SEP*$M$58 Pizzerie n=45*SEP*Results of the resampling (X1)*SEP*$M$66 Pizzerie n=45*SEP*Results of the resampling (X2)*SEP*$M$78 Pizzerie n=45*SEP*Results of the resampling (X3)*SEP*$M$90  Pizzerie n=45*SEP*Summary statistics*SEP*$M$58 Pizzerie n=45*SEP*Results of the resampling (X1)*SEP*$M$66 Pizzerie n=45*SEP*Results of the resampling (X2)*SEP*$M$78 Pizzerie n=45*SEP*Results of the resampling (X3)*SEP*$M$90  840624  Pizzerie n=45*SEP*Summary statistics*SEP*$M$58 Pizzerie n=45*SEP*Results of the resampling (20)*SEP*$M$66 Pizzerie n=45*SEP*Results of the resampling (20)*SEP*$M$78 Pizzerie n=45*SEP*Results of the resampling (60)*SEP*$M$90  Pizzerie n=45*SEP*Summary statistics*SEP*$M$58 Pizzerie n=45*SEP*Results of the resampling (20)*SEP*$M$64 |  |  |  |  |  |  |  |  |  |
| H0: The variable from which the sample was extracted follows a Normal distribution. | | | | | | |  |  |  |
| Ha: The variable from which the sample was extracted does not follow a Normal distribution. | | | | | | | |  |  |
| As the computed p-value is lower than the significance level alpha=0.05,  one should reject the null hypothesis H0, and accept the alternative hypothesis Ha. | | | | | | | | |  |
|  |  |  |  |  |  |  |  |  |  |

| **Shapiro-Wilk test (OBS 2):** | |  |  |  |  |  |  |  |  |
| --- | --- | --- | --- | --- | --- | --- | --- | --- | --- |
|  |  |  |  |  |  |  |  |  |  |
| W | 0.889 |  |  |  |  |  |  |  |  |
| p-value (Two-tailed) | **0.002** |  |  |  |  |  |  |  |  |
| alpha | 0.05 |  |  |  |  |  |  |  |  |
|  |  |  |  |  |  |  |  |  |  |
| Test interpretation: |  |  |  |  |  |  |  |  |  |
| H0: The variable from which the sample was extracted follows a Normal distribution. | | | | | | |  |  |  |
| Ha: The variable from which the sample was extracted does not follow a Normal distribution. | | | | | | |  |  |  |
| As the computed p-value is lower than the significance level alpha=0.05,  one should reject the null hypothesis H0, and accept the alternative hypothesis Ha. | | | | | | | | |  |
|  |  |  |  |  |  |  |  |  |  |

| **Results of the resampling (OBS 1):**  RunProcRSP Form118.txt CheckBoxTrans,CheckBox,0,False,03,False,Trans,False,,, ListBoxQuanti,ListBox,,True,200000000000_Outputs,True,,False,,, CheckBoxHist,CheckBox,0,True,300000000000_Charts,True,Histograms,False,,, OptionButtonHistBar,OptionButton,-1,True,300000000100_Charts,True,Bars,False,,, OptionButtonHistCont,OptionButton,0,True,300000010100_Charts,True,Continuous line,False,,, CheckBoxCum,CheckBox,0,True,300000000200_Charts,True,Cumulative histograms,False,,, OptionButtonHisBased,OptionButton,-1,True,300000000300_Charts,True,Based on the histogram,False,,, OptionButtonECDF,OptionButton,0,True,300000010300_Charts,True,Empirical cumulative distribution,False,,, CheckBoxRData,CheckBox,0,True,200000000008_Outputs,True,Resamples,False,,, TextBoxConfPer,TextBox,95,True,200000000003_Outputs,True,Confidence interval (%):,False,,, CheckBoxRStat,CheckBox,0,True,200000000007_Outputs,True,Resampled statistics,False,,, CheckBoxNormInt,CheckBox,0,True,200000000004_Outputs,True,Standard bootstrap interval,False,,, CheckBoxPercInt,CheckBox,0,True,200000000005_Outputs,True,Simple percentile interval,False,,, CheckBoxBiasInt,CheckBox,0,True,200000000006_Outputs,True,B.C. percentile interval,False,,, OptionButton_MVRemove,OptionButton,0,True,100000000100_Missing data,True,Remove the observations,False,,, OptionButton_MVEstimate,OptionButton,0,True,100000000300_Missing data,True,Estimate missing data,False,,, OptionButton_MeanMode,OptionButton,-1,True,100000000400_Missing data,True,Mean,False,,, OptionButtonAll,OptionButton,-1,True,100000000200_Missing data,True,For all samples,False,,, OptionButtonRestrict,OptionButton,0,True,100000010200_Missing data,True,For the corresponding sample,False,,, OptionButtonMVRefuse,OptionButton,-1,True,100000000000_Missing data,True,Do not accept missing data,False,,, OptionButton_W,OptionButton,0,True,000000000001_General,True,Workbook,False,,, OptionButton_R,OptionButton,-1,True,000000010001_General,True,Range,False,,, OptionButton_S,OptionButton,0,True,000000020001_General,True,Sheet,False,,, RefEdit_R,RefEdit0,'Sheet3'!$M$86,True,000000000101_General,True,Range:,False,,0,0 CheckBoxVarLabels,CheckBox,0,True,000000000201_General,True,Sample labels,False,,, CheckBox_W,CheckBox,0,True,000000000301_General,True,Weights,False,,, RefEdit_W,RefEdit,,True,000000000401_General,True,Weights:,False,,, TextBox_Resample,TextBox,1000,True,000000000000_General,True,Number of samples:,False,,, RefEdit_X,RefEdit0,'Sheet3'!$J$2:$J$56,True,000000000100_General,True,Quantitative data:,False,,55,1 ComboBoxMethod,ComboBox,0,True,000000010300_General,True,Method:,False,,, TextBox_obs,TextBox,5,True,000000000500_General,True,Sample size:,False,,, ComboBoxChartY,ComboBox,2,True,300000000101_Charts,True,Ordinate of the histograms:,False,,,  1 ListBox 118 ListBoxQuanti  1 22  Sum,0 Mean,-1 Variance (n),-1 Variance (n-1),-1 Standard deviation (n),-1 Standard deviation (n-1),-1 Median,-1 1st Quartile,0 3rd Quartile,0 Variation coefficient,0 Standard error of the mean,0 Mean absolute deviation,0 Median absolute deviation,-1 Geometric mean,0 Geometric standard deviation,0 Harmonic mean,0 1-Percentile,0 99-Percentile,0 2.5-Percentile,0 97.5-Percentile,0 5-Percentile,0 95-Percentile,0  788449  Pizzerie n=55*SEP*Summary statistics*SEP*$M$97 Pizzerie n=55*SEP*Results of the resampling (X1)*SEP*$M$103  777618 | | | | | | | | |  | | | |  | |  | | |  |  |  |
| --- | --- | --- | --- | --- | --- | --- | --- | --- | --- | --- | --- | --- | --- | --- | --- | --- | --- | --- | --- | --- |
|  | |  | | |  | | | |  | | | |  | |  | | |  |  |  |
| Parameters | | Estimator | | | Estimator (Bootstrap) | | | | Standard deviation (Bootstrap) | | | |  | |  | | |  |  |  |
| Mean | | 25.972 | | | 25.883 | | | | 3.013 | | | |  | |  | | |  |  |  |
| Variance (n) | | 340.027 | | | 327.429 | | | | 113.563 | | | |  | |  | | |  |  |  |
| Variance (n-1) | | 349.742 | | | 336.784 | | | | 116.807 | | | |  | |  | | |  |  |  |
| Standard deviation (n)  RunProcRSP Form118.txt CheckBoxTrans,CheckBox,0,False,03,False,Trans,False,,, ListBoxQuanti,ListBox,,True,200000000000_Outputs,True,,False,,, CheckBoxHist,CheckBox,0,True,300000000000_Charts,True,Histograms,False,,, OptionButtonHistBar,OptionButton,-1,True,300000000100_Charts,True,Bars,False,,, OptionButtonHistCont,OptionButton,0,True,300000010100_Charts,True,Continuous line,False,,, CheckBoxCum,CheckBox,0,True,300000000200_Charts,True,Cumulative histograms,False,,, OptionButtonHisBased,OptionButton,-1,True,300000000300_Charts,True,Based on the histogram,False,,, OptionButtonECDF,OptionButton,0,True,300000010300_Charts,True,Empirical cumulative distribution,False,,, CheckBoxRData,CheckBox,0,True,200000000008_Outputs,True,Resamples,False,,, TextBoxConfPer,TextBox,95,True,200000000003_Outputs,True,Confidence interval (%):,False,,, CheckBoxRStat,CheckBox,0,True,200000000007_Outputs,True,Resampled statistics,False,,, CheckBoxNormInt,CheckBox,0,True,200000000004_Outputs,True,Standard bootstrap interval,False,,, CheckBoxPercInt,CheckBox,0,True,200000000005_Outputs,True,Simple percentile interval,False,,, CheckBoxBiasInt,CheckBox,0,True,200000000006_Outputs,True,B.C. percentile interval,False,,, OptionButton_MVRemove,OptionButton,0,True,100000000100_Missing data,True,Remove the observations,False,,, OptionButton_MVEstimate,OptionButton,0,True,100000000300_Missing data,True,Estimate missing data,False,,, OptionButton_MeanMode,OptionButton,-1,True,100000000400_Missing data,True,Mean,False,,, OptionButtonAll,OptionButton,-1,True,100000000200_Missing data,True,For all samples,False,,, OptionButtonRestrict,OptionButton,0,True,100000010200_Missing data,True,For the corresponding sample,False,,, OptionButtonMVRefuse,OptionButton,-1,True,100000000000_Missing data,True,Do not accept missing data,False,,, OptionButton_W,OptionButton,0,True,000000000001_General,True,Workbook,False,,, OptionButton_R,OptionButton,-1,True,000000010001_General,True,Range,False,,, OptionButton_S,OptionButton,0,True,000000020001_General,True,Sheet,False,,, RefEdit_R,RefEdit0,'Sheet3'!$M$58,True,000000000101_General,True,Range:,False,,1,1 CheckBoxVarLabels,CheckBox,0,True,000000000201_General,True,Sample labels,False,,, CheckBox_W,CheckBox,0,True,000000000301_General,True,Weights,False,,, RefEdit_W,RefEdit,,True,000000000401_General,True,Weights:,False,,, TextBox_Resample,TextBox,1000,True,000000000000_General,True,Number of samples:,False,,, RefEdit_X,RefEdit0,'Sheet3'!$I$2:$I$56,True,000000000100_General,True,Quantitative data:,False,,26,1 ComboBoxMethod,ComboBox,0,True,000000010300_General,True,Method:,False,,, TextBox_obs,TextBox,5,True,000000000500_General,True,Sample size:,False,,, ComboBoxChartY,ComboBox,2,True,300000000101_Charts,True,Ordinate of the histograms:,False,,,  1 ListBox 118 ListBoxQuanti  1 22  Sum,0 Mean,-1 Variance (n),-1 Variance (n-1),-1 Standard deviation (n),-1 Standard deviation (n-1),-1 Median,-1 1st Quartile,0 3rd Quartile,0 Variation coefficient,0 Standard error of the mean,0 Mean absolute deviation,0 Median absolute deviation,-1 Geometric mean,0 Geometric standard deviation,0 Harmonic mean,0 1-Percentile,0 99-Percentile,0 2.5-Percentile,0 97.5-Percentile,0 5-Percentile,0 95-Percentile,0  RunProcRSP Form118.txt CheckBoxTrans,CheckBox,0,False,03,False,Trans,False,,, ListBoxQuanti,ListBox,,True,200000000000_Outputs,True,,False,,, CheckBoxHist,CheckBox,0,True,300000000000_Charts,True,Histograms,False,,, OptionButtonHistBar,OptionButton,-1,True,300000000100_Charts,True,Bars,False,,, OptionButtonHistCont,OptionButton,0,True,300000010100_Charts,True,Continuous line,False,,, CheckBoxCum,CheckBox,0,True,300000000200_Charts,True,Cumulative histograms,False,,, OptionButtonHisBased,OptionButton,-1,True,300000000300_Charts,True,Based on the histogram,False,,, OptionButtonECDF,OptionButton,0,True,300000010300_Charts,True,Empirical cumulative distribution,False,,, CheckBoxRData,CheckBox,0,True,200000000008_Outputs,True,Resamples,False,,, TextBoxConfPer,TextBox,95,True,200000000003_Outputs,True,Confidence interval (%):,False,,, CheckBoxRStat,CheckBox,0,True,200000000007_Outputs,True,Resampled statistics,False,,, CheckBoxNormInt,CheckBox,0,True,200000000004_Outputs,True,Standard bootstrap interval,False,,, CheckBoxPercInt,CheckBox,0,True,200000000005_Outputs,True,Simple percentile interval,False,,, CheckBoxBiasInt,CheckBox,0,True,200000000006_Outputs,True,B.C. percentile interval,False,,, OptionButton_MVRemove,OptionButton,0,True,100000000100_Missing data,True,Remove the observations,False,,, OptionButton_MVEstimate,OptionButton,0,True,100000000300_Missing data,True,Estimate missing data,False,,, OptionButton_MeanMode,OptionButton,-1,True,100000000400_Missing data,True,Mean,False,,, OptionButtonAll,OptionButton,-1,True,100000000200_Missing data,True,For all samples,False,,, OptionButtonRestrict,OptionButton,0,True,100000010200_Missing data,True,For the corresponding sample,False,,, OptionButtonMVRefuse,OptionButton,-1,True,100000000000_Missing data,True,Do not accept missing data,False,,, OptionButton_W,OptionButton,0,True,000000000001_General,True,Workbook,False,,, OptionButton_R,OptionButton,-1,True,000000010001_General,True,Range,False,,, OptionButton_S,OptionButton,0,True,000000020001_General,True,Sheet,False,,, RefEdit_R,RefEdit0,'Sheet3'!$M$114,True,000000000101_General,True,Range:,False,,0,0 CheckBoxVarLabels,CheckBox,0,True,000000000201_General,True,Sample labels,False,,, CheckBox_W,CheckBox,0,True,000000000301_General,True,Weights,False,,, RefEdit_W,RefEdit,,True,000000000401_General,True,Weights:,False,,, TextBox_Resample,TextBox,1000,True,000000000000_General,True,Number of samples:,False,,, RefEdit_X,RefEdit0,'Sheet3'!$K$2:$K$56,True,000000000100_General,True,Quantitative data:,False,,55,1 ComboBoxMethod,ComboBox,0,True,000000010300_General,True,Method:,False,,, TextBox_obs,TextBox,5,True,000000000500_General,True,Sample size:,False,,, ComboBoxChartY,ComboBox,2,True,300000000101_Charts,True,Ordinate of the histograms:,False,,,  1 ListBox 118 ListBoxQuanti  1 22  Sum,0 Mean,-1 Variance (n),-1 Variance (n-1),-1 Standard deviation (n),-1 Standard deviation (n-1),-1 Median,-1 1st Quartile,0 3rd Quartile,0 Variation coefficient,0 Standard error of the mean,0 Mean absolute deviation,0 Median absolute deviation,-1 Geometric mean,0 Geometric standard deviation,0 Harmonic mean,0 1-Percentile,0 99-Percentile,0 2.5-Percentile,0 97.5-Percentile,0 5-Percentile,0 95-Percentile,0  RunProcMOO Form168.txt RefEditT,RefEdit0,'Sheet3'!$I$1:$K$56,True,000000000100_General,True,,False,,27,3 RefEditGroups,RefEdit,,True,000000000300_General,True,Sample identifiers:,False,,, CheckBoxTrans,CheckBox,0,False,03,False,Trans,False,,, CheckBoxPairwise,CheckBox,-1,True,000000000301_General,True,Multiple pairwise comparisons,False,,, OptionButtonSample,OptionButton,-1,True,000000000600_General,True,One column per sample,False,,, OptionButtonVariable,OptionButton,0,True,000000000700_General,True,One column per variable,False,,, CheckBoxLabels,CheckBox,-1,True,000000000201_General,True,Column labels,False,,, OptionButton_W,OptionButton,0,True,000000020001_General,True,Workbook,False,,, OptionButton_R,OptionButton,-1,True,000000000001_General,True,Range,False,,, OptionButton_S,OptionButton,0,True,000000010001_General,True,Sheet,False,,, RefEdit_R,RefEdit0,'Sheet3'!$M$64,True,000000000101_General,True,Range:,False,,1,1 TextBox_Conf,TextBox,5,True,100000010001_Options,True,Significance level (%):,False,,, CheckBox_Desc,CheckBox,-1,True,300000000000_Outputs,True,Descriptive statistics,False,,, OptionButtonAsympt,OptionButton,0,True,100000000101_Options,True,Asymptotic p-value,False,,, OptionButtonMonte,OptionButton,-1,True,100000000201_Options,True,Monte Carlo method,False,,, TextBoxPermut,TextBox,10000,True,100000010301_Options,True,Number of simulations:,False,,, CheckBoxCorrect,CheckBox,-1,True,100000000401_Options,True,Continuity correction,False,,, OptionButtonExact,OptionButton,0,True,100000000501_Options,True,Exact p-value,False,,, OptionButtonMVRemove,OptionButton,0,True,200000000100_Missing data,True,Remove the observations,False,,, OptionButtonMVRefuse,OptionButton,-1,True,200000000000_Missing data,True,Do not accept missing data,False,,, OptionButtonMVEstimate,OptionButton,0,True,200000000200_Missing data,True,Estimate missing data,False,,, OptionButtonMeanMode,OptionButton,-1,True,200000000300_Missing data,True,Mean,False,,, OptionButtonMVIgnore,OptionButton,0,True,200000000400_Missing data,True,Ignore missing data,False,,, TextBoxMaxTime,TextBox,180,True,100000010601_Options,True,Maximum time (s):,False,,,  RunProcMOO Form168.txt RefEditT,RefEdit0,'Sheet3'!$I$1:$K$56,True,000000000100_General,True,,False,,56,3 RefEditGroups,RefEdit,,True,000000000300_General,True,Sample identifiers:,False,,, CheckBoxTrans,CheckBox,0,False,03,False,Trans,False,,, CheckBoxPairwise,CheckBox,-1,True,000000000301_General,True,Multiple pairwise comparisons,False,,, OptionButtonSample,OptionButton,-1,True,000000000600_General,True,One column per sample,False,,, OptionButtonVariable,OptionButton,0,True,000000000700_General,True,One column per variable,False,,, CheckBoxLabels,CheckBox,-1,True,000000000201_General,True,Column labels,False,,, OptionButton_W,OptionButton,0,True,000000020001_General,True,Workbook,False,,, OptionButton_R,OptionButton,-1,True,000000000001_General,True,Range,False,,, OptionButton_S,OptionButton,0,True,000000010001_General,True,Sheet,False,,, RefEdit_R,RefEdit0,'Sheet3'!$M$64,True,000000000101_General,True,Range:,False,,0,0 TextBox_Conf,TextBox,5,True,100000010001_Options,True,Significance level (%):,False,,, CheckBox_Desc,CheckBox,-1,True,300000000000_Outputs,True,Descriptive statistics,False,,, OptionButtonAsympt,OptionButton,0,True,100000000101_Options,True,Asymptotic p-value,False,,, OptionButtonMonte,OptionButton,-1,True,100000000201_Options,True,Monte Carlo method,False,,, TextBoxPermut,TextBox,10000,True,100000010301_Options,True,Number of simulations:,False,,, CheckBoxCorrect,CheckBox,-1,True,100000000401_Options,True,Continuity correction,False,,, OptionButtonExact,OptionButton,0,True,100000000501_Options,True,Exact p-value,False,,, OptionButtonMVRemove,OptionButton,0,True,200000000100_Missing data,True,Remove the observations,False,,, OptionButtonMVRefuse,OptionButton,-1,True,200000000000_Missing data,True,Do not accept missing data,False,,, OptionButtonMVEstimate,OptionButton,0,True,200000000200_Missing data,True,Estimate missing data,False,,, OptionButtonMeanMode,OptionButton,-1,True,200000000300_Missing data,True,Mean,False,,, OptionButtonMVIgnore,OptionButton,0,True,200000000400_Missing data,True,Ignore missing data,False,,, TextBoxMaxTime,TextBox,180,True,100000010601_Options,True,Maximum time (s):,False,,,  RunProcMOO Form168.txt RefEditT,RefEdit0,'Sheet3'!$I$1:$K$46,True,000000000100_General,True,,False,,46,3 RefEditGroups,RefEdit,,True,000000000300_General,True,Sample identifiers:,False,,, CheckBoxTrans,CheckBox,0,False,03,False,Trans,False,,, CheckBoxPairwise,CheckBox,-1,True,000000000301_General,True,Multiple pairwise comparisons,False,,, OptionButtonSample,OptionButton,-1,True,000000000600_General,True,One column per sample,False,,, OptionButtonVariable,OptionButton,0,True,000000000700_General,True,One column per variable,False,,, CheckBoxLabels,CheckBox,-1,True,000000000201_General,True,Column labels,False,,, OptionButton_W,OptionButton,0,True,000000020001_General,True,Workbook,False,,, OptionButton_R,OptionButton,-1,True,000000000001_General,True,Range,False,,, OptionButton_S,OptionButton,0,True,000000010001_General,True,Sheet,False,,, RefEdit_R,RefEdit0,'Sheet3'!$M$55,True,000000000101_General,True,Range:,False,,1,1 TextBox_Conf,TextBox,5,True,100000010001_Options,True,Significance level (%):,False,,, CheckBox_Desc,CheckBox,-1,True,300000000000_Outputs,True,Descriptive statistics,False,,, OptionButtonAsympt,OptionButton,0,True,100000000101_Options,True,Asymptotic p-value,False,,, OptionButtonMonte,OptionButton,-1,True,100000000201_Options,True,Monte Carlo method,False,,, TextBoxPermut,TextBox,10000,True,100000010301_Options,True,Number of simulations:,False,,, CheckBoxCorrect,CheckBox,-1,True,100000000401_Options,True,Continuity correction,False,,, OptionButtonExact,OptionButton,0,True,100000000501_Options,True,Exact p-value,False,,, OptionButtonMVRemove,OptionButton,0,True,200000000100_Missing data,True,Remove the observations,False,,, OptionButtonMVRefuse,OptionButton,-1,True,200000000000_Missing data,True,Do not accept missing data,False,,, OptionButtonMVEstimate,OptionButton,0,True,200000000200_Missing data,True,Estimate missing data,False,,, OptionButtonMeanMode,OptionButton,-1,True,200000000300_Missing data,True,Mean,False,,, OptionButtonMVIgnore,OptionButton,0,True,200000000400_Missing data,True,Ignore missing data,False,,, TextBoxMaxTime,TextBox,180,True,100000010601_Options,True,Maximum time (s):,False,,,  RunProcRSP Form118.txt CheckBoxTrans,CheckBox,0,False,03,False,Trans,False,,, ListBoxQuanti,ListBox,,True,200000000000_Outputs,True,,False,,, CheckBoxHist,CheckBox,0,True,300000000000_Charts,True,Histograms,False,,, OptionButtonHistBar,OptionButton,-1,True,300000000100_Charts,True,Bars,False,,, OptionButtonHistCont,OptionButton,0,True,300000010100_Charts,True,Continuous line,False,,, CheckBoxCum,CheckBox,0,True,300000000200_Charts,True,Cumulative histograms,False,,, OptionButtonHisBased,OptionButton,-1,True,300000000300_Charts,True,Based on the histogram,False,,, OptionButtonECDF,OptionButton,0,True,300000010300_Charts,True,Empirical cumulative distribution,False,,, CheckBoxRData,CheckBox,0,True,200000000008_Outputs,True,Resamples,False,,, TextBoxConfPer,TextBox,95,True,200000000003_Outputs,True,Confidence interval (%):,False,,, CheckBoxRStat,CheckBox,0,True,200000000007_Outputs,True,Resampled statistics,False,,, CheckBoxNormInt,CheckBox,0,True,200000000004_Outputs,True,Standard bootstrap interval,False,,, CheckBoxPercInt,CheckBox,0,True,200000000005_Outputs,True,Simple percentile interval,False,,, CheckBoxBiasInt,CheckBox,0,True,200000000006_Outputs,True,B.C. percentile interval,False,,, OptionButton_MVRemove,OptionButton,0,True,100000000100_Missing data,True,Remove the observations,False,,, OptionButton_MVEstimate,OptionButton,0,True,100000000300_Missing data,True,Estimate missing data,False,,, OptionButton_MeanMode,OptionButton,-1,True,100000000400_Missing data,True,Mean,False,,, OptionButtonAll,OptionButton,-1,True,100000000200_Missing data,True,For all samples,False,,, OptionButtonRestrict,OptionButton,0,True,100000010200_Missing data,True,For the corresponding sample,False,,, OptionButtonMVRefuse,OptionButton,-1,True,100000000000_Missing data,True,Do not accept missing data,False,,, OptionButton_W,OptionButton,0,True,000000000001_General,True,Workbook,False,,, OptionButton_R,OptionButton,-1,True,000000010001_General,True,Range,False,,, OptionButton_S,OptionButton,0,True,000000020001_General,True,Sheet,False,,, RefEdit_R,RefEdit,'Sheet3'!$M$47,True,000000000101_General,True,Range:,False,,0,0 CheckBoxVarLabels,CheckBox,0,True,000000000201_General,True,Sample labels,False,,, CheckBox_W,CheckBox,0,True,000000000301_General,True,Weights,False,,, RefEdit_W,RefEdit,,True,000000000401_General,True,Weights:,False,,, TextBox_Resample,TextBox,1000,True,000000000000_General,True,Number of samples:,False,,, RefEdit_X,RefEdit0,'Sheet3'!$I$1:$K$46,True,000000000100_General,True,Quantitative data:,False,,46,3 ComboBoxMethod,ComboBox,0,True,000000010300_General,True,Method:,False,,, TextBox_obs,TextBox,5,True,000000000500_General,True,Sample size:,False,,, ComboBoxChartY,ComboBox,2,True,300000000101_Charts,True,Ordinate of the histograms:,False,,,  1 ListBox 118 ListBoxQuanti  1 22  Sum,0 Mean,-1 Variance (n),-1 Variance (n-1),-1 Standard deviation (n),-1 Standard deviation (n-1),-1 Median,-1 1st Quartile,0 3rd Quartile,0 Variation coefficient,0 Standard error of the mean,0 Mean absolute deviation,0 Median absolute deviation,-1 Geometric mean,0 Geometric standard deviation,0 Harmonic mean,0 1-Percentile,0 99-Percentile,0 2.5-Percentile,0 97.5-Percentile,0 5-Percentile,0 95-Percentile,0  124758  RunProcRSP Form118.txt CheckBoxTrans,CheckBox,0,False,03,False,Trans,False,,, ListBoxQuanti,ListBox,,True,200000000000_Outputs,True,,False,,, CheckBoxHist,CheckBox,0,True,300000000000_Charts,True,Histograms,False,,, OptionButtonHistBar,OptionButton,-1,True,300000000100_Charts,True,Bars,False,,, OptionButtonHistCont,OptionButton,0,True,300000010100_Charts,True,Continuous line,False,,, CheckBoxCum,CheckBox,0,True,300000000200_Charts,True,Cumulative histograms,False,,, OptionButtonHisBased,OptionButton,-1,True,300000000300_Charts,True,Based on the histogram,False,,, OptionButtonECDF,OptionButton,0,True,300000010300_Charts,True,Empirical cumulative distribution,False,,, CheckBoxRData,CheckBox,0,True,200000000008_Outputs,True,Resamples,False,,, TextBoxConfPer,TextBox,95,True,200000000003_Outputs,True,Confidence interval (%):,False,,, CheckBoxRStat,CheckBox,0,True,200000000007_Outputs,True,Resampled statistics,False,,, CheckBoxNormInt,CheckBox,0,True,200000000004_Outputs,True,Standard bootstrap interval,False,,, CheckBoxPercInt,CheckBox,0,True,200000000005_Outputs,True,Simple percentile interval,False,,, CheckBoxBiasInt,CheckBox,0,True,200000000006_Outputs,True,B.C. percentile interval,False,,, OptionButton_MVRemove,OptionButton,0,True,100000000100_Missing data,True,Remove the observations,False,,, OptionButton_MVEstimate,OptionButton,0,True,100000000300_Missing data,True,Estimate missing data,False,,, OptionButton_MeanMode,OptionButton,-1,True,100000000400_Missing data,True,Mean,False,,, OptionButtonAll,OptionButton,-1,True,100000000200_Missing data,True,For all samples,False,,, OptionButtonRestrict,OptionButton,0,True,100000010200_Missing data,True,For the corresponding sample,False,,, OptionButtonMVRefuse,OptionButton,-1,True,100000000000_Missing data,True,Do not accept missing data,False,,, OptionButton_W,OptionButton,0,True,000000000001_General,True,Workbook,False,,, OptionButton_R,OptionButton,-1,True,000000010001_General,True,Range,False,,, OptionButton_S,OptionButton,0,True,000000020001_General,True,Sheet,False,,, RefEdit_R,RefEdit0,'Sheet3'!$M$47,True,000000000101_General,True,Range:,False,,0,0 CheckBoxVarLabels,CheckBox,0,True,000000000201_General,True,Sample labels,False,,, CheckBox_W,CheckBox,0,True,000000000301_General,True,Weights,False,,, RefEdit_W,RefEdit,,True,000000000401_General,True,Weights:,False,,, TextBox_Resample,TextBox,1000,True,000000000000_General,True,Number of samples:,False,,, RefEdit_X,RefEdit0,'Sheet3'!$I$2:$K$46,True,000000000100_General,True,Quantitative data:,False,,45,3 ComboBoxMethod,ComboBox,0,True,000000010300_General,True,Method:,False,,, TextBox_obs,TextBox,5,True,000000000500_General,True,Sample size:,False,,, ComboBoxChartY,ComboBox,2,True,300000000101_Charts,True,Ordinate of the histograms:,False,,,  1 ListBox 118 ListBoxQuanti  1 22  Sum,0 Mean,-1 Variance (n),-1 Variance (n-1),-1 Standard deviation (n),-1 Standard deviation (n-1),-1 Median,-1 1st Quartile,0 3rd Quartile,0 Variation coefficient,0 Standard error of the mean,0 Mean absolute deviation,0 Median absolute deviation,-1 Geometric mean,0 Geometric standard deviation,0 Harmonic mean,0 1-Percentile,0 99-Percentile,0 2.5-Percentile,0 97.5-Percentile,0 5-Percentile,0 95-Percentile,0  890520  Pizzerie n=45*SEP*Summary statistics*SEP*$M$58 Pizzerie n=45*SEP*Results of the resampling (X1)*SEP*$M$66 Pizzerie n=45*SEP*Results of the resampling (X2)*SEP*$M$78 Pizzerie n=45*SEP*Results of the resampling (X3)*SEP*$M$90 | | 18.440 | | | 17.809 | | | | 3.208 | | | |  | |  | | |  |  |  |
| Standard deviation (n-1) | | 18.701 | | | 18.061 | | | | 3.253 | | | |  | |  | | |  |  |  |
| Median | | 20.000 | | | 18.173 | | | | 2.315 | | | |  | |  | | |  |  |  |
| Median absolute deviation | | 5.000 | | | 4.475 | | | | 1.666 | | | |  | |  | | |  |  |  |
|  | |  | | |  | | | |  | | | |  | |  | | |  |  |  |
| **Results of the resampling (OBS 2):** | | | | | | |  | | | |  |  | |  | | |  |  |  |  |
|  |  | | |  | | |  | | | |  |  | |  | | |  |  |  |  |
| Parameters | Estimator | | | Estimator (Bootstrap) | | | Standard deviation (Bootstrap) | | | |  |  | |  | | |  |  |  |  |
| Mean | 48.056 | | | 48.117 | | | 2.244 | | | |  |  | |  | | |  |  |  |  |
| Variance (n) | 198.997 | | | 193.792 | | | 48.295 | | | |  |  | |  | | |  |  |  |  |
| Variance (n-1) | 204.683 | | | 199.329 | | | 49.675 | | | |  |  | |  | | |  |  |  |  |
| Standard deviation (n) | 14.107 | | | 13.814 | | | 1.726 | | | |  |  | |  | | |  |  |  |  |
| Standard deviation (n-1) | 14.307 | | | 14.010 | | | 1.750 | | | |  |  | |  | | |  |  |  |  |
| Median | 45.000 | | | 47.820 | | | 5.289 | | | |  |  | |  | | |  |  |  |  |
| Median absolute deviation | 15.000 | | | 12.178 | | | 4.473 | | | |  |  | |  | | |  |  |  |  |
|  |  | | |  | | |  | | | |  |  | |  | | |  |  |  |  |
| **Mood test:** | | |  | | |  | |  | |  |  | |  | | |  | | |  |  |
|  | | |  | | |  | |  | |  |  | |  | | |  | | |  |  |
| U | | | 22.239 | | |  | |  | |  |  | |  | | |  | | |  |  |
| Critical value  RunProcRSP Form118.txt CheckBoxTrans,CheckBox,0,False,03,False,Trans,False,,, ListBoxQuanti,ListBox,,True,200000000000_Outputs,True,,False,,, CheckBoxHist,CheckBox,0,True,300000000000_Charts,True,Histograms,False,,, OptionButtonHistBar,OptionButton,-1,True,300000000100_Charts,True,Bars,False,,, OptionButtonHistCont,OptionButton,0,True,300000010100_Charts,True,Continuous line,False,,, CheckBoxCum,CheckBox,0,True,300000000200_Charts,True,Cumulative histograms,False,,, OptionButtonHisBased,OptionButton,-1,True,300000000300_Charts,True,Based on the histogram,False,,, OptionButtonECDF,OptionButton,0,True,300000010300_Charts,True,Empirical cumulative distribution,False,,, CheckBoxRData,CheckBox,0,True,200000000008_Outputs,True,Resamples,False,,, TextBoxConfPer,TextBox,95,True,200000000003_Outputs,True,Confidence interval (%):,False,,, CheckBoxRStat,CheckBox,0,True,200000000007_Outputs,True,Resampled statistics,False,,, CheckBoxNormInt,CheckBox,0,True,200000000004_Outputs,True,Standard bootstrap interval,False,,, CheckBoxPercInt,CheckBox,0,True,200000000005_Outputs,True,Simple percentile interval,False,,, CheckBoxBiasInt,CheckBox,0,True,200000000006_Outputs,True,B.C. percentile interval,False,,, OptionButton_MVRemove,OptionButton,0,True,100000000100_Missing data,True,Remove the observations,False,,, OptionButton_MVEstimate,OptionButton,0,True,100000000300_Missing data,True,Estimate missing data,False,,, OptionButton_MeanMode,OptionButton,-1,True,100000000400_Missing data,True,Mean,False,,, OptionButtonAll,OptionButton,-1,True,100000000200_Missing data,True,For all samples,False,,, OptionButtonRestrict,OptionButton,0,True,100000010200_Missing data,True,For the corresponding sample,False,,, OptionButtonMVRefuse,OptionButton,-1,True,100000000000_Missing data,True,Do not accept missing data,False,,, OptionButton_W,OptionButton,0,True,000000000001_General,True,Workbook,False,,, OptionButton_R,OptionButton,-1,True,000000010001_General,True,Range,False,,, OptionButton_S,OptionButton,0,True,000000020001_General,True,Sheet,False,,, RefEdit_R,RefEdit0,'Sheet3'!$M$47,True,000000000101_General,True,Range:,False,,0,0 CheckBoxVarLabels,CheckBox,-1,True,000000000201_General,True,Sample labels,False,,, CheckBox_W,CheckBox,0,True,000000000301_General,True,Weights,False,,, RefEdit_W,RefEdit,,True,000000000401_General,True,Weights:,False,,, TextBox_Resample,TextBox,1000,True,000000000000_General,True,Number of samples:,False,,, RefEdit_X,RefEdit0,'Sheet3'!$I$2:$K$46,True,000000000100_General,True,Quantitative data:,False,,36,3 ComboBoxMethod,ComboBox,0,True,000000010300_General,True,Method:,False,,, TextBox_obs,TextBox,5,True,000000000500_General,True,Sample size:,False,,, ComboBoxChartY,ComboBox,2,True,300000000101_Charts,True,Ordinate of the histograms:,False,,,  1 ListBox 118 ListBoxQuanti  1 22  Sum,0 Mean,-1 Variance (n),-1 Variance (n-1),-1 Standard deviation (n),-1 Standard deviation (n-1),-1 Median,-1 1st Quartile,0 3rd Quartile,0 Variation coefficient,0 Standard error of the mean,0 Mean absolute deviation,0 Median absolute deviation,-1 Geometric mean,0 Geometric standard deviation,0 Harmonic mean,0 1-Percentile,0 99-Percentile,0 2.5-Percentile,0 97.5-Percentile,0 5-Percentile,0 95-Percentile,0  RunProcRSP Form118.txt CheckBoxTrans,CheckBox,0,False,03,False,Trans,False,,, ListBoxQuanti,ListBox,,True,200000000000_Outputs,True,,False,,, CheckBoxHist,CheckBox,0,True,300000000000_Charts,True,Histograms,False,,, OptionButtonHistBar,OptionButton,-1,True,300000000100_Charts,True,Bars,False,,, OptionButtonHistCont,OptionButton,0,True,300000010100_Charts,True,Continuous line,False,,, CheckBoxCum,CheckBox,0,True,300000000200_Charts,True,Cumulative histograms,False,,, OptionButtonHisBased,OptionButton,-1,True,300000000300_Charts,True,Based on the histogram,False,,, OptionButtonECDF,OptionButton,0,True,300000010300_Charts,True,Empirical cumulative distribution,False,,, CheckBoxRData,CheckBox,0,True,200000000008_Outputs,True,Resamples,False,,, TextBoxConfPer,TextBox,95,True,200000000003_Outputs,True,Confidence interval (%):,False,,, CheckBoxRStat,CheckBox,0,True,200000000007_Outputs,True,Resampled statistics,False,,, CheckBoxNormInt,CheckBox,0,True,200000000004_Outputs,True,Standard bootstrap interval,False,,, CheckBoxPercInt,CheckBox,0,True,200000000005_Outputs,True,Simple percentile interval,False,,, CheckBoxBiasInt,CheckBox,0,True,200000000006_Outputs,True,B.C. percentile interval,False,,, OptionButton_MVRemove,OptionButton,0,True,100000000100_Missing data,True,Remove the observations,False,,, OptionButton_MVEstimate,OptionButton,0,True,100000000300_Missing data,True,Estimate missing data,False,,, OptionButton_MeanMode,OptionButton,-1,True,100000000400_Missing data,True,Mean,False,,, OptionButtonAll,OptionButton,-1,True,100000000200_Missing data,True,For all samples,False,,, OptionButtonRestrict,OptionButton,0,True,100000010200_Missing data,True,For the corresponding sample,False,,, OptionButtonMVRefuse,OptionButton,-1,True,100000000000_Missing data,True,Do not accept missing data,False,,, OptionButton_W,OptionButton,0,True,000000000001_General,True,Workbook,False,,, OptionButton_R,OptionButton,-1,True,000000010001_General,True,Range,False,,, OptionButton_S,OptionButton,0,True,000000020001_General,True,Sheet,False,,, RefEdit_R,RefEdit0,'Sheet3'!$M$47,True,000000000101_General,True,Range:,False,,0,0 CheckBoxVarLabels,CheckBox,-1,True,000000000201_General,True,Sample labels,False,,, CheckBox_W,CheckBox,0,True,000000000301_General,True,Weights,False,,, RefEdit_W,RefEdit,,True,000000000401_General,True,Weights:,False,,, TextBox_Resample,TextBox,1000,True,000000000000_General,True,Number of samples:,False,,, RefEdit_X,RefEdit0,'Sheet3'!$I$2:$I$37,True,000000000100_General,True,Quantitative data:,False,,36,1 ComboBoxMethod,ComboBox,0,True,000000010300_General,True,Method:,False,,, TextBox_obs,TextBox,5,True,000000000500_General,True,Sample size:,False,,, ComboBoxChartY,ComboBox,2,True,300000000101_Charts,True,Ordinate of the histograms:,False,,,  1 ListBox 118 ListBoxQuanti  1 22  Sum,0 Mean,-1 Variance (n),-1 Variance (n-1),-1 Standard deviation (n),-1 Standard deviation (n-1),-1 Median,-1 1st Quartile,0 3rd Quartile,0 Variation coefficient,0 Standard error of the mean,0 Mean absolute deviation,0 Median absolute deviation,-1 Geometric mean,0 Geometric standard deviation,0 Harmonic mean,0 1-Percentile,0 99-Percentile,0 2.5-Percentile,0 97.5-Percentile,0 5-Percentile,0 95-Percentile,0  634421  RunProcRSP Form118.txt CheckBoxTrans,CheckBox,0,False,03,False,Trans,False,,, ListBoxQuanti,ListBox,,True,200000000000_Outputs,True,,False,,, CheckBoxHist,CheckBox,0,True,300000000000_Charts,True,Histograms,False,,, OptionButtonHistBar,OptionButton,-1,True,300000000100_Charts,True,Bars,False,,, OptionButtonHistCont,OptionButton,0,True,300000010100_Charts,True,Continuous line,False,,, CheckBoxCum,CheckBox,0,True,300000000200_Charts,True,Cumulative histograms,False,,, OptionButtonHisBased,OptionButton,-1,True,300000000300_Charts,True,Based on the histogram,False,,, OptionButtonECDF,OptionButton,0,True,300000010300_Charts,True,Empirical cumulative distribution,False,,, CheckBoxRData,CheckBox,0,True,200000000008_Outputs,True,Resamples,False,,, TextBoxConfPer,TextBox,95,True,200000000003_Outputs,True,Confidence interval (%):,False,,, CheckBoxRStat,CheckBox,0,True,200000000007_Outputs,True,Resampled statistics,False,,, CheckBoxNormInt,CheckBox,0,True,200000000004_Outputs,True,Standard bootstrap interval,False,,, CheckBoxPercInt,CheckBox,0,True,200000000005_Outputs,True,Simple percentile interval,False,,, CheckBoxBiasInt,CheckBox,0,True,200000000006_Outputs,True,B.C. percentile interval,False,,, OptionButton_MVRemove,OptionButton,0,True,100000000100_Missing data,True,Remove the observations,False,,, OptionButton_MVEstimate,OptionButton,0,True,100000000300_Missing data,True,Estimate missing data,False,,, OptionButton_MeanMode,OptionButton,-1,True,100000000400_Missing data,True,Mean,False,,, OptionButtonAll,OptionButton,-1,True,100000000200_Missing data,True,For all samples,False,,, OptionButtonRestrict,OptionButton,0,True,100000010200_Missing data,True,For the corresponding sample,False,,, OptionButtonMVRefuse,OptionButton,-1,True,100000000000_Missing data,True,Do not accept missing data,False,,, OptionButton_W,OptionButton,0,True,000000000001_General,True,Workbook,False,,, OptionButton_R,OptionButton,-1,True,000000010001_General,True,Range,False,,, OptionButton_S,OptionButton,0,True,000000020001_General,True,Sheet,False,,, RefEdit_R,RefEdit0,'Sheet3'!$M$43,True,000000000101_General,True,Range:,False,,0,0 CheckBoxVarLabels,CheckBox,-1,True,000000000201_General,True,Sample labels,False,,, CheckBox_W,CheckBox,0,True,000000000301_General,True,Weights,False,,, RefEdit_W,RefEdit,,True,000000000401_General,True,Weights:,False,,, TextBox_Resample,TextBox,1000,True,000000000000_General,True,Number of samples:,False,,, RefEdit_X,RefEdit0,'Sheet3'!$J$2:$J$37,True,000000000100_General,True,Quantitative data:,False,,36,1 ComboBoxMethod,ComboBox,0,True,000000010300_General,True,Method:,False,,, TextBox_obs,TextBox,5,True,000000000500_General,True,Sample size:,False,,, ComboBoxChartY,ComboBox,2,True,300000000101_Charts,True,Ordinate of the histograms:,False,,,  1 ListBox 118 ListBoxQuanti  1 22  Sum,0 Mean,-1 Variance (n),-1 Variance (n-1),-1 Standard deviation (n),-1 Standard deviation (n-1),-1 Median,-1 1st Quartile,0 3rd Quartile,0 Variation coefficient,0 Standard error of the mean,0 Mean absolute deviation,0 Median absolute deviation,-1 Geometric mean,0 Geometric standard deviation,0 Harmonic mean,0 1-Percentile,0 99-Percentile,0 2.5-Percentile,0 97.5-Percentile,0 5-Percentile,0 95-Percentile,0  411545  Pizzerie n=45*SEP*Summary statistics*SEP*$M$54 Pizzerie n=45*SEP*Results of the resampling (20)*SEP*$M$60 | | | 3.841 | | |  | |  | |  |  | |  | | |  | | |  |  |
| DF | | | 1.000 | | |  | |  | |  |  | |  | | |  | | |  |  |
| p-value | | | < 0.0001 | | |  | |  | |  |  | |  | | |  | | |  |  |
| alpha | | | 0.05 | | |  | |  | |  |  | |  | | |  | | |  |  |
| The p-value has been computed using 10000 Monte Carlo simulations. Time elapsed: 0s. | | | | | | | | | | | | | | | |  | | |  |  |
|  | | |  | | |  | |  | |  |  | |  | | |  | | |  |  |
| Test interpretation: | | |  | | |  | |  | |  |  | |  | | |  | | |  |  |
| H0: The medians of OBS 1 and OBS 2 are equal. | | | | | | | |  | |  |  | |  | | |  | | |  |  |
| Ha: Medians of OBS 1 and OBS 2 are not equal | | | | | | | |  | |  |  | |  | | |  | | |  |  |
| 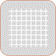As the computed p-value is lower than the significance level alpha=0.05,  one should reject the null hypothesis H0, and accept the alternative hypothesis Ha. | | | | | | | | | | | | | | | | | | | |  |
|  |  |  |  |  |  |  |  |  |  |  |  |  |  |  |  |  |  |  |  |  |

| **Fine dining restaurants** | **OBS 1** | **OBS 2** |
| --- | --- | --- |
| 5 Maggio | 60 | 90 |
| Cavour modo | 45 | 120 |
| Cottura 9' | 60 | 90 |
| Da Gibba | 90 | 150 |
| I tre merli | 90 | 90 |
| Il Baluardo | 60 | 150 |
| Il gelsomino | 90 | 90 |
| Il Sette Genova | 60 | 120 |
| Ittiturismo all'Amo | 60 | 150 |
| Kapperi | 60 | 150 |
| La Ola | 60 | 150 |
| La Terrazza | 60 | 120 |
| Le cantine | 90 | 150 |
| Le cicale trattoria | 60 | 120 |
| Le colonne | 60 | 120 |
| Mangiabuono | 60 | 90 |
| Osteria Vico Palla | 90 | 150 |
| Ostetrattoria | 90 | 90 |
| Paradiso | 25 | 90 |
| Rio Samba | 60 | 60 |
| Rosmarino | 60 | 90 |
| Santa Teresa | 45 | 90 |
| Soho | 60 | 120 |
| The Cook | 60 | 90 |
| Trattoria Osvaldo | 90 | 120 |
| Trattoria Osvaldo | 90 | 90 |
| Trattoria Ugo | 60 | 90 |
| Trattoria Ugo | 60 | 60 |
| Vegia Zena | 60 | 120 |
| Vegia Zena | 60 | 90 |
| Vivarelli | 60 | 90 |
| Vivarelli | 90 | 120 |

| **Descriptives** | **OBS 1** | **OBS 2** |  |  |  |  |  |  |  |
| --- | --- | --- | --- | --- | --- | --- | --- | --- | --- |
| Nbr. of observations | 32 | 32 |  |  |  |  |  |  |  |
| Nbr. of missing values | 0 | 0 |  |  |  |  |  |  |  |
| Obs. without missing data | 32 | 32 |  |  |  |  |  |  |  |
| Minimum | 25.000 | 60.000 |  |  |  |  |  |  |  |
| Maximum | 90.000 | 150.000 |  |  |  |  |  |  |  |
| Freq. of minimum | 1 | 2 |  |  |  |  |  |  |  |
| Freq. of maximum | 9 | 7 |  |  |  |  |  |  |  |
| Median | 60.000 | 105.000 |  |  |  |  |  |  |  |
| Mean | 66.406 | 109.688 |  |  |  |  |  |  |  |
| Variance (n-1) | 272.959 | 732.157 |  |  |  |  |  |  |  |
| Standard deviation (n-1) | 16.521 | 27.058 |  |  |  |  |  |  |  |
| Median absolute deviation | 0.000 | 15.000 |  |  |  |  |  |  |  |
|  |  |  |  |  |  |  |  |  |  |
| **Shapiro-Wilk test (OBS 1):** |  |  |  |  |  |  |  |  |  |
|  |  |  |  |  |  |  |  |  |  |
| W | 0.752 |  |  |  |  |  |  |  |  |
| p-value (Two-tailed) | **<0.0001** |  |  |  |  |  |  |  |  |
| alpha | 0.05 |  |  |  |  |  |  |  |  |
| Fine dining restaurants n=34*SEP*Summary statistics*SEP*$M$47 Fine dining restaurants n=34*SEP*Results of the resampling (X1)*SEP*$M$53 |  |  |  |  |  |  |  |  |  |
| Test interpretation: |  |  |  |  |  |  |  |  |  |
| H0: The variable from which the sample was extracted follows a Normal distribution. | | | | | |  |  |  |  |
| Ha: The variable from which the sample was extracted does not follow a Normal distribution. | | | | | | |  |  |  |
| As the computed p-value is lower than the significance level alpha=0.05,  one should reject the null hypothesis H0, and accept the alternative hypothesis Ha. | | | | | | | | |  |
|  |  |  |  |  |  |  |  |  |  |

| **Shapiro-Wilk test (OBS 2):** | | | | | | |  | |  | |  | |  | | |  | | |  | | |  |  |
| --- | --- | --- | --- | --- | --- | --- | --- | --- | --- | --- | --- | --- | --- | --- | --- | --- | --- | --- | --- | --- | --- | --- | --- |
|  | | | |  | | |  | |  | |  | |  | | |  | | |  | | |  |  |
| W | | | | 0.852 | | |  | |  | |  | |  | | |  | | |  | | |  |  |
| p-value (Two-tailed) | | | | **0.000** | | |  | |  | |  | |  | | |  | | |  | | |  |  |
| alpha | | | | 0.05 | | |  | |  | |  | |  | | |  | | |  | | |  |  |
|  | | | |  | | |  | |  | |  | |  | | |  | | |  | | |  |  |
| Test interpretation: | | | |  | | |  | |  | |  | |  | | |  | | |  | | |  |  |
| H0: The variable from which the sample was extracted follows a Normal distribution. | | | | | | | | | | | | | | | | | | |  | | |  |  |
| Ha: The variable from which the sample was extracted does not follow a Normal distribution. | | | | | | | | | | | | | | | | | | | | | |  |  |
| As the computed p-value is lower than the significance level alpha=0.05,  one should reject the null hypothesis H0, and accept the alternative hypothesis Ha. | | | | | | | | | | | | | | | | | | | | | | |  |
|  |  |  |  |  |  |  |  |  |  |  |  |  |  |  |  |  |  |  |  |  |  |  |  |
|  | | | |  | | |  | |  | |  | |  | | |  | | |  | | |  |  |
| **Results of the resampling (OBS 1):** | | | | | |  | | | |  | | | | |  | | |  | | |  |  |  |
| 777618 | | |  | | |  | | | |  | | | | |  | | |  | | |  |  |  |
| Parameters | | | Estimator | | | Estimator (Bootstrap) | | | | Standard deviation (Bootstrap) | | | | |  | | |  | | |  |  |  |
| Mean  RunProcRSP Form118.txt CheckBoxTrans,CheckBox,0,False,03,False,Trans,False,,, ListBoxQuanti,ListBox,,True,200000000000_Outputs,True,,False,,, CheckBoxHist,CheckBox,0,True,300000000000_Charts,True,Histograms,False,,, OptionButtonHistBar,OptionButton,-1,True,300000000100_Charts,True,Bars,False,,, OptionButtonHistCont,OptionButton,0,True,300000010100_Charts,True,Continuous line,False,,, CheckBoxCum,CheckBox,0,True,300000000200_Charts,True,Cumulative histograms,False,,, OptionButtonHisBased,OptionButton,-1,True,300000000300_Charts,True,Based on the histogram,False,,, OptionButtonECDF,OptionButton,0,True,300000010300_Charts,True,Empirical cumulative distribution,False,,, CheckBoxRData,CheckBox,0,True,200000000008_Outputs,True,Resamples,False,,, TextBoxConfPer,TextBox,95,True,200000000003_Outputs,True,Confidence interval (%):,False,,, CheckBoxRStat,CheckBox,0,True,200000000007_Outputs,True,Resampled statistics,False,,, CheckBoxNormInt,CheckBox,0,True,200000000004_Outputs,True,Standard bootstrap interval,False,,, CheckBoxPercInt,CheckBox,0,True,200000000005_Outputs,True,Simple percentile interval,False,,, CheckBoxBiasInt,CheckBox,0,True,200000000006_Outputs,True,B.C. percentile interval,False,,, OptionButton_MVRemove,OptionButton,0,True,100000000100_Missing data,True,Remove the observations,False,,, OptionButton_MVEstimate,OptionButton,0,True,100000000300_Missing data,True,Estimate missing data,False,,, OptionButton_MeanMode,OptionButton,-1,True,100000000400_Missing data,True,Mean,False,,, OptionButtonAll,OptionButton,-1,True,100000000200_Missing data,True,For all samples,False,,, OptionButtonRestrict,OptionButton,0,True,100000010200_Missing data,True,For the corresponding sample,False,,, OptionButtonMVRefuse,OptionButton,-1,True,100000000000_Missing data,True,Do not accept missing data,False,,, OptionButton_W,OptionButton,0,True,000000000001_General,True,Workbook,False,,, OptionButton_R,OptionButton,-1,True,000000010001_General,True,Range,False,,, OptionButton_S,OptionButton,0,True,000000020001_General,True,Sheet,False,,, RefEdit_R,RefEdit0,'Sheet4'!$M$36,True,000000000101_General,True,Range:,False,,1,1 CheckBoxVarLabels,CheckBox,0,True,000000000201_General,True,Sample labels,False,,, CheckBox_W,CheckBox,0,True,000000000301_General,True,Weights,False,,, RefEdit_W,RefEdit,,True,000000000401_General,True,Weights:,False,,, TextBox_Resample,TextBox,1000,True,000000000000_General,True,Number of samples:,False,,, RefEdit_X,RefEdit0,'Sheet4'!$I$2:$I$35,True,000000000100_General,True,Quantitative data:,False,,34,1 ComboBoxMethod,ComboBox,0,True,000000010300_General,True,Method:,False,,, TextBox_obs,TextBox,5,True,000000000500_General,True,Sample size:,False,,, ComboBoxChartY,ComboBox,2,True,300000000101_Charts,True,Ordinate of the histograms:,False,,,  1 ListBox 118 ListBoxQuanti  1 22  Sum,0 Mean,-1 Variance (n),-1 Variance (n-1),-1 Standard deviation (n),-1 Standard deviation (n-1),-1 Median,-1 1st Quartile,0 3rd Quartile,0 Variation coefficient,0 Standard error of the mean,0 Mean absolute deviation,0 Median absolute deviation,-1 Geometric mean,0 Geometric standard deviation,0 Harmonic mean,0 1-Percentile,0 99-Percentile,0 2.5-Percentile,0 97.5-Percentile,0 5-Percentile,0 95-Percentile,0  574342  Fine dining restaurants n=34*SEP*Summary statistics*SEP*$M$47 Fine dining restaurants n=34*SEP*Results of the resampling (X1)*SEP*$M$53 | | | 66.406 | | | 66.336 | | | | 2.881 | | | | |  | | |  | | |  |  |  |
| Variance (n) | | | 264.429 | | | 256.073 | | | | 60.418 | | | | |  | | |  | | |  |  |  |
| Variance (n-1) | | | 272.959 | | | 264.334 | | | | 62.367 | | | | |  | | |  | | |  |  |  |
| Standard deviation (n) | | | 16.261 | | | 15.888 | | | | 1.910 | | | | |  | | |  | | |  |  |  |
| Standard deviation (n-1) | | | 16.521 | | | 16.142 | | | | 1.940 | | | | |  | | |  | | |  |  |  |
| Median | | | 60.000 | | | 60.090 | | | | 1.498 | | | | |  | | |  | | |  |  |  |
| Median absolute deviation | | | 0.000 | | | 1.103 | | | | 3.792 | | | | |  | | |  | | |  |  |  |
|  | | |  | | |  | | | |  | | | | |  | | |  | | |  |  |  |
| **Results of the resampling (OBS 2):** | | | | | | | |  | | | |  | |  | | |  | | |  |  |  |  |
|  |  |  | | | | | |  | | | |  | |  | | |  | | |  |  |  |  |
| Parameters | Estimator | Estimator (Bootstrap) | | | | | | Standard deviation (Bootstrap) | | | |  | |  | | |  | | |  |  |  |  |
| Mean | 109.688 | 109.562 | | | | | | 4.697 | | | |  | |  | | |  | | |  |  |  |  |
| Variance (n) | 709.277 | 692.334 | | | | | | 128.762 | | | |  | |  | | |  | | |  |  |  |  |
| Variance (n-1) | 732.157 | 714.667 | | | | | | 132.916 | | | |  | |  | | |  | | |  |  |  |  |
| Standard deviation (n) | 26.632 | 26.195 | | | | | | 2.479 | | | |  | |  | | |  | | |  |  |  |  |
| Standard deviation (n-1) | 27.058 | 26.615 | | | | | | 2.518 | | | |  | |  | | |  | | |  |  |  |  |
| Median | 105.000 | 104.805 | | | | | | 14.005 | | | |  | |  | | |  | | |  |  |  |  |
| Median absolute deviation | 15.000 | 20.505 | | | | | | 12.028 | | | |  | |  | | |  | | |  |  |  |  |
|  |  |  | | | | | |  | | | |  | |  | | |  | | |  |  |  |  |
| **Mood test:** | | | | |  | |  | |  | |  | |  | | |  | | |  | | |  |  |
|  | | | | |  | |  | |  | |  | |  | | |  | | |  | | |  |  |
| U  Fine dining restaurants n=34*SEP*Summary statistics*SEP*$M$54 Fine dining restaurants n=34*SEP*Results of the resampling (X1)*SEP*$M$60 | | | | | 18.750 | |  | |  | |  | |  | | |  | | |  | | |  |  |
| Critical value | | | | | 3.841 | |  | |  | |  | |  | | |  | | |  | | |  |  |
| DF | | | | | 1.000 | | 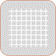 | |  | |  | |  | | |  | | |  | | |  |  |
| p-value | | | | | < 0.0001 | |  | |  | |  | |  | | |  | | |  | | |  |  |
| alpha | | | | | 0.05 | |  | |  | |  | |  | | |  | | |  | | |  |  |
| The p-value has been computed using 10000 Monte Carlo simulations. Time elapsed: 0s. | | | | | | | | | | | | | | | |  | | |  | | |  |  |
|  | | | | |  | |  | |  | |  | |  | | |  | | |  | | |  |  |
| Test interpretation: | | | | |  | |  | |  | |  | |  | | |  | | |  | | |  |  |
| H0: The medians of OBS 1 and OBS 2 are equal.  RunProcRSP Form118.txt CheckBoxTrans,CheckBox,0,False,03,False,Trans,False,,, ListBoxQuanti,ListBox,,True,200000000000_Outputs,True,,False,,, CheckBoxHist,CheckBox,0,True,300000000000_Charts,True,Histograms,False,,, OptionButtonHistBar,OptionButton,-1,True,300000000100_Charts,True,Bars,False,,, OptionButtonHistCont,OptionButton,0,True,300000010100_Charts,True,Continuous line,False,,, CheckBoxCum,CheckBox,0,True,300000000200_Charts,True,Cumulative histograms,False,,, OptionButtonHisBased,OptionButton,-1,True,300000000300_Charts,True,Based on the histogram,False,,, OptionButtonECDF,OptionButton,0,True,300000010300_Charts,True,Empirical cumulative distribution,False,,, CheckBoxRData,CheckBox,0,True,200000000008_Outputs,True,Resamples,False,,, TextBoxConfPer,TextBox,95,True,200000000003_Outputs,True,Confidence interval (%):,False,,, CheckBoxRStat,CheckBox,0,True,200000000007_Outputs,True,Resampled statistics,False,,, CheckBoxNormInt,CheckBox,0,True,200000000004_Outputs,True,Standard bootstrap interval,False,,, CheckBoxPercInt,CheckBox,0,True,200000000005_Outputs,True,Simple percentile interval,False,,, CheckBoxBiasInt,CheckBox,0,True,200000000006_Outputs,True,B.C. percentile interval,False,,, OptionButton_MVRemove,OptionButton,0,True,100000000100_Missing data,True,Remove the observations,False,,, OptionButton_MVEstimate,OptionButton,0,True,100000000300_Missing data,True,Estimate missing data,False,,, OptionButton_MeanMode,OptionButton,-1,True,100000000400_Missing data,True,Mean,False,,, OptionButtonAll,OptionButton,-1,True,100000000200_Missing data,True,For all samples,False,,, OptionButtonRestrict,OptionButton,0,True,100000010200_Missing data,True,For the corresponding sample,False,,, OptionButtonMVRefuse,OptionButton,-1,True,100000000000_Missing data,True,Do not accept missing data,False,,, OptionButton_W,OptionButton,0,True,000000000001_General,True,Workbook,False,,, OptionButton_R,OptionButton,-1,True,000000010001_General,True,Range,False,,, OptionButton_S,OptionButton,0,True,000000020001_General,True,Sheet,False,,, RefEdit_R,RefEdit0,'Sheet4'!$M$43,True,000000000101_General,True,Range:,False,,0,0 CheckBoxVarLabels,CheckBox,0,True,000000000201_General,True,Sample labels,False,,, CheckBox_W,CheckBox,0,True,000000000301_General,True,Weights,False,,, RefEdit_W,RefEdit,,True,000000000401_General,True,Weights:,False,,, TextBox_Resample,TextBox,1000,True,000000000000_General,True,Number of samples:,False,,, RefEdit_X,RefEdit0,'Sheet4'!$J$2:$J$35,True,000000000100_General,True,Quantitative data:,False,,34,1 ComboBoxMethod,ComboBox,0,True,000000010300_General,True,Method:,False,,, TextBox_obs,TextBox,5,True,000000000500_General,True,Sample size:,False,,, ComboBoxChartY,ComboBox,2,True,300000000101_Charts,True,Ordinate of the histograms:,False,,,  1 ListBox 118 ListBoxQuanti  1 22  Sum,0 Mean,-1 Variance (n),-1 Variance (n-1),-1 Standard deviation (n),-1 Standard deviation (n-1),-1 Median,-1 1st Quartile,0 3rd Quartile,0 Variation coefficient,0 Standard error of the mean,0 Mean absolute deviation,0 Median absolute deviation,-1 Geometric mean,0 Geometric standard deviation,0 Harmonic mean,0 1-Percentile,0 99-Percentile,0 2.5-Percentile,0 97.5-Percentile,0 5-Percentile,0 95-Percentile,0  187751 | | | | | | |  | |  | |  | |  | | |  | | |  | | |  |  |
| Ha: Medians of OBS 1 and OBS 2 are not equal | | | | | | |  | |  | |  | |  | | |  | | |  | | |  |  |
| 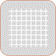As the computed p-value is lower than the significance level alpha=0.05,  one should reject the null hypothesis H0, and accept the alternative hypothesis Ha. | | | | | | | | | | | | | | | | | | | | | | |  |
|  |  |  |  |  |  |  |  |  |  |  |  |  |  |  |  |  |  |  |  |  |  |  |  |
|  | | | | |  | |  | |  | |  | |  | | |  | | |  | | |  |  |
|  | | | | |  | |  | |  | |  | |  | | |  | | |  | | |  |  |

| **Food supermarkets** | **OBS 1** | **OBS 2** |
| --- | --- | --- |
| Basko Passo Antiochia | 15 | 15 |
| Basko Piazza Sopranis | 25 | 20 |
| Basko Via Barabino | 20 | 20 |
| Basko Via Barchetta | 25 | 20 |
| Basko Via Bertolotti | 30 | 25 |
| Basko Via Cavallotti | 25 | 25 |
| Basko Via Cavour | 20 | 25 |
| Basko Via Degola | 20 | 20 |
| Basko Via Emilia | 25 | 20 |
| Basko Via Langustena | 25 | 25 |
| Basko Via Martiri Libertà | 20 | 20 |
| Basko Via Posalunga | 15 | 15 |
| Basko Via Travi | 20 | 20 |
| Basko Via Vezzani | 25 | 25 |
| Carrefour Campetto | 15 | 15 |
| Carrefour Corso Sardegna | 15 | 15 |
| Carrefour Cso Firenze | 15 | 15 |
| Carrefour Piazza Giusti | 10 | 15 |
| Carrefour Pzza Merani | 15 | 15 |
| Carrefour Pzza Villa | 15 | 15 |
| Carrefour Pzzle Parenzo | 15 | 15 |
| Carrefour Salita S Maria | 15 | 20 |
| Carrefour Via Albaro | 15 | 15 |
| Carrefour Via Bari | 15 | 15 |
| Carrefour Via Bologna | 15 | 15 |
| Carrefour Via Canevari | 15 | 15 |
| Carrefour Via Cantore | 15 | 15 |
| Carrefour Via Casaregis | 15 | 15 |
| Carrefour Via Contubernio | 15 | 15 |
| Carrefour Via De Gasperi | 15 | 15 |
| Carrefour Via delle Bernardine | 15 | 15 |
| Carrefour Via di Canneto | 15 | 15 |
| Carrefour Via Fareggiano | 15 | 15 |
| Carrefour Via Fiasella | 15 | 20 |
| Carrefour Via Fillak | 15 | 15 |
| Carrefour Via Gobetti | 15 | 15 |
| Carrefour Via isonzo | 15 | 15 |
| Carrefour Via Jori | 15 | 15 |
| Carrefour Via Montaldo | 15 | 15 |
| Carrefour Via Montevideo | 10 | 15 |
| Carrefour Via Napoli | 15 | 15 |
| Carrefour Via Paleocapa | 15 | 15 |
| Carrefour Via Quinto | 15 | 15 |
| Carrefour Via Rodi | 15 | 15 |
| Carrefour Via Rota | 20 | 15 |
| Carrefour Via S Agnese | 10 | 20 |
| Carrefour Via S Martino | 15 | 15 |
| Carrefour Via S Vincenzo | 15 | 15 |
| Carrefour Via Tanini | 15 | 15 |
| Carrefour Via Torti | 20 | 15 |
| Carrefour Via Tortosa | 15 | 15 |
| Conad Cso Sardegna | 15 | 20 |
| Conad Piazza Carloforte | 20 | 20 |
| Conad Via Anfossi | 25 | 25 |
| Conad Via Mascagni | 20 | 25 |
| Conad Via Piccone | 20 | 20 |
| Conad Via Trossarelli | 15 | 15 |
| Coop Centro commerciale Bisagno | 25 | 25 |
| Coop Centro commerciale Europa | 20 | 25 |
| Coop Centro commerciale Il Mirto | 20 | 25 |
| Coop Centro commerciale Il Terminal | 25 | 25 |
| Coop Centro Commerciale Le Lampare | 20 | 25 |
| Coop Cso Gastaldi | 25 | 25 |
| Coop Pzza Tre Ponti | 20 | 20 |
| Coop Salita F Da Paola | 20 | 20 |
| Coop Stazione Casella | 20 | 20 |
| Coop Via dei Mille | 20 | 20 |
| Coop Via del Canto | 20 | 20 |
| Coop via del Commercio | 20 | 20 |
| Coop Via delle Viazze | 10 | 10 |
| Coop Via Franceschi | 25 | 25 |
| Coop Via Fumara | 20 | 20 |
| Coop Via Merano | 25 | 25 |
| Coop Via Prà | 20 | 20 |
| Coop Via Rivarolo | 20 | 20 |
| Coop Via Roana | 20 | 20 |
| Coop Via Romairone | 10 | 10 |
| Coop Viale Milite Ignoto | 25 | 25 |
| Doro Cso Armellini | 20 | 20 |
| Doro Cso Martinetti | 15 | 15 |
| Doro Via Carrea | 20 | 20 |
| Doro Via Chiodo | 15 | 15 |
| Doro Via del Commercio | 15 | 15 |
| Doro Via Lomellini | 15 | 15 |
| Ekom Via Archimede | 20 | 20 |
| Ekom Via Biga | 20 | 20 |
| Ekom Via Bobbio | 15 | 15 |
| Ekom Via Borgoratti | 15 | 15 |
| Ekom Via Bottego | 20 | 20 |
| Ekom Via Camozzini | 15 | 15 |
| Ekom Via Campomorone | 15 | 15 |
| Ekom Via Canevari | 15 | 15 |
| Ekom Via Cerruti | 20 | 20 |
| Ekom Via Chiaravagna | 15 | 15 |
| Ekom Via Colombo | 20 | 20 |
| Ekom Via Crimea | 20 | 20 |
| Ekom Via Custo | 20 | 20 |
| Ekom Via De Gaspari | 15 | 15 |
| Ekom Via del Lagaccio | 15 | 15 |
| Ekom Via della Castagna | 15 | 15 |
| Ekom Via Donghi | 15 | 15 |
| Ekom Via Ferrara | 25 | 25 |
| Ekom Via Gallino | 20 | 20 |
| Ekom Via Giovanni d'Acrì | 25 | 25 |
| Ekom via Isonzo | 20 | 20 |
| Ekom Via Marussig | 20 | 20 |
| Ekom Via Molassana | 20 | 20 |
| Ekom Via Montebruno | 20 | 20 |
| Ekom Via Oberdan | 20 | 20 |
| Ekom Via Oriani | 20 | 20 |
| Ekom Via Passaggi | 20 | 20 |
| Ekom Via Petrella | 20 | 20 |
| Ekom Via Piacenza | 15 | 15 |
| Ekom Via Pinetti | 15 | 15 |
| Ekom Via Ponte Morosini | 15 | 15 |
| Ekom Via Prà | 25 | 25 |
| Ekom Via S Martino | 15 | 15 |
| Ekom Via San Luca | 20 | 20 |
| Ekom Via Torti | 15 | 15 |
| Ekom Via Turati | 15 | 15 |
| Ekom Via Vittorio Veneto | 15 | 15 |
| iN' Cso Perrone | 20 | 20 |
| iN's Cso de Stefanis | 20 | 20 |
| iN's Mura di s Chiara | 20 | 20 |
| iN's Pzza Caroli | 15 | 15 |
| iN's Pzza Giusti | 20 | 20 |
| iN's Via Buranello | 20 | 20 |
| iN's Via Cantore | 15 | 15 |
| iN's Via Casaregis | 15 | 15 |
| iN's Via Centurione | 25 | 25 |
| iN's Via Donghi | 15 | 15 |
| iN's Via Fusinato | 20 | 20 |
| iN's Via Galata | 20 | 20 |
| iN's Via Giovanni Battista | 25 | 25 |
| iN's Via Jori | 15 | 15 |
| iN's Via Malfettani | 20 | 20 |
| iN's Via Mandoli | 15 | 15 |
| iN's Via Merano | 15 | 15 |
| iN's Via Monticelli | 25 | 25 |
| iN's Via Paggi | 20 | 20 |
| iN's Via Parodi | 25 | 25 |
| iN's Via Piacenza | 15 | 15 |
| iN's Via S Martino | 20 | 20 |
| iN's Via Storace | 15 | 15 |
| iN's Via Vecchia Filanda | 20 | 20 |
| PAM Via Cantore | 15 | 15 |
| PAM Via Chiaravagna | 25 | 20 |
| PAM Via del Lagaccio | 20 | 20 |
| PAM Via Fiasella | 15 | 15 |
| PAM Via Galata | 15 | 15 |
| PAM Via Manuzio | 25 | 25 |
| PAM Via Porta degli Archi | 20 | 20 |
| PAM Via Ruspoli | 15 | 20 |
| PAM Via Sestri | 15 | 15 |
| PAM Via Verità | 25 | 15 |

| **Descriptives** | | | **OBS 1** | | **OBS 2** | |  | |  | | |  | |  | |  | |  | | |  |  |
| --- | --- | --- | --- | --- | --- | --- | --- | --- | --- | --- | --- | --- | --- | --- | --- | --- | --- | --- | --- | --- | --- | --- |
| Nbr. of observations | | | 155 | | 155 | |  | |  | | |  | |  | |  | |  | | |  |  |
| Nbr. of missing values | | | 0 | | 0 | |  | |  | | |  | |  | |  | |  | | |  |  |
| Obs. without missing data | | | 155 | | 155 | |  | |  | | |  | |  | |  | |  | | |  |  |
| Minimum | | | 10.000 | | 10.000 | |  | |  | | |  | |  | |  | |  | | |  |  |
| Maximum | | | 30.000 | | 25.000 | |  | |  | | |  | |  | |  | |  | | |  |  |
| Freq. of minimum | | | 5 | | 2 | |  | |  | | |  | |  | |  | |  | | |  |  |
| Freq. of maximum | | | 1 | | 24 | |  | |  | | |  | |  | |  | |  | | |  |  |
| Median | | | 20.000 | | 20.000 | |  | |  | | |  | |  | |  | |  | | |  |  |
| Mean | | | 18.194 | | 18.323 | |  | |  | | |  | |  | |  | |  | | |  |  |
| Variance (n-1) | | | 15.871 | | 14.051 | |  | |  | | |  | |  | |  | |  | | |  |  |
| Standard deviation (n-1) | | | 3.984 | | 3.748 | |  | |  | | |  | |  | |  | |  | | |  |  |
| Median absolute deviation | | | 5.000 | | 5.000 | |  | |  | | |  | |  | |  | |  | | |  |  |
|  | | |  | |  | |  | |  | | |  | |  | |  | |  | | |  |  |
| **Shapiro-Wilk test (OBS 1):** | | |  | |  | |  | |  | | |  | |  | |  | |  | | |  |  |
|  | | |  | |  | |  | |  | | |  | |  | |  | |  | | |  |  |
| W | | | 0.840 | |  | |  | |  | | |  | |  | |  | |  | | |  |  |
| p-value (Two-tailed) | | | **<0.0001** | |  | |  | |  | | |  | |  | |  | |  | | |  |  |
| alpha | | | 0.05 | |  | |  | |  | | |  | |  | |  | |  | | |  |  |
| Food supermarkets n=155*SEP*Summary statistics*SEP*$M$168  Food supermarkets n=155*SEP*Results of the resampling (X1)*SEP*$M$174 | | |  | |  | |  | |  | | |  | |  | |  | |  | | |  |  |
| Test interpretation: | | |  | |  | |  | |  | | |  | |  | |  | |  | | |  |  |
| H0: The variable from which the sample was extracted follows a Normal distribution. | | | | | | | | | | | | | |  | |  | |  | | |  |  |
| Ha: The variable from which the sample was extracted does not follow a Normal distribution. | | | | | | | | | | | | | | | |  | |  | | |  |  |
| As the computed p-value is lower than the significance level alpha=0.05,  one should reject the null hypothesis H0, and accept the alternative hypothesis Ha. | | | | | | | | | | | | | | | | | | | | |  |  |
|  |  |  |  |  |  |  |  |  |  |  |  |  |  |  |  |  |  |  |  |  |  |  |
|  | | |  | |  | |  | |  | | |  | |  | |  | |  | | |  |  |
| **Shapiro-Wilk test (OBS 2):** | | | |  | |  | |  | | |  | |  | |  | |  | |  |  |  |  |
|  |  | | |  | |  | |  | | |  | |  | |  | |  | |  |  |  |  |
| W | 0.804 | | |  | |  | |  | | |  | |  | |  | |  | |  |  |  |  |
| p-value (Two-tailed) | **<0.0001** | | |  | |  | |  | | |  | |  | |  | |  | |  |  |  |  |
| alpha | 0.05 | | |  | |  | |  | | |  | |  | |  | |  | |  |  |  |  |
|  |  | | |  | |  | |  | | |  | |  | |  | |  | |  |  |  |  |
| Test interpretation: |  | | |  | |  | |  | | |  | |  | |  | |  | |  |  |  |  |
| H0: The variable from which the sample was extracted follows a Normal distribution. | | | | | | | | | | | | | | |  | |  | |  |  |  |  |
| Ha: The variable from which the sample was extracted does not follow a Normal distribution. | | | | | | | | | | | | | | | | |  | |  |  |  |  |
| As the computed p-value is lower than the significance level alpha=0.05,  one should reject the null hypothesis H0, and accept the alternative hypothesis Ha. | | | | | | | | | | | | | | | | | | |  |  |  |  |
|  |  |  |  |  |  |  |  |  |  |  |  |  |  |  |  |  |  |  |  |  |  |  |
|  |  | | |  | |  | |  | | |  | |  | |  | |  | |  |  |  |  |
| **Results of the resampling (OBS 1):** | | | | |  | | | | |  | | | | | |  | |  | | |  | |
|  | |  | | |  | | | | |  | | | | | |  | |  | | |  | |
| Parameters | | Estimator | | | Estimator (Bootstrap) | | | | | Standard deviation (Bootstrap) | | | | | |  | |  | | |  | |
| Mean | | 18.194 | | | 18.195 | | | | | 0.320 | | | | | |  | |  | | |  | |
| Variance (n) | | 15.769 | | | 15.701 | | | | | 1.568 | | | | | |  | |  | | |  | |
| Variance (n-1) | | 15.871 | | | 15.803 | | | | | 1.578 | | | | | |  | |  | | |  | |
| Standard deviation (n) | | 3.971 | | | 3.958 | | | | | 0.197 | | | | | |  | |  | | |  | |
| Standard deviation (n-1) | | 3.984 | | | 3.970 | | | | | 0.198 | | | | | |  | |  | | |  | |
| Median | | 20.000 | | | 17.980 | | | | | 2.455 | | | | | |  | |  | | |  | |
| Median absolute deviation | | 5.000 | | | 4.285 | | | | | 1.751 | | | | | |  | |  | | |  | |
|  | |  | | |  | | | | |  | | | | | |  | |  | | |  | |

| **Results of the resampling (OBS 2):** | | | | | | | |  | | |  | |  | |  |  |  |  |
| --- | --- | --- | --- | --- | --- | --- | --- | --- | --- | --- | --- | --- | --- | --- | --- | --- | --- | --- |
|  |  | |  | | | | |  | | |  | |  | |  |  |  |  |
| Parameters | Estimator | | Estimator (Bootstrap) | | | | | Standard deviation (Bootstrap) | | |  | |  | |  |  |  |  |
| Mean | 18.323 | | 18.323 | | | | | 0.300 | | |  | |  | |  |  |  |  |
| Variance (n) | 13.960 | | 13.882 | | | | | 1.225 | | |  | |  | |  |  |  |  |
| Variance (n-1) | 14.051 | | 13.972 | | | | | 1.233 | | |  | |  | |  |  |  |  |
| Standard deviation (n) | 3.736 | | 3.722 | | | | | 0.165 | | |  | |  | |  |  |  |  |
| Standard deviation (n-1) | 3.748 | | 3.734 | | | | | 0.165 | | |  | |  | |  |  |  |  |
| Median | 20.000 | | 18.640 | | | | | 2.226 | | |  | |  | |  |  |  |  |
| Median absolute deviation | 5.000 | | 4.095 | | | | | 1.926 | | |  | |  | |  |  |  |  |
|  |  | |  | | | | |  | | |  | |  | |  |  |  |  |
| **Mood test:** | | | |  | |  | | |  |  | |  | |  | |  |  |  |
|  | | | |  | |  | | |  |  | |  | |  | |  |  |  |
| U | | | | 0.025 | |  | | |  |  | |  | |  | |  |  |  |
| Critical value  RunProcRSP Form118.txt CheckBoxTrans,CheckBox,0,False,03,False,Trans,False,,, ListBoxQuanti,ListBox,,True,200000000000_Outputs,True,,False,,, CheckBoxHist,CheckBox,0,True,300000000000_Charts,True,Histograms,False,,, OptionButtonHistBar,OptionButton,-1,True,300000000100_Charts,True,Bars,False,,, OptionButtonHistCont,OptionButton,0,True,300000010100_Charts,True,Continuous line,False,,, CheckBoxCum,CheckBox,0,True,300000000200_Charts,True,Cumulative histograms,False,,, OptionButtonHisBased,OptionButton,-1,True,300000000300_Charts,True,Based on the histogram,False,,, OptionButtonECDF,OptionButton,0,True,300000010300_Charts,True,Empirical cumulative distribution,False,,, CheckBoxRData,CheckBox,0,True,200000000008_Outputs,True,Resamples,False,,, TextBoxConfPer,TextBox,95,True,200000000003_Outputs,True,Confidence interval (%):,False,,, CheckBoxRStat,CheckBox,0,True,200000000007_Outputs,True,Resampled statistics,False,,, CheckBoxNormInt,CheckBox,0,True,200000000004_Outputs,True,Standard bootstrap interval,False,,, CheckBoxPercInt,CheckBox,0,True,200000000005_Outputs,True,Simple percentile interval,False,,, CheckBoxBiasInt,CheckBox,0,True,200000000006_Outputs,True,B.C. percentile interval,False,,, OptionButton_MVRemove,OptionButton,0,True,100000000100_Missing data,True,Remove the observations,False,,, OptionButton_MVEstimate,OptionButton,0,True,100000000300_Missing data,True,Estimate missing data,False,,, OptionButton_MeanMode,OptionButton,-1,True,100000000400_Missing data,True,Mean,False,,, OptionButtonAll,OptionButton,-1,True,100000000200_Missing data,True,For all samples,False,,, OptionButtonRestrict,OptionButton,0,True,100000010200_Missing data,True,For the corresponding sample,False,,, OptionButtonMVRefuse,OptionButton,-1,True,100000000000_Missing data,True,Do not accept missing data,False,,, OptionButton_W,OptionButton,0,True,000000000001_General,True,Workbook,False,,, OptionButton_R,OptionButton,-1,True,000000010001_General,True,Range,False,,, OptionButton_S,OptionButton,0,True,000000020001_General,True,Sheet,False,,, RefEdit_R,RefEdit0,'Sheet5'!$M$185,True,000000000101_General,True,Range:,False,,0,0 CheckBoxVarLabels,CheckBox,0,True,000000000201_General,True,Sample labels,False,,, CheckBox_W,CheckBox,0,True,000000000301_General,True,Weights,False,,, RefEdit_W,RefEdit,,True,000000000401_General,True,Weights:,False,,, TextBox_Resample,TextBox,1000,True,000000000000_General,True,Number of samples:,False,,, RefEdit_X,RefEdit0,'Sheet5'!$J$2:$J$156,True,000000000100_General,True,Quantitative data:,False,,155,1 ComboBoxMethod,ComboBox,0,True,000000010300_General,True,Method:,False,,, TextBox_obs,TextBox,5,True,000000000500_General,True,Sample size:,False,,, ComboBoxChartY,ComboBox,2,True,300000000101_Charts,True,Ordinate of the histograms:,False,,,  1 ListBox 118 ListBoxQuanti  1 22  Sum,0 Mean,-1 Variance (n),-1 Variance (n-1),-1 Standard deviation (n),-1 Standard deviation (n-1),-1 Median,-1 1st Quartile,0 3rd Quartile,0 Variation coefficient,0 Standard error of the mean,0 Mean absolute deviation,0 Median absolute deviation,-1 Geometric mean,0 Geometric standard deviation,0 Harmonic mean,0 1-Percentile,0 99-Percentile,0 2.5-Percentile,0 97.5-Percentile,0 5-Percentile,0 95-Percentile,0  976363  Food supermarkets n=155*SEP*Summary statistics*SEP*$M$196  Food supermarkets n=155*SEP*Results of the resampling (X1)*SEP*$M$202 | | | | 3.841 | |  | | |  |  | |  | |  | |  |  |  |
| DF | | | | 1.000 | |  | | |  |  | |  | |  | |  |  |  |
| p-value | | | | 0.877 | |  | | |  |  | |  | |  | |  |  |  |
| alpha | | | | 0.05 | |  | | |  |  | |  | |  | |  |  |  |
| The p-value has been computed using 10000 Monte Carlo simulations. Time elapsed: 0s. | | | | | | | | | | | | | |  | |  |  |  |
|  | | | |  | |  | | |  |  | |  | |  | |  |  |  |
| Test interpretation: | | | |  | |  | | |  |  | |  | |  | |  |  |  |
| H0: The medians of OBS 1 and OBS 2 are equal. | | | | | |  | | |  |  | |  | |  | |  |  |  |
| Ha: Medians of OBS 1 and OBS 2 are not equal | | | | | |  | | |  |  | |  | |  | |  |  |  |
| As the computed p-value is greater than the significance level alpha=0.05,  one cannot reject the null hypothesis H0. | | | | | | | | | | | | | | | | | |  |
|  |  |  |  |  |  |  |  |  |  |  |  |  |  |  |  |  |  |  |
| RunProcRSP Form118.txt CheckBoxTrans,CheckBox,0,False,03,False,Trans,False,,, ListBoxQuanti,ListBox,,True,200000000000_Outputs,True,,False,,, CheckBoxHist,CheckBox,0,True,300000000000_Charts,True,Histograms,False,,, OptionButtonHistBar,OptionButton,-1,True,300000000100_Charts,True,Bars,False,,, OptionButtonHistCont,OptionButton,0,True,300000010100_Charts,True,Continuous line,False,,, CheckBoxCum,CheckBox,0,True,300000000200_Charts,True,Cumulative histograms,False,,, OptionButtonHisBased,OptionButton,-1,True,300000000300_Charts,True,Based on the histogram,False,,, OptionButtonECDF,OptionButton,0,True,300000010300_Charts,True,Empirical cumulative distribution,False,,, CheckBoxRData,CheckBox,0,True,200000000008_Outputs,True,Resamples,False,,, TextBoxConfPer,TextBox,95,True,200000000003_Outputs,True,Confidence interval (%):,False,,, CheckBoxRStat,CheckBox,0,True,200000000007_Outputs,True,Resampled statistics,False,,, CheckBoxNormInt,CheckBox,0,True,200000000004_Outputs,True,Standard bootstrap interval,False,,, CheckBoxPercInt,CheckBox,0,True,200000000005_Outputs,True,Simple percentile interval,False,,, CheckBoxBiasInt,CheckBox,0,True,200000000006_Outputs,True,B.C. percentile interval,False,,, OptionButton_MVRemove,OptionButton,0,True,100000000100_Missing data,True,Remove the observations,False,,, OptionButton_MVEstimate,OptionButton,0,True,100000000300_Missing data,True,Estimate missing data,False,,, OptionButton_MeanMode,OptionButton,-1,True,100000000400_Missing data,True,Mean,False,,, OptionButtonAll,OptionButton,-1,True,100000000200_Missing data,True,For all samples,False,,, OptionButtonRestrict,OptionButton,0,True,100000010200_Missing data,True,For the corresponding sample,False,,, OptionButtonMVRefuse,OptionButton,-1,True,100000000000_Missing data,True,Do not accept missing data,False,,, OptionButton_W,OptionButton,0,True,000000000001_General,True,Workbook,False,,, OptionButton_R,OptionButton,-1,True,000000010001_General,True,Range,False,,, OptionButton_S,OptionButton,0,True,000000020001_General,True,Sheet,False,,, RefEdit_R,RefEdit0,'Sheet5'!$M$213,True,000000000101_General,True,Range:,False,,0,0 CheckBoxVarLabels,CheckBox,0,True,000000000201_General,True,Sample labels,False,,, CheckBox_W,CheckBox,0,True,000000000301_General,True,Weights,False,,, RefEdit_W,RefEdit,,True,000000000401_General,True,Weights:,False,,, TextBox_Resample,TextBox,1000,True,000000000000_General,True,Number of samples:,False,,, RefEdit_X,RefEdit0,'Sheet5'!$K$2:$K$156,True,000000000100_General,True,Quantitative data:,False,,155,1 ComboBoxMethod,ComboBox,0,True,000000010300_General,True,Method:,False,,, TextBox_obs,TextBox,5,True,000000000500_General,True,Sample size:,False,,, ComboBoxChartY,ComboBox,2,True,300000000101_Charts,True,Ordinate of the histograms:,False,,,  1 ListBox 118 ListBoxQuanti  1 22  Sum,0 Mean,-1 Variance (n),-1 Variance (n-1),-1 Standard deviation (n),-1 Standard deviation (n-1),-1 Median,-1 1st Quartile,0 3rd Quartile,0 Variation coefficient,0 Standard error of the mean,0 Mean absolute deviation,0 Median absolute deviation,-1 Geometric mean,0 Geometric standard deviation,0 Harmonic mean,0 1-Percentile,0 99-Percentile,0 2.5-Percentile,0 97.5-Percentile,0 5-Percentile,0 95-Percentile,0  494585  Food supermarkets n=155*SEP*Summary statistics*SEP*$M$224  Food supermarkets n=155*SEP*Results of the resampling (X1)*SEP*$M$230 | | | |  | |  | | |  |  | |  | |  | |  |  |  |
| **Retail shops** | | **OBS 1** | | | **OBS 2** | |  |  |  |  |  |  |  |  |  |  |  |  |
| Acqua & Sapone | | 20 | | | 20 | |  |  |  |  |  |  |  |  |  |  |  |  |
| Antony Morato | | 30 | | | 30 | |  |  |  |  |  |  |  |  |  |  |  |  |
| Arredo 3 store | | 15 | | | 20 | |  |  |  |  |  |  |  |  |  |  |  |  |
| Asta del Mobile | | 20 | | | 20 | |  |  |  |  |  |  |  |  |  |  |  |  |
| Bata | | 25 | | | 25 | |  |  |  |  |  |  |  |  |  |  |  |  |
| Benetton | | 25 | | | 20 | |  |  |  |  |  |  |  |  |  |  |  |  |
| Bricoman | | 20 | | | 20 | |  |  |  |  |  |  |  |  |  |  |  |  |
| Calzedonia | | 20 | | | 20 | |  |  |  |  |  |  |  |  |  |  |  |  |
| Comics corner | | 30 | | | 30 | |  |  |  |  |  |  |  |  |  |  |  |  |
| Dainese | | 25 | | | 25 | |  |  |  |  |  |  |  |  |  |  |  |  |
| Decathlon | | 25 | | | 15 | |  |  |  |  |  |  |  |  |  |  |  |  |
| Dungeon | | 25 | | | 20 | |  |  |  |  |  |  |  |  |  |  |  |  |
| Expert | | 20 | | | 20 | |  |  |  |  |  |  |  |  |  |  |  |  |
| Flying Tiger Copenhagen | | 10 | | | 20 | |  |  |  |  |  |  |  |  |  |  |  |  |
| Globo | | 25 | | | 25 | |  |  |  |  |  |  |  |  |  |  |  |  |
| Guess | | 10 | | | 25 | |  |  |  |  |  |  |  |  |  |  |  |  |
| H&M | | 30 | | | 30 | |  |  |  |  |  |  |  |  |  |  |  |  |
| Il libraccio | | 20 | | | 30 | |  |  |  |  |  |  |  |  |  |  |  |  |
| Lego store | | 20 | | | 20 | |  |  |  |  |  |  |  |  |  |  |  |  |
| Leroy Merlin | | 25 | | | 25 | |  |  |  |  |  |  |  |  |  |  |  |  |
| Lush | | 15 | | | 20 | |  |  |  |  |  |  |  |  |  |  |  |  |
| MediaWorld | | 15 | | | 15 | |  |  |  |  |  |  |  |  |  |  |  |  |
| Moisman Sport | | 25 | | | 25 | |  |  |  |  |  |  |  |  |  |  |  |  |
| Mondadori bookstore | | 30 | | | 30 | |  |  |  |  |  |  |  |  |  |  |  |  |
| Mondadori Sestri | | 30 | | | 30 | |  |  |  |  |  |  |  |  |  |  |  |  |
| Motivi | | 30 | | | 30 | |  |  |  |  |  |  |  |  |  |  |  |  |
| Nespresso boutique | | 15 | | | 20 | |  |  |  |  |  |  |  |  |  |  |  |  |
| OVS Brigata Liguria | | 25 | | | 20 | |  |  |  |  |  |  |  |  |  |  |  |  |
| OVS Fiumara | | 10 | | | 10 | |  |  |  |  |  |  |  |  |  |  |  |  |
| OVS Via Sestri | | 25 | | | 30 | |  |  |  |  |  |  |  |  |  |  |  |  |
| Pitta Rosso | | 10 | | | 10 | |  |  |  |  |  |  |  |  |  |  |  |  |
| Promoclub | | 20 | | | 25 | |  |  |  |  |  |  |  |  |  |  |  |  |
| Romanengo | | 15 | | | 20 | |  |  |  |  |  |  |  |  |  |  |  |  |
| Salewa | | 20 | | | 20 | |  |  |  |  |  |  |  |  |  |  |  |  |
| Stradivarius Fiumara | | 10 | | | 25 | |  |  |  |  |  |  |  |  |  |  |  |  |
| Supershop Brignole | | 20 | | | 20 | |  |  |  |  |  |  |  |  |  |  |  |  |
| Thun shop via XX Zsettembre | | 20 | | | 25 | |  |  |  |  |  |  |  |  |  |  |  |  |
| Tigotà | | 20 | | | 20 | |  |  |  |  |  |  |  |  |  |  |  |  |
| Toys centre | | 25 | | | 20 | |  |  |  |  |  |  |  |  |  |  |  |  |
| Triumph lingerie | | 25 | | | 25 | |  |  |  |  |  |  |  |  |  |  |  |  |
| Unieuro | | 20 | | | 25 | |  |  |  |  |  |  |  |  |  |  |  |  |
| Valigeria Sanson | | 20 | | | 20 | |  |  |  |  |  |  |  |  |  |  |  |  |
| Wheelup | | 25 | | | 20 | |  |  |  |  |  |  |  |  |  |  |  |  |
| Zuicki | | 10 | | | 10 | |  |  |  |  |  |  |  |  |  |  |  |  |

| **Descriptives** | | | **OBS 1** | | | **OBS 2** | |  | |  | |  | | |  | | |  | |  | | |  |  |  |
| --- | --- | --- | --- | --- | --- | --- | --- | --- | --- | --- | --- | --- | --- | --- | --- | --- | --- | --- | --- | --- | --- | --- | --- | --- | --- |
| Nbr. of observations | | | 44 | | | 44 | |  | |  | |  | | |  | | |  | |  | | |  |  |  |
| Nbr. of missing values | | | 0 | | | 0 | |  | |  | |  | | |  | | |  | |  | | |  |  |  |
| Obs. without missing data | | | 44 | | | 44 | |  | |  | |  | | |  | | |  | |  | | |  |  |  |
| Minimum | | | 10.000 | | | 10.000 | |  | |  | |  | | |  | | |  | |  | | |  |  |  |
| Maximum | | | 30.000 | | | 30.000 | |  | |  | |  | | |  | | |  | |  | | |  |  |  |
| Freq. of minimum | | | 6 | | | 3 | |  | |  | |  | | |  | | |  | |  | | |  |  |  |
| Freq. of maximum | | | 6 | | | 8 | |  | |  | |  | | |  | | |  | |  | | |  |  |  |
| Median | | | 20.000 | | | 20.000 | |  | |  | |  | | |  | | |  | |  | | |  |  |  |
| Mean | | | 20.909 | | | 22.159 | |  | |  | |  | | |  | | |  | |  | | |  |  |  |
| Variance (n-1) | | | 37.526 | | | 28.369 | |  | |  | |  | | |  | | |  | |  | | |  |  |  |
| Standard deviation (n-1) | | | 6.126 | | | 5.326 | |  | |  | |  | | |  | | |  | |  | | |  |  |  |
| Median absolute deviation | | | 5.000 | | | 5.000 | |  | |  | |  | | |  | | |  | |  | | |  |  |  |
|  | | |  | | |  | |  | |  | |  | | |  | | |  | |  | | |  |  |  |
| **Shapiro-Wilk test (OBS 1):** | | |  | | |  | |  | |  | |  | | |  | | |  | |  | | |  |  |  |
|  | | |  | | |  | |  | |  | |  | | |  | | |  | |  | | |  |  |  |
| W | | | 0.900 | | |  | |  | |  | |  | | |  | | |  | |  | | |  |  |  |
| p-value (Two-tailed) | | | **0.001** | | |  | |  | |  | |  | | |  | | |  | |  | | |  |  |  |
| alpha | | | 0.05 | | |  | |  | |  | |  | | |  | | |  | |  | | |  |  |  |
| Retail shops n=44*SEP*Summary statistics*SEP*$M$57 Retail shops n=44*SEP*Results of the resampling (X1)*SEP*$M$63 | | |  | | |  | |  | |  | |  | | |  | | |  | |  | | |  |  |  |
| Test interpretation: | | |  | | |  | |  | |  | |  | | |  | | |  | |  | | |  |  |  |
| H0: The variable from which the sample was extracted follows a Normal distribution. | | | | | | | | | | | | | | |  | | |  | |  | | |  |  |  |
| Ha: The variable from which the sample was extracted does not follow a Normal distribution. | | | | | | | | | | | | | | | | | |  | |  | | |  |  |  |
| As the computed p-value is lower than the significance level alpha=0.05,  one should reject the null hypothesis H0, and accept the alternative hypothesis Ha. | | | | | | | | | | | | | | | | | | | | | | |  |  |  |
|  |  |  |  |  |  |  |  |  |  |  |  |  |  |  |  |  |  |  |  |  |  |  |  |  |  |
|  | | |  | | |  | |  | |  | |  | | |  | | |  | |  | | |  |  |  |
| **Shapiro-Wilk test (OBS 2):** | |  | | | |  | |  | |  | |  | | |  | | |  | |  | | |  |  |  |
|  | |  | | | |  | |  | |  | |  | | |  | | |  | |  | | |  |  |  |
| W | | 0.871 | | | |  | |  | |  | |  | | |  | | |  | |  | | |  |  |  |
| p-value (Two-tailed) | | **0.000** | | | |  | |  | |  | |  | | |  | | |  | |  | | |  |  |  |
| alpha | | 0.05 | | | |  | |  | |  | |  | | |  | | |  | |  | | |  |  |  |
|  | |  | | | |  | |  | |  | |  | | |  | | |  | |  | | |  |  |  |
| Test interpretation: | |  | | | |  | |  | |  | |  | | |  | | |  | |  | | |  |  |  |
| H0: The variable from which the sample was extracted follows a Normal distribution. | | | | | | | | | | | | | | |  | | |  | |  | | |  |  |  |
| Ha: The variable from which the sample was extracted does not follow a Normal distribution. | | | | | | | | | | | | | | | | | |  | |  | | |  |  |  |
| As the computed p-value is lower than the significance level alpha=0.05,  one should reject the null hypothesis H0, and accept the alternative hypothesis Ha. | | | | | | | | | | | | | | | | | | | | | | |  |  |  |
|  |  |  |  |  |  |  |  |  |  |  |  |  |  |  |  |  |  |  |  |  |  |  |  |  |  |
|  | |  | | | |  | |  | |  | |  | | |  | | |  | |  | | |  |  |  |
| **Results of the resampling (OBS 1):** | | | | | |  | | | |  | | | | | | |  | | |  | | |  | |  |
|  | | |  | | |  | | | |  | | | | | | |  | | |  | | |  | |  |
| Parameters | | | Estimator | | | Estimator (Bootstrap) | | | | Standard deviation (Bootstrap) | | | | | | |  | | |  | | |  | |  |
| Mean  RunProcRSP Form118.txt CheckBoxTrans,CheckBox,0,False,03,False,Trans,False,,, ListBoxQuanti,ListBox,,True,200000000000_Outputs,True,,False,,, CheckBoxHist,CheckBox,0,True,300000000000_Charts,True,Histograms,False,,, OptionButtonHistBar,OptionButton,-1,True,300000000100_Charts,True,Bars,False,,, OptionButtonHistCont,OptionButton,0,True,300000010100_Charts,True,Continuous line,False,,, CheckBoxCum,CheckBox,0,True,300000000200_Charts,True,Cumulative histograms,False,,, OptionButtonHisBased,OptionButton,-1,True,300000000300_Charts,True,Based on the histogram,False,,, OptionButtonECDF,OptionButton,0,True,300000010300_Charts,True,Empirical cumulative distribution,False,,, CheckBoxRData,CheckBox,0,True,200000000008_Outputs,True,Resamples,False,,, TextBoxConfPer,TextBox,95,True,200000000003_Outputs,True,Confidence interval (%):,False,,, CheckBoxRStat,CheckBox,0,True,200000000007_Outputs,True,Resampled statistics,False,,, CheckBoxNormInt,CheckBox,0,True,200000000004_Outputs,True,Standard bootstrap interval,False,,, CheckBoxPercInt,CheckBox,0,True,200000000005_Outputs,True,Simple percentile interval,False,,, CheckBoxBiasInt,CheckBox,0,True,200000000006_Outputs,True,B.C. percentile interval,False,,, OptionButton_MVRemove,OptionButton,0,True,100000000100_Missing data,True,Remove the observations,False,,, OptionButton_MVEstimate,OptionButton,0,True,100000000300_Missing data,True,Estimate missing data,False,,, OptionButton_MeanMode,OptionButton,-1,True,100000000400_Missing data,True,Mean,False,,, OptionButtonAll,OptionButton,-1,True,100000000200_Missing data,True,For all samples,False,,, OptionButtonRestrict,OptionButton,0,True,100000010200_Missing data,True,For the corresponding sample,False,,, OptionButtonMVRefuse,OptionButton,-1,True,100000000000_Missing data,True,Do not accept missing data,False,,, OptionButton_W,OptionButton,0,True,000000000001_General,True,Workbook,False,,, OptionButton_R,OptionButton,-1,True,000000010001_General,True,Range,False,,, OptionButton_S,OptionButton,0,True,000000020001_General,True,Sheet,False,,, RefEdit_R,RefEdit0,'Sheet6'!$M$46,True,000000000101_General,True,Range:,False,,1,1 CheckBoxVarLabels,CheckBox,0,True,000000000201_General,True,Sample labels,False,,, CheckBox_W,CheckBox,0,True,000000000301_General,True,Weights,False,,, RefEdit_W,RefEdit,,True,000000000401_General,True,Weights:,False,,, TextBox_Resample,TextBox,1000,True,000000000000_General,True,Number of samples:,False,,, RefEdit_X,RefEdit0,'Sheet6'!$I$2:$I$45,True,000000000100_General,True,Quantitative data:,False,,44,1 ComboBoxMethod,ComboBox,0,True,000000010300_General,True,Method:,False,,, TextBox_obs,TextBox,5,True,000000000500_General,True,Sample size:,False,,, ComboBoxChartY,ComboBox,2,True,300000000101_Charts,True,Ordinate of the histograms:,False,,,  1 ListBox 118 ListBoxQuanti  1 22  Sum,0 Mean,-1 Variance (n),-1 Variance (n-1),-1 Standard deviation (n),-1 Standard deviation (n-1),-1 Median,-1 1st Quartile,0 3rd Quartile,0 Variation coefficient,0 Standard error of the mean,0 Mean absolute deviation,0 Median absolute deviation,-1 Geometric mean,0 Geometric standard deviation,0 Harmonic mean,0 1-Percentile,0 99-Percentile,0 2.5-Percentile,0 97.5-Percentile,0 5-Percentile,0 95-Percentile,0  328611  Retail shops n=44*SEP*Summary statistics*SEP*$M$57 Retail shops n=44*SEP*Results of the resampling (X1)*SEP*$M$63 | | | 20.909 | | | 20.898 | | | | 0.884 | | | | | | |  | | |  | | |  | |  |
| Variance (n) | | | 36.674 | | | 35.805 | | | | 6.212 | | | | | | |  | | |  | | |  | |  |
| Variance (n-1) | | | 37.526 | | | 36.637 | | | | 6.357 | | | | | | |  | | |  | | |  | |  |
| Standard deviation (n) | | | 6.056 | | | 5.961 | | | | 0.524 | | | | | | |  | | |  | | |  | |  |
| Standard deviation (n-1) | | | 6.126 | | | 6.030 | | | | 0.530 | | | | | | |  | | |  | | |  | |  |
| Median | | | 20.000 | | | 20.843 | | | | 1.732 | | | | | | |  | | |  | | |  | |  |
| Median absolute deviation | | | 5.000 | | | 4.763 | | | | 0.807 | | | | | | |  | | |  | | |  | |  |
|  | | |  | | |  | | | |  | | | | | | |  | | |  | | |  | |  |
| **Results of the resampling (OBS 2):** | | | |  | | |  | | | | | |  | |  | |  |  |  |  |  |  |  |  |  |
|  |  | | |  | | |  | | | | | |  | |  | |  |  |  |  |  |  |  |  |  |
| Parameters | Estimator | | | Estimator (Bootstrap) | | | Standard deviation (Bootstrap) | | | | | |  | |  | |  |  |  |  |  |  |  |  |  |
| Mean | 22.159 | | | 22.184 | | | 0.788 | | | | | |  | |  | |  |  |  |  |  |  |  |  |  |
| Variance (n) | 27.725 | | | 27.357 | | | 5.747 | | | | | |  | |  | |  |  |  |  |  |  |  |  |  |
| Variance (n-1) | 28.369 | | | 27.993 | | | 5.881 | | | | | |  | |  | |  |  |  |  |  |  |  |  |  |
| Standard deviation (n) | 5.265 | | | 5.202 | | | 0.548 | | | | | |  | |  | |  |  |  |  |  |  |  |  |  |
| Standard deviation (n-1) | 5.326 | | | 5.262 | | | 0.554 | | | | | |  | |  | |  |  |  |  |  |  |  |  |  |
| Median | 20.000 | | | 20.860 | | | 1.747 | | | | | |  | |  | |  |  |  |  |  |  |  |  |  |
| Median absolute deviation | 5.000 | | | 3.455 | | | 2.057 | | | | | |  | |  | |  |  |  |  |  |  |  |  |  |
|  |  | | |  | | |  | | | | | |  | |  | |  |  |  |  |  |  |  |  |  |
| **Mood test:** | |  | | |  | |  | |  | |  | | |  | |  | | |  | |  |  |  |  |  |
|  | |  | | |  | |  | |  | |  | | |  | |  | | |  | |  |  |  |  |  |
| U  RunProcRSP Form118.txt CheckBoxTrans,CheckBox,0,False,03,False,Trans,False,,, ListBoxQuanti,ListBox,,True,200000000000_Outputs,True,,False,,, CheckBoxHist,CheckBox,0,True,300000000000_Charts,True,Histograms,False,,, OptionButtonHistBar,OptionButton,-1,True,300000000100_Charts,True,Bars,False,,, OptionButtonHistCont,OptionButton,0,True,300000010100_Charts,True,Continuous line,False,,, CheckBoxCum,CheckBox,0,True,300000000200_Charts,True,Cumulative histograms,False,,, OptionButtonHisBased,OptionButton,-1,True,300000000300_Charts,True,Based on the histogram,False,,, OptionButtonECDF,OptionButton,0,True,300000010300_Charts,True,Empirical cumulative distribution,False,,, CheckBoxRData,CheckBox,0,True,200000000008_Outputs,True,Resamples,False,,, TextBoxConfPer,TextBox,95,True,200000000003_Outputs,True,Confidence interval (%):,False,,, CheckBoxRStat,CheckBox,0,True,200000000007_Outputs,True,Resampled statistics,False,,, CheckBoxNormInt,CheckBox,0,True,200000000004_Outputs,True,Standard bootstrap interval,False,,, CheckBoxPercInt,CheckBox,0,True,200000000005_Outputs,True,Simple percentile interval,False,,, CheckBoxBiasInt,CheckBox,0,True,200000000006_Outputs,True,B.C. percentile interval,False,,, OptionButton_MVRemove,OptionButton,0,True,100000000100_Missing data,True,Remove the observations,False,,, OptionButton_MVEstimate,OptionButton,0,True,100000000300_Missing data,True,Estimate missing data,False,,, OptionButton_MeanMode,OptionButton,-1,True,100000000400_Missing data,True,Mean,False,,, OptionButtonAll,OptionButton,-1,True,100000000200_Missing data,True,For all samples,False,,, OptionButtonRestrict,OptionButton,0,True,100000010200_Missing data,True,For the corresponding sample,False,,, OptionButtonMVRefuse,OptionButton,-1,True,100000000000_Missing data,True,Do not accept missing data,False,,, OptionButton_W,OptionButton,0,True,000000000001_General,True,Workbook,False,,, OptionButton_R,OptionButton,-1,True,000000010001_General,True,Range,False,,, OptionButton_S,OptionButton,0,True,000000020001_General,True,Sheet,False,,, RefEdit_R,RefEdit0,'Sheet6'!$M$74,True,000000000101_General,True,Range:,False,,0,0 CheckBoxVarLabels,CheckBox,0,True,000000000201_General,True,Sample labels,False,,, CheckBox_W,CheckBox,0,True,000000000301_General,True,Weights,False,,, RefEdit_W,RefEdit,,True,000000000401_General,True,Weights:,False,,, TextBox_Resample,TextBox,1000,True,000000000000_General,True,Number of samples:,False,,, RefEdit_X,RefEdit0,'Sheet6'!$J$2:$J$45,True,000000000100_General,True,Quantitative data:,False,,44,1 ComboBoxMethod,ComboBox,0,True,000000010300_General,True,Method:,False,,, TextBox_obs,TextBox,5,True,000000000500_General,True,Sample size:,False,,, ComboBoxChartY,ComboBox,2,True,300000000101_Charts,True,Ordinate of the histograms:,False,,,  1 ListBox 118 ListBoxQuanti  1 22  Sum,0 Mean,-1 Variance (n),-1 Variance (n-1),-1 Standard deviation (n),-1 Standard deviation (n-1),-1 Median,-1 1st Quartile,0 3rd Quartile,0 Variation coefficient,0 Standard error of the mean,0 Mean absolute deviation,0 Median absolute deviation,-1 Geometric mean,0 Geometric standard deviation,0 Harmonic mean,0 1-Percentile,0 99-Percentile,0 2.5-Percentile,0 97.5-Percentile,0 5-Percentile,0 95-Percentile,0  851734  Retail shops n=44*SEP*Summary statistics*SEP*$M$85 Retail shops n=44*SEP*Results of the resampling (X1)*SEP*$M$91 | | 0.046 | | |  | |  | |  | |  | | |  | |  | | |  | |  |  |  |  |  |
| Critical value | | 3.841 | | |  | |  | |  | |  | | |  | |  | | |  | |  |  |  |  |  |
| DF | | 1.000 | | |  | |  | |  | |  | | |  | |  | | |  | |  |  |  |  |  |
| p-value | | 0.823 | | |  | |  | |  | |  | | |  | |  | | |  | |  |  |  |  |  |
| alpha | | 0.05 | | |  | |  | |  | |  | | |  | |  | | |  | |  |  |  |  |  |
| The p-value has been computed using 10000 Monte Carlo simulations. Time elapsed: 0s. | | | | | | | | | | | | | | | |  | | |  | |  |  |  |  |  |
|  | |  | | |  | |  | |  | |  | | |  | |  | | |  | |  |  |  |  |  |
| Test interpretation: | |  | | |  | |  | |  | |  | | |  | |  | | |  | |  |  |  |  |  |
| H0: The medians of OBS 1 and OBS 2 are equal. | | | | | | |  | |  | |  | | |  | |  | | |  | |  |  |  |  |  |
| Ha: Medians of OBS 1 and OBS 2 are not equal | | | | | | |  | |  | |  | | |  | |  | | |  | |  |  |  |  |  |
| As the computed p-value is greater than the significance level alpha=0.05,  one cannot reject the null hypothesis H0. | | | | | | | | | | | | | | | | | | | | |  |  |  |  |  |
|  |  |  |  |  |  |  |  |  |  |  |  |  |  |  |  |  |  |  |  |  |  |  |  |  |  |

| **Post offices** | **OBS 1** | **OBS 2** |
| --- | --- | --- |
| Poste Corso Firenze | 10 | 20 |
| Poste Corso Martinetti | 20 | 15 |
| Poste Corso Martinetti | 20 | 15 |
| Poste Cso Europa 1068 | 15 | 15 |
| Poste Cso Europa 546 | 15 | 15 |
| Poste Cso Sardegna 2 | 15 | 15 |
| Poste Piazza Cavour | 15 | 15 |
| Poste Piazza Gaggero | 25 | 20 |
| Poste Piazza Monastero | 15 | 15 |
| Poste Piazza Rovere | 25 | 25 |
| Poste Piazzale Marassi | 25 | 20 |
| Poste Piazzale Rizzolio | 25 | 15 |
| Poste Piazzale Sigelli | 15 | 15 |
| Poste Via Airaghi | 20 | 20 |
| Poste Via Assarotti | 20 | 20 |
| Poste Via Blelè | 15 | 15 |
| Poste Via Borzoli | 15 | 15 |
| Poste Via Caldesi | 20 | 15 |
| Poste Via Canevari | 15 | 15 |
| Poste Via Cantore | 20 | 20 |
| Poste Via Carbone | 20 | 20 |
| Poste Via Catalani | 25 | 25 |
| Poste Via Colombo | 20 | 20 |
| Poste Via da Pozzo | 20 | 20 |
| Poste Via dal Canto | 20 | 20 |
| Poste Via Dante | 15 | 20 |
| Poste Via Donghi | 15 | 15 |
| Poste Via Fereggiano | 20 | 20 |
| Poste Via Franchini | 20 | 20 |
| Poste Via Francia | 15 | 25 |
| Poste Via Giannelli | 15 | 20 |
| Poste Via Granello | 20 | 25 |
| Poste Via Ilva | 15 | 20 |
| Poste Via Langustena | 15 | 15 |
| Poste Via Martiri della Libertà | 20 | 20 |
| Poste Via Molassana | 15 | 20 |
| Poste Via Multedo | 15 | 15 |
| Poste Via Napoli | 25 | 25 |
| Poste Via Olivieri | 15 | 15 |
| Poste Via Orsini | 20 | 20 |
| Poste Via Pastorino | 20 | 20 |
| Poste Via Piacenza | 15 | 15 |
| Poste Via Piccone | 25 | 15 |
| Poste Via Pisa | 15 | 15 |
| Poste Via Pozzo | 20 | 20 |
| Poste Via Re di Puglia | 20 | 25 |
| Poste Via Robino | 20 | 15 |
| Poste Via Roggerone | 20 | 20 |
| Poste Via S Franceco de Paola | 25 | 15 |
| Poste Via San Fruttuoso | 15 | 25 |
| Poste Via San Quirico | 15 | 20 |
| Poste Via San Romolo | 15 | 15 |
| Poste Via Schiaffino | 25 | 25 |
| Poste Via Spalato | 15 | 15 |
| Poste Via Struppa | 15 | 15 |
| Poste Via Terpi | 15 | 20 |
| Poste Via Toscanelli | 25 | 20 |
| Poste Via Ulanowski | 20 | 20 |
| Poste Via Valle Chiara | 15 | 15 |
| Poste Viale Cembrano | 15 | 10 |

| **Descriptives** | **OBS 1** | **OBS 2** |  |  |  |  |  |  |  |
| --- | --- | --- | --- | --- | --- | --- | --- | --- | --- |
| Nbr. of observations | 60 | 60 |  |  |  |  |  |  |  |
| Nbr. of missing values | 0 | 0 |  |  |  |  |  |  |  |
| Obs. without missing data | 60 | 60 |  |  |  |  |  |  |  |
| Minimum | 10.000 | 10.000 |  |  |  |  |  |  |  |
| Maximum | 25.000 | 25.000 |  |  |  |  |  |  |  |
| Freq. of minimum | 1 | 1 |  |  |  |  |  |  |  |
| Freq. of maximum | 10 | 8 |  |  |  |  |  |  |  |
| Median | 20.000 | 20.000 |  |  |  |  |  |  |  |
| Mean | 18.333 | 18.333 |  |  |  |  |  |  |  |
| Variance (n-1) | 14.972 | 13.277 |  |  |  |  |  |  |  |
| Standard deviation (n-1) | 3.869 | 3.644 |  |  |  |  |  |  |  |
| Median absolute deviation | 5.000 | 5.000 |  |  |  |  |  |  |  |
|  |  |  |  |  |  |  |  |  |  |
| **Shapiro-Wilk test (OBS 1):** | |  |  |  |  |  |  |  |  |
|  |  |  |  |  |  |  |  |  |  |
| W | 0.811 |  |  |  |  |  |  |  |  |
| p-value (Two-tailed) | **<0.0001** |  |  |  |  |  |  |  |  |
| alpha | 0.05 |  |  |  |  |  |  |  |  |
| Post offices n=60*SEP*Summary statistics*SEP*$M$73 Post offices n=60*SEP*Results of the resampling (X1)*SEP*$M$79 |  |  |  |  |  |  |  |  |  |
| Test interpretation: |  |  |  |  |  |  |  |  |  |
| H0: The variable from which the sample was extracted follows a Normal distribution. | | | | | | |  |  |  |
| Ha: The variable from which the sample was extracted does not follow a Normal distribution. | | | | | | |  |  |  |
| As the computed p-value is lower than the significance level alpha=0.05,  one should reject the null hypothesis H0, and accept the alternative hypothesis Ha. | | | | | | | | |  |
|  |  |  |  |  |  |  |  |  |  |
|  |  |  |  |  |  |  |  |  |  |

| **Shapiro-Wilk test (OBS 2):** | | | |  |  |  |  |  |  |
| --- | --- | --- | --- | --- | --- | --- | --- | --- | --- |
|  |  |  |  |  |  |  |  |  |  |
| W | 0.818 |  |  |  |  |  |  |  |  |
| p-value (Two-tailed) | **<0.0001** |  |  |  |  |  |  |  |  |
| alpha | 0.05 |  |  |  |  |  |  |  |  |
|  |  |  |  |  |  |  |  |  |  |
| Test interpretation: | |  |  |  |  |  |  |  |  |
| H0: The variable from which the sample was extracted follows a Normal distribution. | | | | | | | | |  |
| Ha: The variable from which the sample was extracted does not follow a Normal distribution. | | | | | | | | | |
| As the computed p-value is lower than the significance level alpha=0.05, one should reject the null hypothesis H0, and accept the alternative hypothesis Ha. | | | | | | | | |  |
|  |  |  |  |  |  |  |  |  |  |

| **Results of the resampling (OBS 1):** | | | | | | | |  | | | | | | | |  | |  | |  | | |  |  |  |
| --- | --- | --- | --- | --- | --- | --- | --- | --- | --- | --- | --- | --- | --- | --- | --- | --- | --- | --- | --- | --- | --- | --- | --- | --- | --- |
|  |  | | |  | | | |  | | | | | | | |  | |  | |  | | |  |  |  |
| Parameters | Estimator | | | Estimator (Bootstrap) | | | | Standard deviation (Bootstrap) | | | | | | | |  | |  | |  | | |  |  |  |
| Mean | 18.333 | | | 18.351 | | | | 0.489 | | | | | | | |  | |  | |  | | |  |  |  |
| Variance (n) | 14.722 | | | 14.507 | | | | 2.066 | | | | | | | |  | |  | |  | | |  |  |  |
| Variance (n-1) | 14.972 | | | 14.753 | | | | 2.101 | | | | | | | |  | |  | |  | | |  |  |  |
| Standard deviation (n) | 3.837 | | | 3.799 | | | | 0.272 | | | | | | | |  | |  | |  | | |  |  |  |
| Standard deviation (n-1) | 3.869 | | | 3.831 | | | | 0.275 | | | | | | | |  | |  | |  | | |  |  |  |
| Median | 20.000 | | | 18.088 | | | | 2.295 | | | | | | | |  | |  | |  | | |  |  |  |
| Median absolute deviation | 5.000 | | | 3.340 | | | | 2.136 | | | | | | | |  | |  | |  | | |  |  |  |
|  |  | | |  | | | |  | | | | | | | |  | |  | |  | | |  |  |  |
| **Results of the resampling (OBS 2):** | | | | | | | | | | | | | |  | | |  | |  | | |  |  |  |  |
|  |  |  | | | | |  | | | | | |  |  | | |  | |  | | |  |  |  |  |
| Parameters | Estimator | Estimator (Bootstrap) | | | | | Standard deviation (Bootstrap) | | | | | |  |  | | |  | |  | | |  |  |  |  |
| Mean | 18.333 | 18.334 | | | | | 0.479 | | | | | |  |  | | |  | |  | | |  |  |  |  |
| Variance (n) | 13.056 | 12.824 | | | | | 1.964 | | | | | |  |  | | |  | |  | | |  |  |  |  |
| Variance (n-1) | 13.277 | 13.042 | | | | | 1.998 | | | | | |  |  | | |  | |  | | |  |  |  |  |
| Standard deviation (n) | 3.613 | 3.571 | | | | | 0.273 | | | | | |  |  | | |  | |  | | |  |  |  |  |
| Standard deviation (n-1) | 3.644 | 3.601 | | | | | 0.275 | | | | | |  |  | | |  | |  | | |  |  |  |  |
| Median | 20.000 | 18.945 | | | | | 1.941 | | | | | |  |  | | |  | |  | | |  |  |  |  |
| Median absolute deviation | 5.000 | 3.643 | | | | | 2.014 | | | | | |  |  | | |  | |  | | |  |  |  |  |
|  |  |  | | | | |  | | | | | |  |  | | |  | |  | | |  |  |  |  |
| **Mood test:** | | |  | | |  | | | |  | |  | | |  | | |  | | |  | | |  |  |
|  | | |  | | |  | | | |  | |  | | |  | | |  | | |  | | |  |  |
| U  RunProcRSP Form118.txt CheckBoxTrans,CheckBox,0,False,03,False,Trans,False,,, ListBoxQuanti,ListBox,,True,200000000000_Outputs,True,,False,,, CheckBoxHist,CheckBox,0,True,300000000000_Charts,True,Histograms,False,,, OptionButtonHistBar,OptionButton,-1,True,300000000100_Charts,True,Bars,False,,, OptionButtonHistCont,OptionButton,0,True,300000010100_Charts,True,Continuous line,False,,, CheckBoxCum,CheckBox,0,True,300000000200_Charts,True,Cumulative histograms,False,,, OptionButtonHisBased,OptionButton,-1,True,300000000300_Charts,True,Based on the histogram,False,,, OptionButtonECDF,OptionButton,0,True,300000010300_Charts,True,Empirical cumulative distribution,False,,, CheckBoxRData,CheckBox,0,True,200000000008_Outputs,True,Resamples,False,,, TextBoxConfPer,TextBox,95,True,200000000003_Outputs,True,Confidence interval (%):,False,,, CheckBoxRStat,CheckBox,0,True,200000000007_Outputs,True,Resampled statistics,False,,, CheckBoxNormInt,CheckBox,0,True,200000000004_Outputs,True,Standard bootstrap interval,False,,, CheckBoxPercInt,CheckBox,0,True,200000000005_Outputs,True,Simple percentile interval,False,,, CheckBoxBiasInt,CheckBox,0,True,200000000006_Outputs,True,B.C. percentile interval,False,,, OptionButton_MVRemove,OptionButton,0,True,100000000100_Missing data,True,Remove the observations,False,,, OptionButton_MVEstimate,OptionButton,0,True,100000000300_Missing data,True,Estimate missing data,False,,, OptionButton_MeanMode,OptionButton,-1,True,100000000400_Missing data,True,Mean,False,,, OptionButtonAll,OptionButton,-1,True,100000000200_Missing data,True,For all samples,False,,, OptionButtonRestrict,OptionButton,0,True,100000010200_Missing data,True,For the corresponding sample,False,,, OptionButtonMVRefuse,OptionButton,-1,True,100000000000_Missing data,True,Do not accept missing data,False,,, OptionButton_W,OptionButton,0,True,000000000001_General,True,Workbook,False,,, OptionButton_R,OptionButton,-1,True,000000010001_General,True,Range,False,,, OptionButton_S,OptionButton,0,True,000000020001_General,True,Sheet,False,,, RefEdit_R,RefEdit0,'Sheet7'!$M$90,True,000000000101_General,True,Range:,False,,0,0 CheckBoxVarLabels,CheckBox,0,True,000000000201_General,True,Sample labels,False,,, CheckBox_W,CheckBox,0,True,000000000301_General,True,Weights,False,,, RefEdit_W,RefEdit,,True,000000000401_General,True,Weights:,False,,, TextBox_Resample,TextBox,1000,True,000000000000_General,True,Number of samples:,False,,, RefEdit_X,RefEdit0,'Sheet7'!$J$2:$J$61,True,000000000100_General,True,Quantitative data:,False,,60,1 ComboBoxMethod,ComboBox,0,True,000000010300_General,True,Method:,False,,, TextBox_obs,TextBox,5,True,000000000500_General,True,Sample size:,False,,, ComboBoxChartY,ComboBox,2,True,300000000101_Charts,True,Ordinate of the histograms:,False,,,  1 ListBox 118 ListBoxQuanti  1 22  Sum,0 Mean,-1 Variance (n),-1 Variance (n-1),-1 Standard deviation (n),-1 Standard deviation (n-1),-1 Median,-1 1st Quartile,0 3rd Quartile,0 Variation coefficient,0 Standard error of the mean,0 Mean absolute deviation,0 Median absolute deviation,-1 Geometric mean,0 Geometric standard deviation,0 Harmonic mean,0 1-Percentile,0 99-Percentile,0 2.5-Percentile,0 97.5-Percentile,0 5-Percentile,0 95-Percentile,0  244960  Post offices n=60*SEP*Summary statistics*SEP*$M$101 Post offices n=60*SEP*Results of the resampling (X1)*SEP*$M$107 | | | 0.065 | | |  | | | |  | |  | | |  | | |  | | |  | | |  |  |
| Critical value | | | 3.841 | | |  | | | |  | |  | | |  | | |  | | |  | | |  |  |
| DF | | | 1.000 | | |  | | | |  | |  | | |  | | |  | | |  | | |  |  |
| p-value | | | 0.798 | | |  | | | |  | |  | | |  | | |  | | |  | | |  |  |
| alpha | | | 0.05 | | |  | | | |  | |  | | |  | | |  | | |  | | |  |  |
| The p-value has been computed using 10000 Monte Carlo simulations. Time elapsed: 0s. | | | | | | | | | | | | | | | | | | | | |  | | |  |  |
|  | | |  | | |  | | | |  | |  | | |  | | |  | | |  | | |  |  |
| Test interpretation: | | |  | | |  | | | |  | |  | | |  | | |  | | |  | | |  |  |
| H0: The medians of OBS 1 and OBS 2 are equal. | | | | | | | | | |  | |  | | |  | | |  | | |  | | |  |  |
| Ha: Medians of OBS 1 and OBS 2 are not equal | | | | | | | | | |  | |  | | |  | | |  | | |  | | |  |  |
| As the computed p-value is greater than the significance level alpha=0.05,  RunProcRSP Form118.txt CheckBoxTrans,CheckBox,0,False,03,False,Trans,False,,, ListBoxQuanti,ListBox,,True,200000000000_Outputs,True,,False,,, CheckBoxHist,CheckBox,0,True,300000000000_Charts,True,Histograms,False,,, OptionButtonHistBar,OptionButton,-1,True,300000000100_Charts,True,Bars,False,,, OptionButtonHistCont,OptionButton,0,True,300000010100_Charts,True,Continuous line,False,,, CheckBoxCum,CheckBox,0,True,300000000200_Charts,True,Cumulative histograms,False,,, OptionButtonHisBased,OptionButton,-1,True,300000000300_Charts,True,Based on the histogram,False,,, OptionButtonECDF,OptionButton,0,True,300000010300_Charts,True,Empirical cumulative distribution,False,,, CheckBoxRData,CheckBox,0,True,200000000008_Outputs,True,Resamples,False,,, TextBoxConfPer,TextBox,95,True,200000000003_Outputs,True,Confidence interval (%):,False,,, CheckBoxRStat,CheckBox,0,True,200000000007_Outputs,True,Resampled statistics,False,,, CheckBoxNormInt,CheckBox,0,True,200000000004_Outputs,True,Standard bootstrap interval,False,,, CheckBoxPercInt,CheckBox,0,True,200000000005_Outputs,True,Simple percentile interval,False,,, CheckBoxBiasInt,CheckBox,0,True,200000000006_Outputs,True,B.C. percentile interval,False,,, OptionButton_MVRemove,OptionButton,0,True,100000000100_Missing data,True,Remove the observations,False,,, OptionButton_MVEstimate,OptionButton,0,True,100000000300_Missing data,True,Estimate missing data,False,,, OptionButton_MeanMode,OptionButton,-1,True,100000000400_Missing data,True,Mean,False,,, OptionButtonAll,OptionButton,-1,True,100000000200_Missing data,True,For all samples,False,,, OptionButtonRestrict,OptionButton,0,True,100000010200_Missing data,True,For the corresponding sample,False,,, OptionButtonMVRefuse,OptionButton,-1,True,100000000000_Missing data,True,Do not accept missing data,False,,, OptionButton_W,OptionButton,0,True,000000000001_General,True,Workbook,False,,, OptionButton_R,OptionButton,-1,True,000000010001_General,True,Range,False,,, OptionButton_S,OptionButton,0,True,000000020001_General,True,Sheet,False,,, RefEdit_R,RefEdit0,'Sheet7'!$M$118,True,000000000101_General,True,Range:,False,,0,0 CheckBoxVarLabels,CheckBox,0,True,000000000201_General,True,Sample labels,False,,, CheckBox_W,CheckBox,0,True,000000000301_General,True,Weights,False,,, RefEdit_W,RefEdit,,True,000000000401_General,True,Weights:,False,,, TextBox_Resample,TextBox,1000,True,000000000000_General,True,Number of samples:,False,,, RefEdit_X,RefEdit0,'Sheet7'!$K$2:$K$61,True,000000000100_General,True,Quantitative data:,False,,60,1 ComboBoxMethod,ComboBox,0,True,000000010300_General,True,Method:,False,,, TextBox_obs,TextBox,5,True,000000000500_General,True,Sample size:,False,,, ComboBoxChartY,ComboBox,2,True,300000000101_Charts,True,Ordinate of the histograms:,False,,,  1 ListBox 118 ListBoxQuanti  1 22  Sum,0 Mean,-1 Variance (n),-1 Variance (n-1),-1 Standard deviation (n),-1 Standard deviation (n-1),-1 Median,-1 1st Quartile,0 3rd Quartile,0 Variation coefficient,0 Standard error of the mean,0 Mean absolute deviation,0 Median absolute deviation,-1 Geometric mean,0 Geometric standard deviation,0 Harmonic mean,0 1-Percentile,0 99-Percentile,0 2.5-Percentile,0 97.5-Percentile,0 5-Percentile,0 95-Percentile,0  397599  Post offices n=60*SEP*Summary statistics*SEP*$M$129 Post offices n=60*SEP*Results of the resampling (X1)*SEP*$M$135  one cannot reject the null hypothesis H0. | | | | | | | | | | | | | | | | | | | | | | | | |  |
|  |  |  |  |  |  |  |  |  |  |  |  |  |  |  |  |  |  |  |  |  |  |  |  |  |  |
| **Banks** | | | | | **OBS 1** | | | | **OBS 2** | |  |  |  |  |  |  |  |  |  |  |  |  |  |  |  |
| Banca CARIGE Via Corsica | | | | | 15 | | | | 15 | |  |  |  |  |  |  |  |  |  |  |  |  |  |  |  |
| Banca di Sondrio Pzza Tomasseo | | | | | 20 | | | | 15 | |  |  |  |  |  |  |  |  |  |  |  |  |  |  |  |
| Banca di Sondrio Via XXV Aprile | | | | | 20 | | | | 20 | |  |  |  |  |  |  |  |  |  |  |  |  |  |  |  |
| Banca Italia Via Dante | | | | | 20 | | | | 20 | |  |  |  |  |  |  |  |  |  |  |  |  |  |  |  |
| Banco di Sardegna Pzza Fontane Marose | | | | | 20 | | | | 20 | |  |  |  |  |  |  |  |  |  |  |  |  |  |  |  |
| BNL Corso Torino | | | | | 15 | | | | 15 | |  |  |  |  |  |  |  |  |  |  |  |  |  |  |  |
| BNL Piazza Montano | | | | | 15 | | | | 15 | |  |  |  |  |  |  |  |  |  |  |  |  |  |  |  |
| BNL Via Bolzaneto | | | | | 15 | | | | 10 | |  |  |  |  |  |  |  |  |  |  |  |  |  |  |  |
| BNL Via Brigata Liguria | | | | | 15 | | | | 15 | |  |  |  |  |  |  |  |  |  |  |  |  |  |  |  |
| BNL Via Caprera | | | | | 10 | | | | 10 | |  |  |  |  |  |  |  |  |  |  |  |  |  |  |  |
| BNL Via Catalani | | | | | 15 | | | | 10 | |  |  |  |  |  |  |  |  |  |  |  |  |  |  |  |
| BNL Via Gherzi | | | | | 20 | | | | 10 | |  |  |  |  |  |  |  |  |  |  |  |  |  |  |  |
| BNL Via Jori | | | | | 15 | | | | 15 | |  |  |  |  |  |  |  |  |  |  |  |  |  |  |  |
| BNL Via Martiri della Libertà | | | | | 15 | | | | 15 | |  |  |  |  |  |  |  |  |  |  |  |  |  |  |  |
| BNL Via Murcarolo | | | | | 15 | | | | 15 | |  |  |  |  |  |  |  |  |  |  |  |  |  |  |  |
| BNL Via Torti | | | | | 15 | | | | 15 | |  |  |  |  |  |  |  |  |  |  |  |  |  |  |  |
| BPER Banca Viale Brigate Bisagno | | | | | 20 | | | | 10 | |  |  |  |  |  |  |  |  |  |  |  |  |  |  |  |
| Carige al Monte di Pietà | | | | | 25 | | | | 30 | |  |  |  |  |  |  |  |  |  |  |  |  |  |  |  |
| Carige Piazza delle Americhe | | | | | 15 | | | | 15 | |  |  |  |  |  |  |  |  |  |  |  |  |  |  |  |
| Carige Via Avio | | | | | 10 | | | | 10 | |  |  |  |  |  |  |  |  |  |  |  |  |  |  |  |
| Carige Via Cassa di Risparmio | | | | | 15 | | | | 15 | |  |  |  |  |  |  |  |  |  |  |  |  |  |  |  |
| Carige Via Cesarea | | | | | 25 | | | | 20 | |  |  |  |  |  |  |  |  |  |  |  |  |  |  |  |
| Carige Via Torti | | | | | 10 | | | | 20 | |  |  |  |  |  |  |  |  |  |  |  |  |  |  |  |
| Credem Piazza Dante | | | | | 20 | | | | 20 | |  |  |  |  |  |  |  |  |  |  |  |  |  |  |  |
| Intesa Cso Sardegna | | | | | 15 | | | | 10 | |  |  |  |  |  |  |  |  |  |  |  |  |  |  |  |
| Intesa Pzza Fontane Marose | | | | | 15 | | | | 15 | |  |  |  |  |  |  |  |  |  |  |  |  |  |  |  |
| Intesa Via Fieschi | | | | | 15 | | | | 15 | |  |  |  |  |  |  |  |  |  |  |  |  |  |  |  |
| Intesa Via Righetti | | | | | 10 | | | | 15 | |  |  |  |  |  |  |  |  |  |  |  |  |  |  |  |
| Intesa Via Rolando | | | | | 15 | | | | 15 | |  |  |  |  |  |  |  |  |  |  |  |  |  |  |  |
| Intesa Via Timavo | | | | | 15 | | | | 10 | |  |  |  |  |  |  |  |  |  |  |  |  |  |  |  |
| MPS Corso Sardegna | | | | | 10 | | | | 15 | |  |  |  |  |  |  |  |  |  |  |  |  |  |  |  |
| Unicredit Via Cornigliano | | | | | 15 | | | | 15 | |  |  |  |  |  |  |  |  |  |  |  |  |  |  |  |
| Unicredit Via Fiasella | | | | | 15 | | | | 15 | |  |  |  |  |  |  |  |  |  |  |  |  |  |  |  |
| Unicredit Via Garibaldi | | | | | 20 | | | | 20 | |  |  |  |  |  |  |  |  |  |  |  |  |  |  |  |
| Unicredit Via Gastaldi | | | | | 10 | | | | 10 | |  |  |  |  |  |  |  |  |  |  |  |  |  |  |  |
| Unicredit Via Sestri | | | | | 10 | | | | 10 | |  |  |  |  |  |  |  |  |  |  |  |  |  |  |  |
| Unicredit Via Vittorio Veneto | | | | | 15 | | | | 20 | |  |  |  |  |  |  |  |  |  |  |  |  |  |  |  |

| **Descriptives** | **OBS 1** | **OBS 2** |  |  |  |  |  |  |  |
| --- | --- | --- | --- | --- | --- | --- | --- | --- | --- |
| Nbr. of observations | 37 | 37 |  |  |  |  |  |  |  |
| Nbr. of missing values | 0 | 0 |  |  |  |  |  |  |  |
| Obs. without missing data | 37 | 37 |  |  |  |  |  |  |  |
| Minimum | 10.000 | 10.000 |  |  |  |  |  |  |  |
| Maximum | 25.000 | 30.000 |  |  |  |  |  |  |  |
| Freq. of minimum | 7 | 10 |  |  |  |  |  |  |  |
| Freq. of maximum | 2 | 1 |  |  |  |  |  |  |  |
| Median | 15.000 | 15.000 |  |  |  |  |  |  |  |
| Mean | 15.676 | 15.135 |  |  |  |  |  |  |  |
| Variance (n-1) | 15.503 | 18.731 |  |  |  |  |  |  |  |
| Standard deviation (n-1) | 3.937 | 4.328 |  |  |  |  |  |  |  |
| Median absolute deviation | 0.000 | 5.000 |  |  |  |  |  |  |  |
|  |  |  |  |  |  |  |  |  |  |
|  |  |  |  |  |  |  |  |  |  |
| **Shapiro-Wilk test (OBS 1):** |  |  |  |  |  |  |  |  |  |
|  |  |  |  |  |  |  |  |  |  |
| W | 0.843 |  |  |  |  |  |  |  |  |
| p-value (Two-tailed) | **0.000** |  |  |  |  |  |  |  |  |
| alpha  Banks n=37*SEP*Summary statistics*SEP*$M$50 Banks n=37*SEP*Results of the resampling (X1)*SEP*$M$56 | 0.05 |  |  |  |  |  |  |  |  |
|  |  |  |  |  |  |  |  |  |  |
| Test interpretation: |  |  |  |  |  |  |  |  |  |
| H0: The variable from which the sample was extracted follows a Normal distribution. | | | | | |  |  |  |  |
| Ha: The variable from which the sample was extracted does not follow a Normal distribution. | | | | | | |  |  |  |
| As the computed p-value is lower than the significance level alpha=0.05,  one should reject the null hypothesis H0, and accept the alternative hypothesis Ha. | | | | | | | | |  |
|  |  |  |  |  |  |  |  |  |  |

| **Shapiro-Wilk test (OBS 2):** | | | |  | |  |  | |  |  |  | |  |  |  |
| --- | --- | --- | --- | --- | --- | --- | --- | --- | --- | --- | --- | --- | --- | --- | --- |
|  |  |  |  |  | |  |  | |  |  |  | |  |  |  |
| W | 0.818 |  |  |  | |  |  | |  |  |  | |  |  |  |
| p-value (Two-tailed) | **<0.0001** |  |  |  | |  |  | |  |  |  | |  |  |  |
| alpha | 0.05 |  |  |  | |  |  | |  |  |  | |  |  |  |
|  |  |  |  |  | |  |  | |  |  |  | |  |  |  |
| Test interpretation: | |  |  |  | |  |  | |  |  |  | |  |  |  |
| H0: The variable from which the sample was extracted follows a Normal distribution. | | | | | | | | | | |  | |  |  |  |
| Ha: The variable from which the sample was extracted does not follow a Normal distribution. | | | | | | | | | | | | |  |  |  |
| As the computed p-value is lower than the significance level alpha=0.05, one should reject the null hypothesis H0, and accept the alternative hypothesis Ha. | | | | | | | | | | |  | |  |  |  |
|  |  |  |  |  |  |  |  |  |  |  |  | |  |  |  |
| **Results of the resampling (OBS 1):** | | | | |  | | |  | | | |  | |  |  |
| RunProcRSP Form118.txt CheckBoxTrans,CheckBox,0,False,03,False,Trans,False,,, ListBoxQuanti,ListBox,,True,200000000000_Outputs,True,,False,,, CheckBoxHist,CheckBox,0,True,300000000000_Charts,True,Histograms,False,,, OptionButtonHistBar,OptionButton,-1,True,300000000100_Charts,True,Bars,False,,, OptionButtonHistCont,OptionButton,0,True,300000010100_Charts,True,Continuous line,False,,, CheckBoxCum,CheckBox,0,True,300000000200_Charts,True,Cumulative histograms,False,,, OptionButtonHisBased,OptionButton,-1,True,300000000300_Charts,True,Based on the histogram,False,,, OptionButtonECDF,OptionButton,0,True,300000010300_Charts,True,Empirical cumulative distribution,False,,, CheckBoxRData,CheckBox,0,True,200000000008_Outputs,True,Resamples,False,,, TextBoxConfPer,TextBox,95,True,200000000003_Outputs,True,Confidence interval (%):,False,,, CheckBoxRStat,CheckBox,0,True,200000000007_Outputs,True,Resampled statistics,False,,, CheckBoxNormInt,CheckBox,0,True,200000000004_Outputs,True,Standard bootstrap interval,False,,, CheckBoxPercInt,CheckBox,0,True,200000000005_Outputs,True,Simple percentile interval,False,,, CheckBoxBiasInt,CheckBox,0,True,200000000006_Outputs,True,B.C. percentile interval,False,,, OptionButton_MVRemove,OptionButton,0,True,100000000100_Missing data,True,Remove the observations,False,,, OptionButton_MVEstimate,OptionButton,0,True,100000000300_Missing data,True,Estimate missing data,False,,, OptionButton_MeanMode,OptionButton,-1,True,100000000400_Missing data,True,Mean,False,,, OptionButtonAll,OptionButton,-1,True,100000000200_Missing data,True,For all samples,False,,, OptionButtonRestrict,OptionButton,0,True,100000010200_Missing data,True,For the corresponding sample,False,,, OptionButtonMVRefuse,OptionButton,-1,True,100000000000_Missing data,True,Do not accept missing data,False,,, OptionButton_W,OptionButton,0,True,000000000001_General,True,Workbook,False,,, OptionButton_R,OptionButton,-1,True,000000010001_General,True,Range,False,,, OptionButton_S,OptionButton,0,True,000000020001_General,True,Sheet,False,,, RefEdit_R,RefEdit0,'Sheet8'!$M$39,True,000000000101_General,True,Range:,False,,1,1 CheckBoxVarLabels,CheckBox,0,True,000000000201_General,True,Sample labels,False,,, CheckBox_W,CheckBox,0,True,000000000301_General,True,Weights,False,,, RefEdit_W,RefEdit,,True,000000000401_General,True,Weights:,False,,, TextBox_Resample,TextBox,1000,True,000000000000_General,True,Number of samples:,False,,, RefEdit_X,RefEdit0,'Sheet8'!$I$2:$I$38,True,000000000100_General,True,Quantitative data:,False,,37,1 ComboBoxMethod,ComboBox,0,True,000000010300_General,True,Method:,False,,, TextBox_obs,TextBox,5,True,000000000500_General,True,Sample size:,False,,, ComboBoxChartY,ComboBox,2,True,300000000101_Charts,True,Ordinate of the histograms:,False,,,  1 ListBox 118 ListBoxQuanti  1 22  Sum,0 Mean,-1 Variance (n),-1 Variance (n-1),-1 Standard deviation (n),-1 Standard deviation (n-1),-1 Median,-1 1st Quartile,0 3rd Quartile,0 Variation coefficient,0 Standard error of the mean,0 Mean absolute deviation,0 Median absolute deviation,-1 Geometric mean,0 Geometric standard deviation,0 Harmonic mean,0 1-Percentile,0 99-Percentile,0 2.5-Percentile,0 97.5-Percentile,0 5-Percentile,0 95-Percentile,0  218861  Banks n=37*SEP*Summary statistics*SEP*$M$50 Banks n=37*SEP*Results of the resampling (X1)*SEP*$M$56 | |  | | |  | | |  | | | |  | |  |  |
| Parameters | | Estimator | | | Estimator (Bootstrap) | | | Standard deviation (Bootstrap) | | | |  | |  |  |
| Mean | | 15.676 | | | 15.645 | | | 0.618 | | | |  | |  |  |
| Variance (n) | | 15.084 | | | 14.503 | | | 3.454 | | | |  | |  |  |
| Variance (n-1) | | 15.503 | | | 14.906 | | | 3.550 | | | |  | |  |  |
| Standard deviation (n) | | 3.884 | | | 3.781 | | | 0.458 | | | |  | |  |  |
| Standard deviation (n-1) | | 3.937 | | | 3.833 | | | 0.465 | | | |  | |  |  |
| Median | | 15.000 | | | 15.000 | | | 0.000 | | | |  | |  |  |
| Median absolute deviation | | 0.000 | | | 1.515 | | | 2.299 | | | |  | |  |  |
|  | |  | | |  | | |  | | | |  | |  |  |

| **Results of the resampling (OBS 2):** | | | | | | | | | | | | |  |  |  |  |  |  |
| --- | --- | --- | --- | --- | --- | --- | --- | --- | --- | --- | --- | --- | --- | --- | --- | --- | --- | --- |
|  |  |  | | | | |  | | | |  | |  |  |  |  |  |  |
| Parameters | Estimator | Estimator (Bootstrap) | | | | | Standard deviation (Bootstrap) | | | |  | |  |  |  |  |  |  |
| Mean | 15.135 | 15.140 | | | | | 0.699 | | | |  | |  |  |  |  |  |  |
| Variance (n) | 18.225 | 17.630 | | | | | 5.886 | | | |  | |  |  |  |  |  |  |
| Variance (n-1) | 18.731 | 18.119 | | | | | 6.050 | | | |  | |  |  |  |  |  |  |
| Standard deviation (n) | 4.269 | 4.142 | | | | | 0.688 | | | |  | |  |  |  |  |  |  |
| Standard deviation (n-1) | 4.328 | 4.199 | | | | | 0.697 | | | |  | |  |  |  |  |  |  |
| Median | 15.000 | 14.990 | | | | | 0.316 | | | |  | |  |  |  |  |  |  |
| Median absolute deviation | 5.000 | 2.810 | | | | | 2.482 | | | |  | |  |  |  |  |  |  |
|  |  |  | | | | |  | | | |  | |  |  |  |  |  |  |
| **Mood test:** | | | |  | |  | | |  |  | |  | |  | |  |  |  |
|  | | | |  | |  | | |  |  | |  | |  | |  |  |  |
| U | | | | 0.000 | |  | | |  |  | |  | |  | |  |  |  |
| Critical value  RunProcRSP Form118.txt CheckBoxTrans,CheckBox,0,False,03,False,Trans,False,,, ListBoxQuanti,ListBox,,True,200000000000_Outputs,True,,False,,, CheckBoxHist,CheckBox,0,True,300000000000_Charts,True,Histograms,False,,, OptionButtonHistBar,OptionButton,-1,True,300000000100_Charts,True,Bars,False,,, OptionButtonHistCont,OptionButton,0,True,300000010100_Charts,True,Continuous line,False,,, CheckBoxCum,CheckBox,0,True,300000000200_Charts,True,Cumulative histograms,False,,, OptionButtonHisBased,OptionButton,-1,True,300000000300_Charts,True,Based on the histogram,False,,, OptionButtonECDF,OptionButton,0,True,300000010300_Charts,True,Empirical cumulative distribution,False,,, CheckBoxRData,CheckBox,0,True,200000000008_Outputs,True,Resamples,False,,, TextBoxConfPer,TextBox,95,True,200000000003_Outputs,True,Confidence interval (%):,False,,, CheckBoxRStat,CheckBox,0,True,200000000007_Outputs,True,Resampled statistics,False,,, CheckBoxNormInt,CheckBox,0,True,200000000004_Outputs,True,Standard bootstrap interval,False,,, CheckBoxPercInt,CheckBox,0,True,200000000005_Outputs,True,Simple percentile interval,False,,, CheckBoxBiasInt,CheckBox,0,True,200000000006_Outputs,True,B.C. percentile interval,False,,, OptionButton_MVRemove,OptionButton,0,True,100000000100_Missing data,True,Remove the observations,False,,, OptionButton_MVEstimate,OptionButton,0,True,100000000300_Missing data,True,Estimate missing data,False,,, OptionButton_MeanMode,OptionButton,-1,True,100000000400_Missing data,True,Mean,False,,, OptionButtonAll,OptionButton,-1,True,100000000200_Missing data,True,For all samples,False,,, OptionButtonRestrict,OptionButton,0,True,100000010200_Missing data,True,For the corresponding sample,False,,, OptionButtonMVRefuse,OptionButton,-1,True,100000000000_Missing data,True,Do not accept missing data,False,,, OptionButton_W,OptionButton,0,True,000000000001_General,True,Workbook,False,,, OptionButton_R,OptionButton,-1,True,000000010001_General,True,Range,False,,, OptionButton_S,OptionButton,0,True,000000020001_General,True,Sheet,False,,, RefEdit_R,RefEdit0,'Sheet8'!$M$67,True,000000000101_General,True,Range:,False,,0,0 CheckBoxVarLabels,CheckBox,0,True,000000000201_General,True,Sample labels,False,,, CheckBox_W,CheckBox,0,True,000000000301_General,True,Weights,False,,, RefEdit_W,RefEdit,,True,000000000401_General,True,Weights:,False,,, TextBox_Resample,TextBox,1000,True,000000000000_General,True,Number of samples:,False,,, RefEdit_X,RefEdit0,'Sheet8'!$J$2:$J$38,True,000000000100_General,True,Quantitative data:,False,,37,1 ComboBoxMethod,ComboBox,0,True,000000010300_General,True,Method:,False,,, TextBox_obs,TextBox,5,True,000000000500_General,True,Sample size:,False,,, ComboBoxChartY,ComboBox,2,True,300000000101_Charts,True,Ordinate of the histograms:,False,,,  1 ListBox 118 ListBoxQuanti  1 22  Sum,0 Mean,-1 Variance (n),-1 Variance (n-1),-1 Standard deviation (n),-1 Standard deviation (n-1),-1 Median,-1 1st Quartile,0 3rd Quartile,0 Variation coefficient,0 Standard error of the mean,0 Mean absolute deviation,0 Median absolute deviation,-1 Geometric mean,0 Geometric standard deviation,0 Harmonic mean,0 1-Percentile,0 99-Percentile,0 2.5-Percentile,0 97.5-Percentile,0 5-Percentile,0 95-Percentile,0  568601  Banks n=37*SEP*Summary statistics*SEP*$M$78 Banks n=37*SEP*Results of the resampling (X1)*SEP*$M$84 | | | | 3.841 | |  | | |  |  | |  | |  | |  |  |  |
| DF | | | | 1.000 | |  | | |  |  | |  | |  | |  |  |  |
| p-value | | | | 1.000 | |  | | |  |  | |  | |  | |  |  |  |
| alpha | | | | 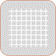0.05 | |  | | |  |  | |  | |  | |  |  |  |
| The p-value has been computed using 10000 Monte Carlo simulations. Time elapsed: 0s. | | | | | | | | | | | | | |  | |  |  |  |
|  | | | |  | |  | | |  |  | |  | |  | |  |  |  |
| Test interpretation: | | | |  | |  | | |  |  | |  | |  | |  |  |  |
| H0: The medians of OBS 1 and OBS 2 are equal. | | | | | |  | | |  |  | |  | |  | |  |  |  |
| Ha: Medians of OBS 1 and OBS 2 are not equal | | | | | |  | | |  |  | |  | |  | |  |  |  |
| As the computed p-value is greater than the significance level alpha=0.05,  one cannot reject the null hypothesis H0. | | | | | | | | | | | | | | | | | |  |
|  |  |  |  |  |  |  |  |  |  |  |  |  |  |  |  |  |  |  |
| **Gas stations** | | | **OBS 1** | | **OBS 2** | | |  |  |  |  |  |  |  |  |  |  |  |
| Automobilgas Via Borzoli | | | 10 | | 10 | | |  |  |  |  |  |  |  |  |  |  |  |
| Automobilgas Via Trensaco | | | 10 | | 10 | | |  |  |  |  |  |  |  |  |  |  |  |
| Barisone Via Ronchi | | | 15 | | 10 | | |  |  |  |  |  |  |  |  |  |  |  |
| ENI Via Archimede | | | 10 | | 10 | | |  |  |  |  |  |  |  |  |  |  |  |
| Esso Cso Europa | | | 10 | | 10 | | |  |  |  |  |  |  |  |  |  |  |  |
| Esso Piazza Dinegro | | | 10 | | 10 | | |  |  |  |  |  |  |  |  |  |  |  |
| Esso Piazza Manin | | | 10 | | 10 | | |  |  |  |  |  |  |  |  |  |  |  |
| Esso Molassana | | | 10 | | 10 | | |  |  |  |  |  |  |  |  |  |  |  |
| Esso Via Lungobisagno | | | 10 | | 10 | | |  |  |  |  |  |  |  |  |  |  |  |
| Esso Via Pegli | | | 10 | | 10 | | |  |  |  |  |  |  |  |  |  |  |  |
| Europam Cso Magenta | | | 10 | | 10 | | |  |  |  |  |  |  |  |  |  |  |  |
| Europam Via Borzoli | | | 10 | | 10 | | |  |  |  |  |  |  |  |  |  |  |  |
| Europam Via Bianchi | | | 10 | | 10 | | |  |  |  |  |  |  |  |  |  |  |  |
| IP Via Cantore | | | 10 | | 10 | | |  |  |  |  |  |  |  |  |  |  |  |
| IP Via Diaz | | | 10 | | 10 | | |  |  |  |  |  |  |  |  |  |  |  |
| IP Via Ferri | | | 10 | | 10 | | |  |  |  |  |  |  |  |  |  |  |  |
| Q8 Cso Europa | | | 10 | | 10 | | |  |  |  |  |  |  |  |  |  |  |  |
| Q8 S.Ilario | | | 15 | | 15 | | |  |  |  |  |  |  |  |  |  |  |  |
| Q8 Lanterna Est | | | 10 | | 10 | | |  |  |  |  |  |  |  |  |  |  |  |
| Q8 Via Adamoli | | | 10 | | 10 | | |  |  |  |  |  |  |  |  |  |  |  |
| Q8 Via dei Mille | | | 10 | | 10 | | |  |  |  |  |  |  |  |  |  |  |  |
| Q8 Via Multedo | | | 10 | | 10 | | |  |  |  |  |  |  |  |  |  |  |  |
| Q8 Via Vasco de Gama | | | 10 | | 10 | | |  |  |  |  |  |  |  |  |  |  |  |
| Stazione di Servizio Via Lemerle | | | 10 | | 10 | | |  |  |  |  |  |  |  |  |  |  |  |
| Tamoil Via Canepari | | | 10 | | 10 | | |  |  |  |  |  |  |  |  |  |  |  |
| Tamoil Corso Europa | | | 10 | | 10 | | |  |  |  |  |  |  |  |  |  |  |  |
|  | | |  | |  | | |  |  |  |  |  |  |  |  |  |  |  |

| **Descriptives** | | **OBS 1** | | **OBS 2** | |  | |  | | |  | |  | |  | |  | | |  |  |
| --- | --- | --- | --- | --- | --- | --- | --- | --- | --- | --- | --- | --- | --- | --- | --- | --- | --- | --- | --- | --- | --- |
| Nbr. of observations | | 26 | | 26 | |  | |  | | |  | |  | |  | |  | | |  |  |
| Nbr. of missing values | | 0 | | 0 | |  | |  | | |  | |  | |  | |  | | |  |  |
| Obs. without missing data | | 26 | | 26 | |  | |  | | |  | |  | |  | |  | | |  |  |
| Minimum | | 10.000 | | 10.000 | |  | |  | | |  | |  | |  | |  | | |  |  |
| Maximum | | 15.000 | | 15.000 | |  | |  | | |  | |  | |  | |  | | |  |  |
| Freq. of minimum | | 24 | | 25 | |  | |  | | |  | |  | |  | |  | | |  |  |
| Freq. of maximum | | 2 | | 1 | |  | |  | | |  | |  | |  | |  | | |  |  |
| Median | | 10.000 | | 10.000 | |  | |  | | |  | |  | |  | |  | | |  |  |
| Mean | | 10.385 | | 10.192 | |  | |  | | |  | |  | |  | |  | | |  |  |
| Variance (n-1) | | 1.846 | | 0.962 | |  | |  | | |  | |  | |  | |  | | |  |  |
| Standard deviation (n-1) | | 1.359 | | 0.981 | |  | |  | | |  | |  | |  | |  | | |  |  |
| Median absolute deviation | | 0.000 | | 0.000 | |  | |  | | |  | |  | |  | |  | | |  |  |
|  | |  | |  | |  | |  | | |  | |  | |  | |  | | |  |  |
| **Shapiro-Wilk test (OBS 1):** | |  | |  | |  | |  | | |  | |  | |  | |  | | |  |  |
|  | |  | |  | |  | |  | | |  | |  | |  | |  | | |  |  |
| W | | 0.301 | |  | |  | |  | | |  | |  | |  | |  | | |  |  |
| p-value (Two-tailed) | | **<0.0001** | |  | |  | |  | | |  | |  | |  | |  | | |  |  |
| alpha  Gas stations n=26*SEP*Summary statistics*SEP*$M$39 Gas stations n=26*SEP*Results of the resampling (X1)*SEP*$M$45 | | 0.05 | |  | |  | |  | | |  | |  | |  | |  | | |  |  |
|  | |  | |  | |  | |  | | |  | |  | |  | |  | | |  |  |
| Test interpretation: | |  | |  | |  | |  | | |  | |  | |  | |  | | |  |  |
| H0: The variable from which the sample was extracted follows a Normal distribution. | | | | | | | | | | | | |  | |  | |  | | |  |  |
| Ha: The variable from which the sample was extracted does not follow a Normal distribution. | | | | | | | | | | | | | | |  | |  | | |  |  |
| As the computed p-value is lower than the significance level alpha=0.05,  one should reject the null hypothesis H0, and accept the alternative hypothesis Ha. | | | | | | | | | | | | | | | | | | | |  |  |
|  |  |  |  |  |  |  |  |  |  |  |  |  |  |  |  |  |  |  |  |  |  |
|  | |  | |  | |  | |  | | |  | |  | |  | |  | | |  |  |
| **Shapiro-Wilk test (OBS 2):** | | |  | |  | |  | | |  | |  | |  | |  | |  |  |  |  |
|  |  | |  | |  | |  | | |  | |  | |  | |  | |  |  |  |  |
| W | 0.198 | |  | |  | |  | | |  | |  | |  | |  | |  |  |  |  |
| p-value (Two-tailed) | **<0.0001** | |  | |  | |  | | |  | |  | |  | |  | |  |  |  |  |
| alpha | 0.05 | |  | |  | |  | | |  | |  | |  | |  | |  |  |  |  |
|  |  | |  | |  | |  | | |  | |  | |  | |  | |  |  |  |  |
| Test interpretation: |  | |  | |  | |  | | |  | |  | |  | |  | |  |  |  |  |
| H0: The variable from which the sample was extracted follows a Normal distribution. | | | | | | | | | | | | | |  | |  | |  |  |  |  |
| Ha: The variable from which the sample was extracted does not follow a Normal distribution. | | | | | | | | | | | | | | | |  | |  |  |  |  |
| As the computed p-value is lower than the significance level alpha=0.05,  one should reject the null hypothesis H0, and accept the alternative hypothesis Ha. | | | | | | | | | | | | | | | | | |  |  |  |  |
|  |  |  |  |  |  |  |  |  |  |  |  |  |  |  |  |  |  |  |  |  |  |
|  |  | |  | |  | |  | | |  | |  | |  | |  | |  |  |  |  |
| **Results of the resampling (OBS 1):** | | | |  | | | | |  | | | | | |  | |  | | |  | |
| RunProcRSP Form118.txt CheckBoxTrans,CheckBox,0,False,03,False,Trans,False,,, ListBoxQuanti,ListBox,,True,200000000000_Outputs,True,,False,,, CheckBoxHist,CheckBox,0,True,300000000000_Charts,True,Histograms,False,,, OptionButtonHistBar,OptionButton,-1,True,300000000100_Charts,True,Bars,False,,, OptionButtonHistCont,OptionButton,0,True,300000010100_Charts,True,Continuous line,False,,, CheckBoxCum,CheckBox,0,True,300000000200_Charts,True,Cumulative histograms,False,,, OptionButtonHisBased,OptionButton,-1,True,300000000300_Charts,True,Based on the histogram,False,,, OptionButtonECDF,OptionButton,0,True,300000010300_Charts,True,Empirical cumulative distribution,False,,, CheckBoxRData,CheckBox,0,True,200000000008_Outputs,True,Resamples,False,,, TextBoxConfPer,TextBox,95,True,200000000003_Outputs,True,Confidence interval (%):,False,,, CheckBoxRStat,CheckBox,0,True,200000000007_Outputs,True,Resampled statistics,False,,, CheckBoxNormInt,CheckBox,0,True,200000000004_Outputs,True,Standard bootstrap interval,False,,, CheckBoxPercInt,CheckBox,0,True,200000000005_Outputs,True,Simple percentile interval,False,,, CheckBoxBiasInt,CheckBox,0,True,200000000006_Outputs,True,B.C. percentile interval,False,,, OptionButton_MVRemove,OptionButton,0,True,100000000100_Missing data,True,Remove the observations,False,,, OptionButton_MVEstimate,OptionButton,0,True,100000000300_Missing data,True,Estimate missing data,False,,, OptionButton_MeanMode,OptionButton,-1,True,100000000400_Missing data,True,Mean,False,,, OptionButtonAll,OptionButton,-1,True,100000000200_Missing data,True,For all samples,False,,, OptionButtonRestrict,OptionButton,0,True,100000010200_Missing data,True,For the corresponding sample,False,,, OptionButtonMVRefuse,OptionButton,-1,True,100000000000_Missing data,True,Do not accept missing data,False,,, OptionButton_W,OptionButton,0,True,000000000001_General,True,Workbook,False,,, OptionButton_R,OptionButton,-1,True,000000010001_General,True,Range,False,,, OptionButton_S,OptionButton,0,True,000000020001_General,True,Sheet,False,,, RefEdit_R,RefEdit0,'Sheet9'!$M$28,True,000000000101_General,True,Range:,False,,1,1 CheckBoxVarLabels,CheckBox,0,True,000000000201_General,True,Sample labels,False,,, CheckBox_W,CheckBox,0,True,000000000301_General,True,Weights,False,,, RefEdit_W,RefEdit,,True,000000000401_General,True,Weights:,False,,, TextBox_Resample,TextBox,1000,True,000000000000_General,True,Number of samples:,False,,, RefEdit_X,RefEdit0,'Sheet9'!$I$2:$I$27,True,000000000100_General,True,Quantitative data:,False,,26,1 ComboBoxMethod,ComboBox,0,True,000000010300_General,True,Method:,False,,, TextBox_obs,TextBox,5,True,000000000500_General,True,Sample size:,False,,, ComboBoxChartY,ComboBox,2,True,300000000101_Charts,True,Ordinate of the histograms:,False,,,  1 ListBox 118 ListBoxQuanti  1 22  Sum,0 Mean,-1 Variance (n),-1 Variance (n-1),-1 Standard deviation (n),-1 Standard deviation (n-1),-1 Median,-1 1st Quartile,0 3rd Quartile,0 Variation coefficient,0 Standard error of the mean,0 Mean absolute deviation,0 Median absolute deviation,-1 Geometric mean,0 Geometric standard deviation,0 Harmonic mean,0 1-Percentile,0 99-Percentile,0 2.5-Percentile,0 97.5-Percentile,0 5-Percentile,0 95-Percentile,0  331174 | |  | |  | | | | |  | | | | | |  | |  | | |  | |
| Parameters | | Estimator | | Estimator (Bootstrap) | | | | | Standard deviation (Bootstrap) | | | | | |  | |  | | |  | |
| Mean | | 10.385 | | 10.384 | | | | | 0.258 | | | | | |  | |  | | |  | |
| Variance (n) | | 1.775 | | 1.707 | | | | | 1.060 | | | | | |  | |  | | |  | |
| Variance (n-1) | | 1.846 | | 1.775 | | | | | 1.103 | | | | | |  | |  | | |  | |
| Standard deviation (n) | | 1.332 | | 1.190 | | | | | 0.540 | | | | | |  | |  | | |  | |
| Standard deviation (n-1) | | 1.359 | | 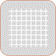1.214 | | | | | 0.550 | | | | | |  | |  | | |  | |
| Median | | 10.000 | | 10.000 | | | | | 0.000 | | | | | |  | |  | | |  | |
| Median absolute deviation | | 0.000 | | 0.000 | | | | | 0.000 | | | | | |  | |  | | |  | |
|  | |  | |  | | | | |  | | | | | |  | |  | | |  | |

| **Results of the resampling (OBS 2):** | | | | | | | | |  | | | | |  | |  | |  | |  |  |  |
| --- | --- | --- | --- | --- | --- | --- | --- | --- | --- | --- | --- | --- | --- | --- | --- | --- | --- | --- | --- | --- | --- | --- |
|  |  |  | | | | | | |  | | | | |  | |  | |  | |  |  |  |
| Parameters | Estimator | Estimator (Bootstrap) | | | | | | | Standard deviation (Bootstrap) | | | | |  | |  | |  | |  |  |  |
| Mean | 10.192 | 10.194 | | | | | | | 0.193 | | | | |  | |  | |  | |  |  |  |
| Variance (n) | 0.925 | 0.894 | | | | | | | 0.854 | | | | |  | |  | |  | |  |  |  |
| Variance (n-1) | 0.962 | 0.929 | | | | | | | 0.888 | | | | |  | |  | |  | |  |  |  |
| Standard deviation (n) | 0.962 | 0.737 | | | | | | | 0.592 | | | | |  | |  | |  | |  |  |  |
| Standard deviation (n-1) | 0.981 | 0.752 | | | | | | | 0.604 | | | | |  | |  | |  | |  |  |  |
| Median | 10.000 | 10.000 | | | | | | | 0.000 | | | | |  | |  | |  | |  |  |  |
| Median absolute deviation | 0.000 | 0.000 | | | | | | | 0.000 | | | | |  | |  | |  | |  |  |  |
|  |  |  | | | | | | |  | | | | |  | |  | |  | |  |  |  |
| **Mood test:** | | |  | | |  | | | |  | | |  | |  | |  | |  | |  |  |
| RunProcRSP Form118.txt CheckBoxTrans,CheckBox,0,False,03,False,Trans,False,,, ListBoxQuanti,ListBox,,True,200000000000_Outputs,True,,False,,, CheckBoxHist,CheckBox,0,True,300000000000_Charts,True,Histograms,False,,, OptionButtonHistBar,OptionButton,-1,True,300000000100_Charts,True,Bars,False,,, OptionButtonHistCont,OptionButton,0,True,300000010100_Charts,True,Continuous line,False,,, CheckBoxCum,CheckBox,0,True,300000000200_Charts,True,Cumulative histograms,False,,, OptionButtonHisBased,OptionButton,-1,True,300000000300_Charts,True,Based on the histogram,False,,, OptionButtonECDF,OptionButton,0,True,300000010300_Charts,True,Empirical cumulative distribution,False,,, CheckBoxRData,CheckBox,0,True,200000000008_Outputs,True,Resamples,False,,, TextBoxConfPer,TextBox,95,True,200000000003_Outputs,True,Confidence interval (%):,False,,, CheckBoxRStat,CheckBox,0,True,200000000007_Outputs,True,Resampled statistics,False,,, CheckBoxNormInt,CheckBox,0,True,200000000004_Outputs,True,Standard bootstrap interval,False,,, CheckBoxPercInt,CheckBox,0,True,200000000005_Outputs,True,Simple percentile interval,False,,, CheckBoxBiasInt,CheckBox,0,True,200000000006_Outputs,True,B.C. percentile interval,False,,, OptionButton_MVRemove,OptionButton,0,True,100000000100_Missing data,True,Remove the observations,False,,, OptionButton_MVEstimate,OptionButton,0,True,100000000300_Missing data,True,Estimate missing data,False,,, OptionButton_MeanMode,OptionButton,-1,True,100000000400_Missing data,True,Mean,False,,, OptionButtonAll,OptionButton,-1,True,100000000200_Missing data,True,For all samples,False,,, OptionButtonRestrict,OptionButton,0,True,100000010200_Missing data,True,For the corresponding sample,False,,, OptionButtonMVRefuse,OptionButton,-1,True,100000000000_Missing data,True,Do not accept missing data,False,,, OptionButton_W,OptionButton,0,True,000000000001_General,True,Workbook,False,,, OptionButton_R,OptionButton,-1,True,000000010001_General,True,Range,False,,, OptionButton_S,OptionButton,0,True,000000020001_General,True,Sheet,False,,, RefEdit_R,RefEdit0,'Sheet9'!$M$56,True,000000000101_General,True,Range:,False,,0,0 CheckBoxVarLabels,CheckBox,0,True,000000000201_General,True,Sample labels,False,,, CheckBox_W,CheckBox,0,True,000000000301_General,True,Weights,False,,, RefEdit_W,RefEdit,,True,000000000401_General,True,Weights:,False,,, TextBox_Resample,TextBox,1000,True,000000000000_General,True,Number of samples:,False,,, RefEdit_X,RefEdit0,'Sheet9'!$J$2:$J$27,True,000000000100_General,True,Quantitative data:,False,,26,1 ComboBoxMethod,ComboBox,0,True,000000010300_General,True,Method:,False,,, TextBox_obs,TextBox,5,True,000000000500_General,True,Sample size:,False,,, ComboBoxChartY,ComboBox,2,True,300000000101_Charts,True,Ordinate of the histograms:,False,,,  1 ListBox 118 ListBoxQuanti  1 22  Sum,0 Mean,-1 Variance (n),-1 Variance (n-1),-1 Standard deviation (n),-1 Standard deviation (n-1),-1 Median,-1 1st Quartile,0 3rd Quartile,0 Variation coefficient,0 Standard error of the mean,0 Mean absolute deviation,0 Median absolute deviation,-1 Geometric mean,0 Geometric standard deviation,0 Harmonic mean,0 1-Percentile,0 99-Percentile,0 2.5-Percentile,0 97.5-Percentile,0 5-Percentile,0 95-Percentile,0  768287  Gas stations n=26*SEP*Summary statistics*SEP*$M$67 Gas stations n=26*SEP*Results of the resampling (X1)*SEP*$M$73 | | |  | | |  | | | |  | | |  | |  | |  | |  | |  |  |
| U | | | 0.000 | | |  | | | |  | | |  | |  | |  | |  | |  |  |
| Critical value | | | 3.841 | | |  | | | |  | | |  | |  | |  | |  | |  |  |
| DF | | | 1.000 | | |  | | | |  | | |  | |  | |  | |  | |  |  |
| p-value | | | 1.000 | | |  | | | |  | | |  | |  | |  | |  | |  |  |
| alpha | | | 0.05 | | |  | | | |  | | |  | |  | |  | |  | |  |  |
| The p-value has been computed using 10000 Monte Carlo simulations. Time elapsed: 0s. | | | | | | | | | | | | | | | | | | |  | |  |  |
|  | | |  | | |  | | | |  | | |  | |  | |  | |  | |  |  |
| Test interpretation: | | |  | | |  | | | |  | | |  | |  | |  | |  | |  |  |
| H0: The medians of OBS 1 and OBS 2 are equal. | | | | | | | | | |  | | |  | |  | |  | |  | |  |  |
| Ha: Medians of OBS 1 and OBS 2 are not equal | | | | | | | | | |  | | |  | |  | |  | |  | |  |  |
| As the computed p-value is greater than the significance level alpha=0.05,  RunProcRSP Form118.txt CheckBoxTrans,CheckBox,0,False,03,False,Trans,False,,, ListBoxQuanti,ListBox,,True,200000000000_Outputs,True,,False,,, CheckBoxHist,CheckBox,0,True,300000000000_Charts,True,Histograms,False,,, OptionButtonHistBar,OptionButton,-1,True,300000000100_Charts,True,Bars,False,,, OptionButtonHistCont,OptionButton,0,True,300000010100_Charts,True,Continuous line,False,,, CheckBoxCum,CheckBox,0,True,300000000200_Charts,True,Cumulative histograms,False,,, OptionButtonHisBased,OptionButton,-1,True,300000000300_Charts,True,Based on the histogram,False,,, OptionButtonECDF,OptionButton,0,True,300000010300_Charts,True,Empirical cumulative distribution,False,,, CheckBoxRData,CheckBox,0,True,200000000008_Outputs,True,Resamples,False,,, TextBoxConfPer,TextBox,95,True,200000000003_Outputs,True,Confidence interval (%):,False,,, CheckBoxRStat,CheckBox,0,True,200000000007_Outputs,True,Resampled statistics,False,,, CheckBoxNormInt,CheckBox,0,True,200000000004_Outputs,True,Standard bootstrap interval,False,,, CheckBoxPercInt,CheckBox,0,True,200000000005_Outputs,True,Simple percentile interval,False,,, CheckBoxBiasInt,CheckBox,0,True,200000000006_Outputs,True,B.C. percentile interval,False,,, OptionButton_MVRemove,OptionButton,0,True,100000000100_Missing data,True,Remove the observations,False,,, OptionButton_MVEstimate,OptionButton,0,True,100000000300_Missing data,True,Estimate missing data,False,,, OptionButton_MeanMode,OptionButton,-1,True,100000000400_Missing data,True,Mean,False,,, OptionButtonAll,OptionButton,-1,True,100000000200_Missing data,True,For all samples,False,,, OptionButtonRestrict,OptionButton,0,True,100000010200_Missing data,True,For the corresponding sample,False,,, OptionButtonMVRefuse,OptionButton,-1,True,100000000000_Missing data,True,Do not accept missing data,False,,, OptionButton_W,OptionButton,0,True,000000000001_General,True,Workbook,False,,, OptionButton_R,OptionButton,-1,True,000000010001_General,True,Range,False,,, OptionButton_S,OptionButton,0,True,000000020001_General,True,Sheet,False,,, RefEdit_R,RefEdit0,'Sheet9'!$M$84,True,000000000101_General,True,Range:,False,,0,0 CheckBoxVarLabels,CheckBox,0,True,000000000201_General,True,Sample labels,False,,, CheckBox_W,CheckBox,0,True,000000000301_General,True,Weights,False,,, RefEdit_W,RefEdit,,True,000000000401_General,True,Weights:,False,,, TextBox_Resample,TextBox,1000,True,000000000000_General,True,Number of samples:,False,,, RefEdit_X,RefEdit0,'Sheet9'!$K$2:$K$26,True,000000000100_General,True,Quantitative data:,False,,25,1 ComboBoxMethod,ComboBox,0,True,000000010300_General,True,Method:,False,,, TextBox_obs,TextBox,5,True,000000000500_General,True,Sample size:,False,,, ComboBoxChartY,ComboBox,2,True,300000000101_Charts,True,Ordinate of the histograms:,False,,,  1 ListBox 118 ListBoxQuanti  1 22  Sum,0 Mean,-1 Variance (n),-1 Variance (n-1),-1 Standard deviation (n),-1 Standard deviation (n-1),-1 Median,-1 1st Quartile,0 3rd Quartile,0 Variation coefficient,0 Standard error of the mean,0 Mean absolute deviation,0 Median absolute deviation,-1 Geometric mean,0 Geometric standard deviation,0 Harmonic mean,0 1-Percentile,0 99-Percentile,0 2.5-Percentile,0 97.5-Percentile,0 5-Percentile,0 95-Percentile,0  804488  Gas stations n=26*SEP*Summary statistics*SEP*$M$95 Gas stations n=26*SEP*Results of the resampling (X1)*SEP*$M$101  one cannot reject the null hypothesis H0. | | | | | | | | | | | | | | | | | | | | | |  |
|  |  |  |  |  |  |  |  |  |  |  |  |  |  |  |  |  |  |  |  |  |  |  |
| **Pharmacies** | | | | | **OBS 1** | | | **OBS 2** | | | |  |  |  |  |  |  |  |  |  |  |  |
| Farmacia Alvigini | | | | | 15 | | | 15 | | | |  |  |  |  |  |  |  |  |  |  |  |
| Farmacia Amoretti | | | | | 15 | | | 15 | | | |  |  |  |  |  |  |  |  |  |  |  |
| Farmacia Arte Farmaceutica | | | | | 15 | | | 15 | | | |  |  |  |  |  |  |  |  |  |  |  |
| Farmacia Assarotti | | | | | 10 | | | 10 | | | |  |  |  |  |  |  |  |  |  |  |  |
| Farmacia Barabino | | | | | 15 | | | 15 | | | |  |  |  |  |  |  |  |  |  |  |  |
| Farmacia Bassano | | | | | 20 | | | 20 | | | |  |  |  |  |  |  |  |  |  |  |  |
| Farmacia Baudoin | | | | | 15 | | | 15 | | | |  |  |  |  |  |  |  |  |  |  |  |
| Farmacia Bonanni | | | | | 15 | | | 15 | | | |  |  |  |  |  |  |  |  |  |  |  |
| Farmacia Burlando | | | | | 20 | | | 10 | | | |  |  |  |  |  |  |  |  |  |  |  |
| Farmacia Campart | | | | | 10 | | | 15 | | | |  |  |  |  |  |  |  |  |  |  |  |
| Farmacia Canevari | | | | | 15 | | | 15 | | | |  |  |  |  |  |  |  |  |  |  |  |
| Farmacia Cantore | | | | | 15 | | | 15 | | | |  |  |  |  |  |  |  |  |  |  |  |
| Farmacia Cappuccini | | | | | 15 | | | 15 | | | |  |  |  |  |  |  |  |  |  |  |  |
| Farmacia Carlevaro | | | | | 15 | | | 15 | | | |  |  |  |  |  |  |  |  |  |  |  |
| Farmacia Castelletto | | | | | 15 | | | 15 | | | |  |  |  |  |  |  |  |  |  |  |  |
| Farmacia Centrale | | | | | 15 | | | 15 | | | |  |  |  |  |  |  |  |  |  |  |  |
| Farmacia Centrale | | | | | 15 | | | 15 | | | |  |  |  |  |  |  |  |  |  |  |  |
| Farmacia Certosa | | | | | 15 | | | 15 | | | |  |  |  |  |  |  |  |  |  |  |  |
| Farmacia Comunale | | | | | 15 | | | 15 | | | |  |  |  |  |  |  |  |  |  |  |  |
| Farmacia Comunale 2 | | | | | 15 | | | 15 | | | |  |  |  |  |  |  |  |  |  |  |  |
| Farmacia Comunale Burlando | | | | | 15 | | | 15 | | | |  |  |  |  |  |  |  |  |  |  |  |
| Farmacia Comunale Molassana | | | | | 15 | | | 15 | | | |  |  |  |  |  |  |  |  |  |  |  |
| Farmacia Comunale Sestri Ponente | | | | | 15 | | | 15 | | | |  |  |  |  |  |  |  |  |  |  |  |
| Farmacia Dagnino | | | | | 15 | | | 15 | | | |  |  |  |  |  |  |  |  |  |  |  |
| Farmacia Dapelo | | | | | 15 | | | 15 | | | |  |  |  |  |  |  |  |  |  |  |  |
| Farmacia de Ferrari | | | | | 15 | | | 15 | | | |  |  |  |  |  |  |  |  |  |  |  |
| Farmacia Del Chiappazzo | | | | | 15 | | | 15 | | | |  |  |  |  |  |  |  |  |  |  |  |
| Farmacia della Nunziata | | | | | 15 | | | 15 | | | |  |  |  |  |  |  |  |  |  |  |  |
| Farmacia dell'Aquila | | | | | 15 | | | 20 | | | |  |  |  |  |  |  |  |  |  |  |  |
| Farmacia Dell'Oro | | | | | 10 | | | 10 | | | |  |  |  |  |  |  |  |  |  |  |  |
| Farmacia Europa | | | | | 10 | | | 10 | | | |  |  |  |  |  |  |  |  |  |  |  |
| Farmacia Gallo | | | | | 15 | | | 15 | | | |  |  |  |  |  |  |  |  |  |  |  |
| Farmacia Genovese | | | | | 15 | | | 15 | | | |  |  |  |  |  |  |  |  |  |  |  |
| Farmacia Ghersi | | | | | 10 | | | 15 | | | |  |  |  |  |  |  |  |  |  |  |  |
| Farmacia Giusto | | | | | 15 | | | 15 | | | |  |  |  |  |  |  |  |  |  |  |  |
| Farmacia Iachetti | | | | | 10 | | | 15 | | | |  |  |  |  |  |  |  |  |  |  |  |
| Farmacia Imperiale | | | | | 15 | | | 15 | | | |  |  |  |  |  |  |  |  |  |  |  |
| Farmacia Lagaccio | | | | | 15 | | | 15 | | | |  |  |  |  |  |  |  |  |  |  |  |
| Farmacia Ligure | | | | | 15 | | | 15 | | | |  |  |  |  |  |  |  |  |  |  |  |
| Farmacia Lloyds | | | | | 15 | | | 15 | | | |  |  |  |  |  |  |  |  |  |  |  |
| Farmacia Lloyds albaro | | | | | 15 | | | 15 | | | |  |  |  |  |  |  |  |  |  |  |  |
| Farmacia Lloyds Castelletto | | | | | 15 | | | 15 | | | |  |  |  |  |  |  |  |  |  |  |  |
| Farmacia Majonchi | | | | | 10 | | | 10 | | | |  |  |  |  |  |  |  |  |  |  |  |
| Farmacia Martinelli | | | | | 15 | | | 15 | | | |  |  |  |  |  |  |  |  |  |  |  |
| Farmacia Massa | | | | | 15 | | | 15 | | | |  |  |  |  |  |  |  |  |  |  |  |
| Farmacia Moderna | | | | | 15 | | | 15 | | | |  |  |  |  |  |  |  |  |  |  |  |
| Farmacia Modigliani | | | | | 15 | | | 15 | | | |  |  |  |  |  |  |  |  |  |  |  |
| Farmacia Monticelli | | | | | 15 | | | 15 | | | |  |  |  |  |  |  |  |  |  |  |  |
| Farmacia Multedo | | | | | 10 | | | 10 | | | |  |  |  |  |  |  |  |  |  |  |  |
| Farmacia Nazionale | | | | | 15 | | | 15 | | | |  |  |  |  |  |  |  |  |  |  |  |
| Farmacia Nizza | | | | | 15 | | | 15 | | | |  |  |  |  |  |  |  |  |  |  |  |
| Farmacia Nostra Signora del monte | | | | | 15 | | | 15 | | | |  |  |  |  |  |  |  |  |  |  |  |
| Farmacia Nuovo Lido di Albaro | | | | | 10 | | | 15 | | | |  |  |  |  |  |  |  |  |  |  |  |
| Farmacia Oregina | | | | | 15 | | | 15 | | | |  |  |  |  |  |  |  |  |  |  |  |
| Farmacia Orientale | | | | | 15 | | | 15 | | | |  |  |  |  |  |  |  |  |  |  |  |
| Farmacia Palmaro | | | | | 15 | | | 15 | | | |  |  |  |  |  |  |  |  |  |  |  |
| Farmacia Pedrini | | | | | 15 | | | 15 | | | |  |  |  |  |  |  |  |  |  |  |  |
| Farmacia Pescetto | | | | | 15 | | | 15 | | | |  |  |  |  |  |  |  |  |  |  |  |
| Farmacia Piaggio | | | | | 15 | | | 15 | | | |  |  |  |  |  |  |  |  |  |  |  |
| Farmacia Ponte Monumentale | | | | | 15 | | | 15 | | | |  |  |  |  |  |  |  |  |  |  |  |
| Farmacia Ponte Pila | | | | | 15 | | | 15 | | | |  |  |  |  |  |  |  |  |  |  |  |
| Farmacia Popolare | | | | | 15 | | | 15 | | | |  |  |  |  |  |  |  |  |  |  |  |
| Farmacia Quarto dei Mille | | | | | 15 | | | 15 | | | |  |  |  |  |  |  |  |  |  |  |  |
| Farmacia Ribaldone | | | | | 10 | | | 10 | | | |  |  |  |  |  |  |  |  |  |  |  |
| Farmacia Saltarelli | | | | | 15 | | | 15 | | | |  |  |  |  |  |  |  |  |  |  |  |
| Farmacia San Bernardo | | | | | 15 | | | 15 | | | |  |  |  |  |  |  |  |  |  |  |  |
| Farmacia san Giacomo | | | | | 15 | | | 15 | | | |  |  |  |  |  |  |  |  |  |  |  |
| Farmacia San Giorgio | | | | | 10 | | | 15 | | | |  |  |  |  |  |  |  |  |  |  |  |
| Farmacia San Pietro | | | | | 15 | | | 15 | | | |  |  |  |  |  |  |  |  |  |  |  |
| Farmacia San Raffaele | | | | | 10 | | | 15 | | | |  |  |  |  |  |  |  |  |  |  |  |
| Farmacia San Rocco | | | | | 15 | | | 15 | | | |  |  |  |  |  |  |  |  |  |  |  |
| Farmacia San Sebastiano | | | | | 15 | | | 15 | | | |  |  |  |  |  |  |  |  |  |  |  |
| Farmacia Sangiorgi | | | | | 15 | | | 15 | | | |  |  |  |  |  |  |  |  |  |  |  |
| Farmacia Sanitas | | | | | 15 | | | 15 | | | |  |  |  |  |  |  |  |  |  |  |  |
| Farmacia Santa Rosa | | | | | 15 | | | 15 | | | |  |  |  |  |  |  |  |  |  |  |  |
| Farmacia Sarzano | | | | | 15 | | | 15 | | | |  |  |  |  |  |  |  |  |  |  |  |
| Farmacia Scanavino | | | | | 10 | | | 15 | | | |  |  |  |  |  |  |  |  |  |  |  |
| Farmacia Sturla | | | | | 10 | | | 10 | | | |  |  |  |  |  |  |  |  |  |  |  |
| Farmacia Terrile | | | | | 15 | | | 15 | | | |  |  |  |  |  |  |  |  |  |  |  |
| Farmacia Vesuvio | | | | | 15 | | | 15 | | | |  |  |  |  |  |  |  |  |  |  |  |
|  | | | | |  | | |  | | | |  |  |  |  |  |  |  |  |  |  |  |
| **Descriptives** | | | | **OBS 1** | | | **OBS 2** | | | |  | |  | |  | |  | |  | |  |  |
| Nbr. of observations | | | | 80 | | | 80 | | | |  | |  | |  | |  | |  | |  |  |
| Nbr. of missing values | | | | 0 | | | 0 | | | |  | |  | |  | |  | |  | |  |  |
| Obs. without missing data | | | | 80 | | | 80 | | | |  | |  | |  | |  | |  | |  |  |
| Minimum | | | | 10.000 | | | 10.000 | | | |  | |  | |  | |  | |  | |  |  |
| Maximum | | | | 20.000 | | | 20.000 | | | |  | |  | |  | |  | |  | |  |  |
| Freq. of minimum | | | | 14 | | | 8 | | | |  | |  | |  | |  | |  | |  |  |
| Freq. of maximum | | | | 2 | | | 2 | | | |  | |  | |  | |  | |  | |  |  |
| Median | | | | 15.000 | | | 15.000 | | | |  | |  | |  | |  | |  | |  |  |
| Mean | | | | 14.250 | | | 14.625 | | | |  | |  | |  | |  | |  | |  |  |
| Variance (n-1) | | | | 4.494 | | | 3.022 | | | |  | |  | |  | |  | |  | |  |  |
| Standard deviation (n-1) | | | | 2.120 | | | 1.738 | | | |  | |  | |  | |  | |  | |  |  |
| Median absolute deviation | | | | 0.000 | | | 0.000 | | | |  | |  | |  | |  | |  | |  |  |
|  | | | |  | | |  | | | |  | |  | |  | |  | |  | |  |  |
| **Shapiro-Wilk test (OBS 1):** | | | |  | | |  | | | |  | |  | |  | |  | |  | |  |  |
|  | | | |  | | |  | | | |  | |  | |  | |  | |  | |  |  |
| W | | | | 0.566 | | |  | | | |  | |  | |  | |  | |  | |  |  |
| p-value (Two-tailed) | | | | **<0.0001** | | |  | | | |  | |  | |  | |  | |  | |  |  |
| alpha | | | | 0.05 | | |  | | | |  | |  | |  | |  | |  | |  |  |
| Pharmacies n=80*SEP*Summary statistics*SEP*$M$93 Pharmacies n=80*SEP*Results of the resampling (X1)*SEP*$M$99 | | | |  | | |  | | | |  | |  | |  | |  | |  | |  |  |
| Test interpretation: | | | |  | | |  | | | |  | |  | |  | |  | |  | |  |  |
| H0: The variable from which the sample was extracted follows a Normal distribution. | | | | | | | | | | | | | | | | |  | |  | |  |  |
| Ha: The variable from which the sample was extracted does not follow a Normal distribution. | | | | | | | | | | | | | | | | | | |  | |  |  |
| As the computed p-value is lower than the significance level alpha=0.05,  one should reject the null hypothesis H0, and accept the alternative hypothesis Ha. | | | | | | | | | | | | | | | | | | | | | |  |
|  |  |  |  |  |  |  |  |  |  |  |  |  |  |  |  |  |  |  |  |  |  |  |
|  | | | |  | | |  | | | |  | |  | |  | |  | |  | |  |  |

| **Shapiro-Wilk test (OBS 2):** | | | | | |  | | |  | | |  | | |  | | |  | | | |  | | |  | |  | | |
| --- | --- | --- | --- | --- | --- | --- | --- | --- | --- | --- | --- | --- | --- | --- | --- | --- | --- | --- | --- | --- | --- | --- | --- | --- | --- | --- | --- | --- | --- |
|  | |  | | | |  | | |  | | |  | | |  | | |  | | | |  | | |  | |  | | |
| W | | 0.462 | | | |  | | |  | | |  | | |  | | |  | | | |  | | |  | |  | | |
| p-value (Two-tailed) | | **<0.0001** | | | |  | | |  | | |  | | |  | | |  | | | |  | | |  | |  | | |
| alpha | | 0.05 | | | |  | | |  | | |  | | |  | | |  | | | |  | | |  | |  | | |
|  | |  | | | |  | | |  | | |  | | |  | | |  | | | |  | | |  | |  | | |
| Test interpretation: | |  | | | |  | | |  | | |  | | |  | | |  | | | |  | | |  | |  | | |
| H0: The variable from which the sample was extracted follows a Normal distribution. | | | | | | | | | | | | | | | | | | | | | |  | | |  | |  | | |
| Ha: The variable from which the sample was extracted does not follow a Normal distribution. | | | | | | | | | | | | | | | | | | | | | | | | |  | |  | | |
| As the computed p-value is lower than the significance level alpha=0.05,  one should reject the null hypothesis H0, and accept the alternative hypothesis Ha. | | | | | | | | | | | | | | | | | | | | | | | | | | |  | | |
|  |  |  |  |  |  |  |  |  |  |  |  |  |  |  |  |  |  |  |  |  |  |  |  |  |  |  |  | | |
|  | |  | | | |  | | |  | | |  | | |  | | |  | | | |  | | |  | |  | | |
| **Results of the resampling (OBS 1):** | | | | | |  | | | | |  | | | | | | |  | | |  | | |  |  |  |  |  |  |
|  | | | |  | |  | | | | |  | | | | | | |  | | |  | | |  |  |  |  |  |  |
| Parameters  RunProcRSP Form118.txt CheckBoxTrans,CheckBox,0,False,03,False,Trans,False,,, ListBoxQuanti,ListBox,,True,200000000000_Outputs,True,,False,,, CheckBoxHist,CheckBox,0,True,300000000000_Charts,True,Histograms,False,,, OptionButtonHistBar,OptionButton,-1,True,300000000100_Charts,True,Bars,False,,, OptionButtonHistCont,OptionButton,0,True,300000010100_Charts,True,Continuous line,False,,, CheckBoxCum,CheckBox,0,True,300000000200_Charts,True,Cumulative histograms,False,,, OptionButtonHisBased,OptionButton,-1,True,300000000300_Charts,True,Based on the histogram,False,,, OptionButtonECDF,OptionButton,0,True,300000010300_Charts,True,Empirical cumulative distribution,False,,, CheckBoxRData,CheckBox,0,True,200000000008_Outputs,True,Resamples,False,,, TextBoxConfPer,TextBox,95,True,200000000003_Outputs,True,Confidence interval (%):,False,,, CheckBoxRStat,CheckBox,0,True,200000000007_Outputs,True,Resampled statistics,False,,, CheckBoxNormInt,CheckBox,0,True,200000000004_Outputs,True,Standard bootstrap interval,False,,, CheckBoxPercInt,CheckBox,0,True,200000000005_Outputs,True,Simple percentile interval,False,,, CheckBoxBiasInt,CheckBox,0,True,200000000006_Outputs,True,B.C. percentile interval,False,,, OptionButton_MVRemove,OptionButton,0,True,100000000100_Missing data,True,Remove the observations,False,,, OptionButton_MVEstimate,OptionButton,0,True,100000000300_Missing data,True,Estimate missing data,False,,, OptionButton_MeanMode,OptionButton,-1,True,100000000400_Missing data,True,Mean,False,,, OptionButtonAll,OptionButton,-1,True,100000000200_Missing data,True,For all samples,False,,, OptionButtonRestrict,OptionButton,0,True,100000010200_Missing data,True,For the corresponding sample,False,,, OptionButtonMVRefuse,OptionButton,-1,True,100000000000_Missing data,True,Do not accept missing data,False,,, OptionButton_W,OptionButton,0,True,000000000001_General,True,Workbook,False,,, OptionButton_R,OptionButton,-1,True,000000010001_General,True,Range,False,,, OptionButton_S,OptionButton,0,True,000000020001_General,True,Sheet,False,,, RefEdit_R,RefEdit0,'Sheet10'!$M$82,True,000000000101_General,True,Range:,False,,0,0 CheckBoxVarLabels,CheckBox,0,True,000000000201_General,True,Sample labels,False,,, CheckBox_W,CheckBox,0,True,000000000301_General,True,Weights,False,,, RefEdit_W,RefEdit,,True,000000000401_General,True,Weights:,False,,, TextBox_Resample,TextBox,1000,True,000000000000_General,True,Number of samples:,False,,, RefEdit_X,RefEdit0,'Sheet10'!$I$2:$I$81,True,000000000100_General,True,Quantitative data:,False,,26,1 ComboBoxMethod,ComboBox,0,True,000000010300_General,True,Method:,False,,, TextBox_obs,TextBox,5,True,000000000500_General,True,Sample size:,False,,, ComboBoxChartY,ComboBox,2,True,300000000101_Charts,True,Ordinate of the histograms:,False,,,  1 ListBox 118 ListBoxQuanti  1 22  Sum,0 Mean,-1 Variance (n),-1 Variance (n-1),-1 Standard deviation (n),-1 Standard deviation (n-1),-1 Median,-1 1st Quartile,0 3rd Quartile,0 Variation coefficient,0 Standard error of the mean,0 Mean absolute deviation,0 Median absolute deviation,-1 Geometric mean,0 Geometric standard deviation,0 Harmonic mean,0 1-Percentile,0 99-Percentile,0 2.5-Percentile,0 97.5-Percentile,0 5-Percentile,0 95-Percentile,0  670022 | | | | Estimator | | Estimator (Bootstrap) | | | | | Standard deviation (Bootstrap) | | | | | | |  | | |  | | |  |  |  |  |  |  |
| Mean | | | | 14.250 | | 14.256 | | | | | 0.237 | | | | | | |  | | |  | | |  |  |  |  |  |  |
| Variance (n) | | | | 4.438 | | 4.403 | | | | | 0.898 | | | | | | |  | | |  | | |  |  |  |  |  |  |
| Variance (n-1) | | | | 4.494 | | 4.459 | | | | | 0.909 | | | | | | |  | | |  | | |  |  |  |  |  |  |
| Standard deviation (n) | | | | 2.107 | | 2.087 | | | | | 0.216 | | | | | | |  | | |  | | |  |  |  |  |  |  |
| Standard deviation (n-1) | | | | 2.120 | | 2.100 | | | | | 0.218 | | | | | | |  | | |  | | |  |  |  |  |  |  |
| Median | | | | 15.000 | | 15.000 | | | | | 0.000 | | | | | | |  | | |  | | |  |  |  |  |  |  |
| Median absolute deviation | | | | 0.000 | | 0.000 | | | | | 0.000 | | | | | | |  | | |  | | |  |  |  |  |  |  |
|  | | | |  | |  | | | | |  | | | | | | |  | | |  | | |  |  |  |  |  |  |
| **Results of the resampling (OBS 2):** | | | | | | | |  | | | | | |  | | |  | | |  | | |  |  |  |  |  |  |  |
|  |  | |  | | | | |  | | | | | |  | | |  | | |  | | |  |  |  |  |  |  |  |
| Parameters | Estimator | | Estimator (Bootstrap) | | | | | Standard deviation (Bootstrap) | | | | | |  | | |  | | |  | | |  |  |  |  |  |  |  |
| Mean | 14.625 | | 14.624 | | | | | 0.190 | | | | | |  | | |  | | |  | | |  |  |  |  |  |  |  |
| Variance (n) | 2.984 | | 2.929 | | | | | 0.876 | | | | | |  | | |  | | |  | | |  |  |  |  |  |  |  |
| Variance (n-1) | 3.022 | | 2.966 | | | | | 0.888 | | | | | |  | | |  | | |  | | |  |  |  |  |  |  |  |
| Standard deviation (n) | 1.728 | | 1.691 | | | | | 0.262 | | | | | |  | | |  | | |  | | |  |  |  |  |  |  |  |
| Standard deviation (n-1) | 1.738 | | 1.702 | | | | | 0.263 | | | | | |  | | |  | | |  | | |  |  |  |  |  |  |  |
| Median | 15.000 | | 15.000 | | | | | 0.000 | | | | | |  | | |  | | |  | | |  |  |  |  |  |  |  |
| Median absolute deviation | 0.000 | | 0.000 | | | | | 0.000 | | | | | |  | | |  | | |  | | |  |  |  |  |  |  |  |
|  |  | |  | | | | |  | | | | | |  | | |  | | |  | | |  |  |  |  |  |  |  |
| **Mood test:** | | | | |  | |  | | |  | | |  | | |  | | |  | | | |  | | |  | |  |  |
|  | | | | |  | |  | | |  | | |  | | |  | | |  | | | |  | | |  | |  |  |
| U | | | | | 0.256 | |  | | |  | | |  | | |  | | |  | | | |  | | |  | |  |  |
| Critical value  RunProcRSP Form118.txt CheckBoxTrans,CheckBox,0,False,03,False,Trans,False,,, ListBoxQuanti,ListBox,,True,200000000000_Outputs,True,,False,,, CheckBoxHist,CheckBox,0,True,300000000000_Charts,True,Histograms,False,,, OptionButtonHistBar,OptionButton,-1,True,300000000100_Charts,True,Bars,False,,, OptionButtonHistCont,OptionButton,0,True,300000010100_Charts,True,Continuous line,False,,, CheckBoxCum,CheckBox,0,True,300000000200_Charts,True,Cumulative histograms,False,,, OptionButtonHisBased,OptionButton,-1,True,300000000300_Charts,True,Based on the histogram,False,,, OptionButtonECDF,OptionButton,0,True,300000010300_Charts,True,Empirical cumulative distribution,False,,, CheckBoxRData,CheckBox,0,True,200000000008_Outputs,True,Resamples,False,,, TextBoxConfPer,TextBox,95,True,200000000003_Outputs,True,Confidence interval (%):,False,,, CheckBoxRStat,CheckBox,0,True,200000000007_Outputs,True,Resampled statistics,False,,, CheckBoxNormInt,CheckBox,0,True,200000000004_Outputs,True,Standard bootstrap interval,False,,, CheckBoxPercInt,CheckBox,0,True,200000000005_Outputs,True,Simple percentile interval,False,,, CheckBoxBiasInt,CheckBox,0,True,200000000006_Outputs,True,B.C. percentile interval,False,,, OptionButton_MVRemove,OptionButton,0,True,100000000100_Missing data,True,Remove the observations,False,,, OptionButton_MVEstimate,OptionButton,0,True,100000000300_Missing data,True,Estimate missing data,False,,, OptionButton_MeanMode,OptionButton,-1,True,100000000400_Missing data,True,Mean,False,,, OptionButtonAll,OptionButton,-1,True,100000000200_Missing data,True,For all samples,False,,, OptionButtonRestrict,OptionButton,0,True,100000010200_Missing data,True,For the corresponding sample,False,,, OptionButtonMVRefuse,OptionButton,-1,True,100000000000_Missing data,True,Do not accept missing data,False,,, OptionButton_W,OptionButton,0,True,000000000001_General,True,Workbook,False,,, OptionButton_R,OptionButton,-1,True,000000010001_General,True,Range,False,,, OptionButton_S,OptionButton,0,True,000000020001_General,True,Sheet,False,,, RefEdit_R,RefEdit0,'Sheet10'!$M$110,True,000000000101_General,True,Range:,False,,0,0 CheckBoxVarLabels,CheckBox,0,True,000000000201_General,True,Sample labels,False,,, CheckBox_W,CheckBox,0,True,000000000301_General,True,Weights,False,,, RefEdit_W,RefEdit,,True,000000000401_General,True,Weights:,False,,, TextBox_Resample,TextBox,1000,True,000000000000_General,True,Number of samples:,False,,, RefEdit_X,RefEdit0,'Sheet10'!$J$2:$J$81,True,000000000100_General,True,Quantitative data:,False,,80,1 ComboBoxMethod,ComboBox,0,True,000000010300_General,True,Method:,False,,, TextBox_obs,TextBox,5,True,000000000500_General,True,Sample size:,False,,, ComboBoxChartY,ComboBox,2,True,300000000101_Charts,True,Ordinate of the histograms:,False,,,  1 ListBox 118 ListBoxQuanti  1 22  Sum,0 Mean,-1 Variance (n),-1 Variance (n-1),-1 Standard deviation (n),-1 Standard deviation (n-1),-1 Median,-1 1st Quartile,0 3rd Quartile,0 Variation coefficient,0 Standard error of the mean,0 Mean absolute deviation,0 Median absolute deviation,-1 Geometric mean,0 Geometric standard deviation,0 Harmonic mean,0 1-Percentile,0 99-Percentile,0 2.5-Percentile,0 97.5-Percentile,0 5-Percentile,0 95-Percentile,0  629472  Pharmacies n=80*SEP*Summary statistics*SEP*$M$121 Pharmacies n=80*SEP*Results of the resampling (X1)*SEP*$M$127 | | | | | 3.841 | |  | | |  | | |  | | |  | | |  | | | |  | | |  | |  |  |
| DF | | | | | 1.000 | |  | | |  | | |  | | |  | | |  | | | |  | | |  | |  |  |
| p-value | | | | | 0.634 | |  | | |  | | |  | | |  | | |  | | | |  | | |  | |  |  |
| alpha | | | | | 0.05 | |  | | |  | | |  | | |  | | |  | | | |  | | |  | |  |  |
| The p-value has been computed using 10000 Monte Carlo simulations. Time elapsed: 0s. | | | | | | | | | | | | | | | | | | |  | | | |  | | |  | |  |  |
|  | | | | |  | |  | | |  | | |  | | |  | | |  | | | |  | | |  | |  |  |
| Test interpretation: | | | | |  | |  | | |  | | |  | | |  | | |  | | | |  | | |  | |  |  |
| H0: The medians of OBS 1 and OBS 2 are equal. | | | | | | |  | | |  | | |  | | |  | | |  | | | |  | | |  | |  |  |
| Ha: Medians of OBS 1 and OBS 2 are not equal | | | | | | |  | | |  | | |  | | |  | | |  | | | |  | | |  | |  |  |
| As the computed p-value is greater than the significance level alpha=0.05,  RunProcRSP Form118.txt CheckBoxTrans,CheckBox,0,False,03,False,Trans,False,,, ListBoxQuanti,ListBox,,True,200000000000_Outputs,True,,False,,, CheckBoxHist,CheckBox,0,True,300000000000_Charts,True,Histograms,False,,, OptionButtonHistBar,OptionButton,-1,True,300000000100_Charts,True,Bars,False,,, OptionButtonHistCont,OptionButton,0,True,300000010100_Charts,True,Continuous line,False,,, CheckBoxCum,CheckBox,0,True,300000000200_Charts,True,Cumulative histograms,False,,, OptionButtonHisBased,OptionButton,-1,True,300000000300_Charts,True,Based on the histogram,False,,, OptionButtonECDF,OptionButton,0,True,300000010300_Charts,True,Empirical cumulative distribution,False,,, CheckBoxRData,CheckBox,0,True,200000000008_Outputs,True,Resamples,False,,, TextBoxConfPer,TextBox,95,True,200000000003_Outputs,True,Confidence interval (%):,False,,, CheckBoxRStat,CheckBox,0,True,200000000007_Outputs,True,Resampled statistics,False,,, CheckBoxNormInt,CheckBox,0,True,200000000004_Outputs,True,Standard bootstrap interval,False,,, CheckBoxPercInt,CheckBox,0,True,200000000005_Outputs,True,Simple percentile interval,False,,, CheckBoxBiasInt,CheckBox,0,True,200000000006_Outputs,True,B.C. percentile interval,False,,, OptionButton_MVRemove,OptionButton,0,True,100000000100_Missing data,True,Remove the observations,False,,, OptionButton_MVEstimate,OptionButton,0,True,100000000300_Missing data,True,Estimate missing data,False,,, OptionButton_MeanMode,OptionButton,-1,True,100000000400_Missing data,True,Mean,False,,, OptionButtonAll,OptionButton,-1,True,100000000200_Missing data,True,For all samples,False,,, OptionButtonRestrict,OptionButton,0,True,100000010200_Missing data,True,For the corresponding sample,False,,, OptionButtonMVRefuse,OptionButton,-1,True,100000000000_Missing data,True,Do not accept missing data,False,,, OptionButton_W,OptionButton,0,True,000000000001_General,True,Workbook,False,,, OptionButton_R,OptionButton,-1,True,000000010001_General,True,Range,False,,, OptionButton_S,OptionButton,0,True,000000020001_General,True,Sheet,False,,, RefEdit_R,RefEdit0,'Sheet10'!$M$138,True,000000000101_General,True,Range:,False,,0,0 CheckBoxVarLabels,CheckBox,0,True,000000000201_General,True,Sample labels,False,,, CheckBox_W,CheckBox,0,True,000000000301_General,True,Weights,False,,, RefEdit_W,RefEdit,,True,000000000401_General,True,Weights:,False,,, TextBox_Resample,TextBox,1000,True,000000000000_General,True,Number of samples:,False,,, RefEdit_X,RefEdit0,'Sheet10'!$K$2:$K$81,True,000000000100_General,True,Quantitative data:,False,,80,1 ComboBoxMethod,ComboBox,0,True,000000010300_General,True,Method:,False,,, TextBox_obs,TextBox,5,True,000000000500_General,True,Sample size:,False,,, ComboBoxChartY,ComboBox,2,True,300000000101_Charts,True,Ordinate of the histograms:,False,,,  1 ListBox 118 ListBoxQuanti  1 22  Sum,0 Mean,-1 Variance (n),-1 Variance (n-1),-1 Standard deviation (n),-1 Standard deviation (n-1),-1 Median,-1 1st Quartile,0 3rd Quartile,0 Variation coefficient,0 Standard error of the mean,0 Mean absolute deviation,0 Median absolute deviation,-1 Geometric mean,0 Geometric standard deviation,0 Harmonic mean,0 1-Percentile,0 99-Percentile,0 2.5-Percentile,0 97.5-Percentile,0 5-Percentile,0 95-Percentile,0  858984  Pharmacies n=80*SEP*Summary statistics*SEP*$M$149 Pharmacies n=80*SEP*Results of the resampling (X1)*SEP*$M$155  one cannot reject the null hypothesis H0. | | | | | | | | | | | | | | | | | | | | | | | | | | | |  |  |
|  |  |  |  |  |  |  |  |  |  |  |  |  |  |  |  |  |  |  |  |  |  |  |  |  |  |  |  |  |  |
